# Supplementary material for: Predicting the risk and speed of drug resistance emerging in soil-transmitted helminths during preventive chemotherapy
Source: Nat Commun. 2024 Feb 6;15:1099. doi: 10.1038/s41467-024-45027-2 (PMC10847116; doi:10.1038/s41467-024-45027-2)

Supplementary Information A: Technical model specification

Contents

This document 2

Introduction 3

Human demography 3

Transmission of infection 5

Individual contribution and exposure to the environmental reservoir 5

Acquisition of new worms by hosts 8

Within-host dynamics of infection 8

Dynamics of the environmental reservoir 10

Genetic mechanisms underlying drug resistance in worms 11

Model concepts for monogenic resistance 11

Model concepts for polygenic resistance 12

Within-host dynamics of parasite genetics 14

Dynamics of parasite genetics in the environmental reservoir 16

Genetic drift 16

Preventive chemotherapy 17

Individual participation 18

Parasitological effects of drug treatment 18

Drug efficacy monitoring 19

Survey output 19

Simulation algorithm 20

Examples 21

Running a simulation 21

Evolution of drug resistance 23

Initialising a simulation based on a previously saved population state 25

Comparison to WORMSIM 28

References 29

R session info 30

This document

This document is an appendix to the paper ***Predicting the risk and speed of drug resistance emerging in soil-transmitted helminths during preventive chemotherapy*** by Coffeng *et al.* (Nature Communications 2024) and provides a technical specification of an individual-based model (IBM) for the evolution of monogenic and polygenic drug resistance in soil-transmitted helminths (STH) during regular preventive chemotherapy (PC) or mass drug administration (MDA). Much of the model structure is based on the existing model WORMSIM, a generic individual-based modelling framework for transmission and control of worm infections in human, written in the Java programming language [1–8].

The IBM for drug resistance described here was developed in `R` using the `data.table` package and has been compiled into the open source package `simresist`, which can be used and distributed under the CC BY-NC 4.0 licence, citing the paper mentioned above.

Specific information about functions within the `simresist` package can be found in the `simresist` help files. For instance, `?simresist` will show the help file for the `simresist()` function which is used to perform a simulation, and `?param_mda` points to the default parameter values related to MDA.

This appendix was written in the `R markdown` language in `RStudio`, with hyperlinks formatted in blue. Plots were produced using the `ggplot2` package. See the section Session info for details about version numbers of all packages used to produce this document. Blocks of `R` code and output are printed against a grey background (e.g., in the Examples section), where "`#>`" indicates output that is printed to the console when the code is executed, for instance:

```
library(ggplot2)
library(simresist)
#> Loading required package: data.table
```

Introduction

The simresist package allows the user to simulate a dynamic population of human hosts who are born, age, die, and are exposed to eggs or larvae of soil-transmitted helminths (STH) present in the environment. Within each human host, simresist simulates the life histories of individual female and male worms that mate and produce eggs, which are excreted into the environment by the host. In addition, simresist simulates the control of STH by reductions in host exposure and contribution to the environmental reservoir (i.e., water, sanitation and hygiene, or WASH) and PC. The evolution of drug resistance as a result of PC is explicitly simulated, based on user assumptions about the genetic mechanisms underlying resistance and the initial genetic state of the worm population in the simulation. Simulation results are processed and returned in the form of a list of vectors and data.table objects that can be easily analysed and visualised in R.

The following sections describe the simulation processes in more detail. The order in which all of the simulation processes are executed is described at the end in the section Simulation algorithm. Where applicable, we explain what arguments of the simresist() function can be used to change default parameter values by naming the argument in **bold Courier fonts**. For instance, worms can only mate once they become patent at a certain age **w_patent**.

In the very last section, we provide comparison of model predictions by WORMSIM and simresist for a range of epidemiological and programmatic scenarios, demonstrating that simresist (with drug resistance turned off), reproduces the original WORMSIM model behaviour.

Human demography

Human hosts are assumed to live in a semi-closed population: individuals enter and leave the population by birth or death, and to keep the simulated population size more or less the same, individuals can randomly leave the population (e.g., emigration). The model does not consider immigration of individuals. The number of births N_b per time step δt in the simulation is drawn from a Poisson distribution with expectation defined as a function of age-specific annual fertility rates r_a (**fert**) and the number of women of reproductive age N_f in each of n_a age groups a:

$$N_b \sim \text{Poisson}(\delta t \cdot \sum_{a=1}^{n_a} N_{f,a} \cdot r_a) \tag{1}$$

Humans are assumed to be born female with 50% probability. Birth events are scheduled to occur uniformly distributed over the period covered by time step $t \to t + \delta t$.

Mortality is governed by drawing a random value for human lifespan T_H at birth of individual i, based on the cumulative survival probability (**surv_cum**) by age, the inverse cumulative survival function $S_H^{-1}(x)$, and a random value u_i drawn from a standard uniform distribution at birth:

$$\begin{aligned} T_{H,i} &= S_H^{-1}(u_i) \\ u_i &\sim \text{U}(0,1) \end{aligned} \tag{2}$$

Fertility and cumulative survival are defined using the same cut-offs for age (**age_table**).[1] Note that fertility rates are interpolated over age in a piece-wise constant fashion (i.e., **age_table** defines the lower boundary of age categories for fertility). In contrast, cumulative survival is interpolated over age in a piece-wise linear fashion. Table A1 shows the default parameter values used for fertility and cumulative survival. Figure A1 shows the resulting age distribution of the human population, which follows the age distribution in sub-Saharan Africa as estimated by the UN Population Division for the year 2000 [9].

[1] Note that **age_table**, **fert**, and **surv_cum** must be defined as vectors of equal length, **age_table** must contain strictly increasing values, and **surv_cum** must contain strictly decreasing values between zero and one.

What makes the population semi-closed (rather than fully closed) is that when the population reaches a user-defined size (**n_human_max**, which is 440 by default), a random fraction (**reap_fraction**, which is 0.05 by default) is removed from the simulation. This way, a more or less stable population size can be simulated (Figure A2). This process can be considered to represent that individuals leave the community and are effectively no longer members of the geographical transmission unit being simulated.

When starting a simulation, the initial demographic state of the human population is simulated. At the start of this so-called "warm-up" period, a default number of people (**n_human_init**, which is 400 by default) are created and a lifespan is drawn for each individual as described above. Then for each individual a current age is drawn from a uniform distribution with a range of zero and the lifespan drawn for that individual. Next, to achieve a stable human population and age distribution, we run the human demographic processes for a duration of 250 year(s) (**human_warmup_duration**), taking 12 discrete time step(s) per year (**human_warmup_steps**). Only after this initial warm-up period does `simresist` start simulation of transmission.[2]

Table A1: Parameter values for human demography.

Age	Fertilitate rate (per woman per year)	Cumulative survival (fraction)
0	0.000	1.000
5	0.000	0.804
10	0.000	0.772
15	0.109	0.760
20	0.300	0.740
30	0.119	0.686
50	0.000	0.509
90	0.000	0.000

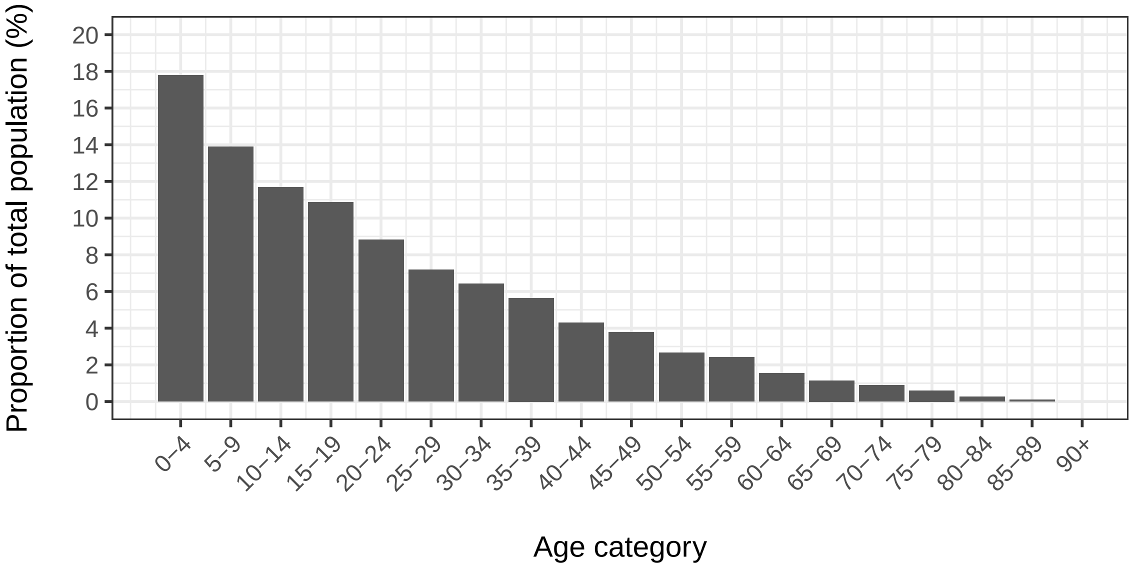

Figure A1: Age distribution of the simulated human population in equilibrium.

[2]If the user provides a pre-generated population state object via the `state_init` argument (e.g., a stored population state from a previous simulation with `simresist`), the warm-up period is skipped.

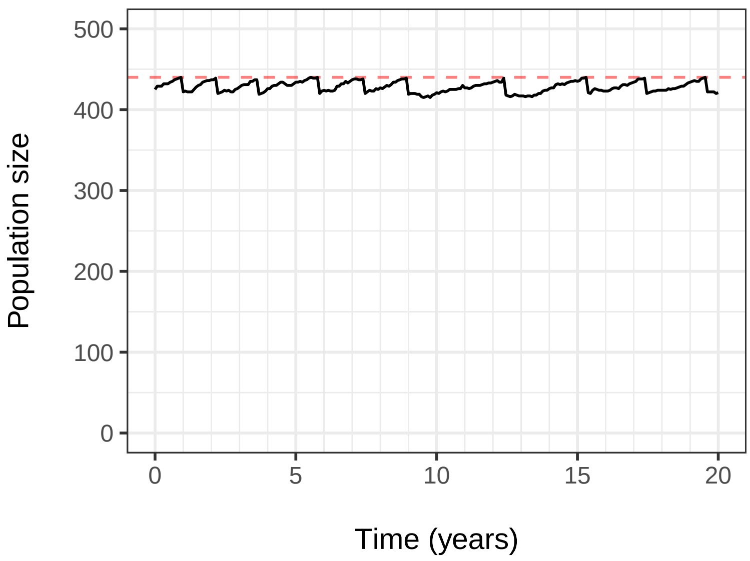

Figure A2: Simulated human population size over time. The saw-tooth pattern is due to a random fraction of people leaving each time the total population size reaches the user-defined maximum (red dashed line).

Transmission of infection

Transmission of STH infection is mediated via one central environmental reservoir to which all humans contribute and are exposured. The rate of contribution and exposure of specific individals varies with age and random personal factors. Although sex is an attribute of simulated humans, the model currently does not support sex-specific exposure and contribution of humans. The amount of infective material (larvae or eggs) that an individual host contributes to the environmental reservoir depends on the number and reproductive status of worms in that individual. The amount of infective material in the environmental reservoir is updated at each time step in the simulation based on 1) the total egg output of the host population and 2) a fixed fraction of the infective material already present in the reservoir that survives up to the next time point (depending on the average lifespan of eggs or larvae in the environment).[3] In the next subsections, we describe transmission-related processes in more details.

Individual contribution and exposure to the environmental reservoir

For each individual i, we define their relative contribution $Co_{i,t}$ and exposure $Ex_{i,t}$ to the environmental reservoir of infection at time t:

$$\begin{aligned} Co_{i,t} &= Coa_{i,t} \cdot Coi_i \cdot Coc_{i,t} \\ Ex_{i,t} &= Exa_{i,t} \cdot Exi_i \cdot Exc_{i,t} \end{aligned} \tag{3}$$

Each are each composed of three components:

1. an age component ($Exa_{i,t}$, $Coa_{i,t}$) that is updated as the individual ages

[3]Note that after warm-up of demography (which takes 12 time steps per year; `human_warmup_steps`), all processes, including transmission and human demography, are simulated at 52 time steps per year (`steps`).

2. a random component (Exi_i, Coi_i) that is fixed throughout life and reflects variation in individual behaviours and habits, and
3. the potential impact of control measures that reduce contribution and/or exposuse ($Exc_{i,t}$, $Coc_{i,t}$), which may change over time (e.g., WASH-related interventions).

Age-dependent relative contribution $Coa_{i,t}$ and exposure $Exa_{i,t}$ are each defined based on a linear interpolation of user-defined values of relative contribution and exposure (**`contr_age_y`** and **`expo_age_y`**) at specific ages (**`contr_age_x`** and **`expo_age_x`**). For relative exposure, the default age pattern varies with STH species (**`species`**), reflecting differences in the age patterns of infection levels. This is related to how hosts contract infection: geophagia for *Ascaris lumbricoides* vs. bare skin contact with infective material in the environment for *Necator americanus*. See Figure A3 for the default values of age-dependent exposure by species, which have also been used in previous modelling studies and have been validated against data on age-specific infection levels [1,2].

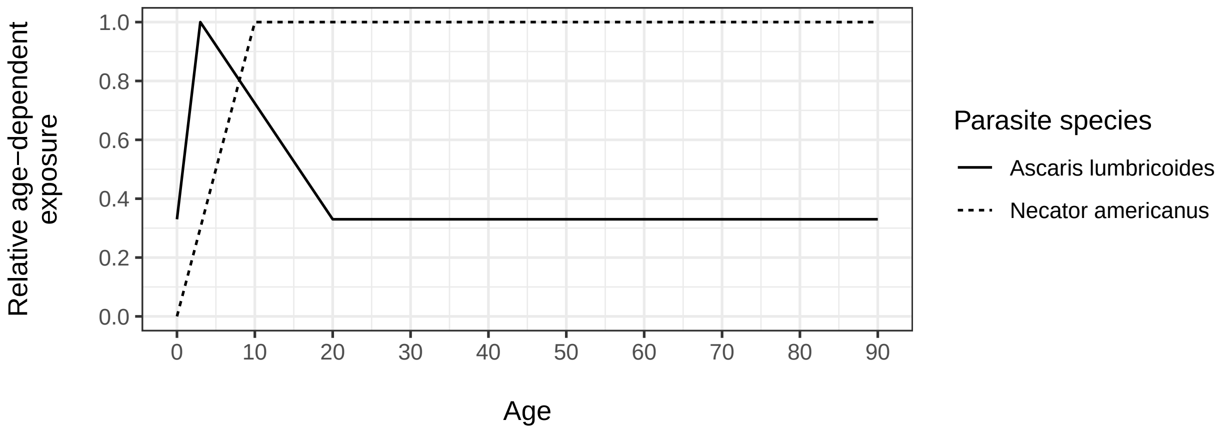

Figure A3: Age-dependent relative exposure of humans to the environmental reservoir of infection by worm species. Values have been scaled such that the maximum level equals 1, i.e., at the age of 4 for *Ascaris lumbricoides* and 10 and above for *Necator americanus*.

Age-dependent relative contribution to the reservoir is assumed to be the same for all STH species, as the causative behaviour (open defaecation) can be assumed to be identical. We assume that age-dependent contribution to the reservoir is similar to the age-dependent exposure pattern for hookworm infection, as this requires bare skin contact with free-living larvae in the environment and therefore can be considered a close proxy for the amount of time an individual spends in contaminated areas (in contrast to *Ascaris lumbricoides* infection levels, which are driven more so by geophagia). See Figure A4 for the default values of age-dependent contribution, which have been used in previous modelling studies [1–8].

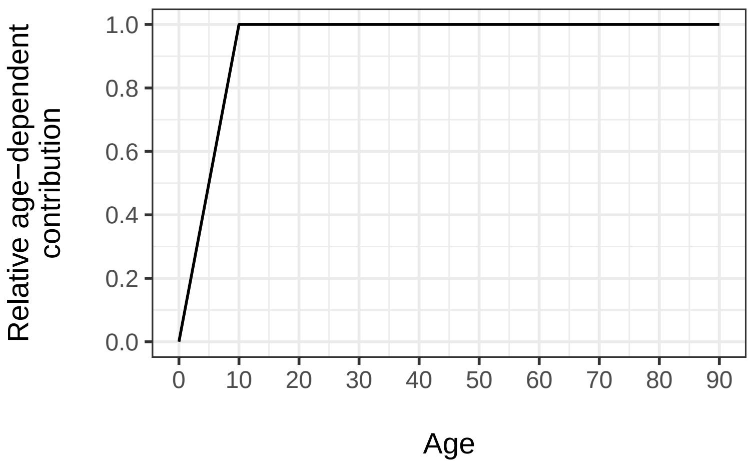

Figure A4: Age-dependent relative contribution of humans to the environmental reservoir of infection

The lifelong random components Coi_i and Exi_i are defined to be the same ($Coi_i = Exi_i$), assuming that individual behaviours related to contribution and exposure to the reservoir are highly correlated. For each individual, at birth a value of $Coi_i = Exi_i$ is drawn from a Gamma distribution with mean one and default shape (and rate) of 0.4 (**`contrib_k`**). The value of this shape parameter lies within the range of values reported in literature [10] and used in previous modelling studies [1–8]. The shape parameter governs the level of overdispersion of parasites in the host population (in addition to variation in exposure by age). The lower the value of the shape parameter (value must be > 0), the more overdispersed the parasite population is.

Based on model concepts from a previous modelling study [3], which we simplify somewhat here, the impacts $Coc_{i,t}$ and $Exc_{i,t}$ of control measures are defined as:

$$\begin{aligned} Coc_{i,t} &= 1 - (\alpha_{Co,t} \cdot \beta_{i,t}) \\ Exc_{i,t} &= 1 - (\alpha_{Ex,t} \cdot \beta_{i,t}) \end{aligned} \tag{4}$$

Here, $\alpha_{Co,t}$ and $\alpha_{Ec,t}$ represent the reduction in contribution (**`contr_eff`**) and exposure (**`expo_eff`**) in case an individual takes up the control measure at time t, and $\beta_{i,t}$ is an indicator of whether or not individual i has taken up the intervention at time t:

$$\beta_{i,t} = \begin{cases} 0 & \text{if } ec_i < 1 - \text{Cov}_t \text{ or individual } i \text{ is not part of the target age group,} \\ 1 & \text{if } ec_i \geq 1 - \text{Cov}_t \text{ and individual } i \text{ is part of the target age group.} \end{cases} \tag{5}$$

Here, ec_i is a lifelong value that is drawn from a uniform distribution $\text{U}(0, 1)$ at birth and that represents the individual's inclination to take up control measures related to contribution and exposure to the environmental reservoir. This value is lifelong and is assigned at birth. Further, coverage Cov_t indicates the proportion of the targeted age group that takes up the intervention at time t (**`expo_contr_uptake`**). The age group targeted by the control intervention (**`expo_contr_age`**) is defined in terms of an lower and upper age bound, which are fixed for the duration of the entire simulation (e.g., 5 and 15 to indicate school age children).

The uptake and impact of control measures that reduce contribution and exposure to the environmental reservoir are allowed to change over time in a piece-wise constant fashion, based on the specification of time points (**`expo_contr_t`**) at which either or both of these values change.[4]

[4] Note that **`expo_contr_t`**, **`expo_contr_uptake`**, **`expo_eff`**, and **`contr_eff`** must be therefore defined as vectors of equal length.

Acquisition of new worms by hosts

We define the total force of infection FOI acting on the human population during time step $t \rightarrow t + \delta t$ as a function of the amount of infective material in the environmental reservoir Res_t at time t, the overall transmission rate ζ (**`zeta`**), and the average relative exposure levels $Ex_{i,t}$ of the $N_{H,t}$ individuals in the human population at time t:

$$FOI_{t \rightarrow t+\delta t} = \delta t \cdot \zeta \cdot Res_t \cdot \frac{1}{N_{H,t}} \sum_{i=1}^{N_{H,t}} Ex_{i,t} \tag{6}$$

The number of new parasites W_b (subscript b for "birth") that establish themselves in the host population during time step $t \rightarrow t + \delta t$ is drawn from a Poisson distribution:

$$W_{b,t \rightarrow t+\delta t} \sim \text{Poisson}(FOI_{t \rightarrow t+\delta t}) \tag{7}$$

The hosts for the new worms are randomly sampled with replacement such that multiple worms may establish themselves in the same host during the same time step $t \rightarrow t+\delta t$. The probability that individual i is selected for a new worm to establish itself in is proportional to the individual's relative exposure $Ex_{i,t}$ at time t. The probability that a new worm is female is assumed to be 50%. Worm establishment events are scheduled to occur uniformly distributed over the period $t \rightarrow t + \delta t$.

Within-host dynamics of infection

Worm lifespan T_W is assumed to follow a Weibull distribution with shape k_{T_W} (**`w_lifespan_k`**) and mean μ_{T_W} (**`w_lifespan_mu`**). When a worm j establishes itself in a human host, its lifespan $T_{W,j}$ is generated using the inverse cumulative Weibull distribution function, which is parameterised in terms of shape k_{T_W} and scale λ_{T_W}:

$$\begin{aligned} T_{W,j} &= \lambda_{T_W} (-\ln(1 - z_j))^{1/k_{T_W}} \\ \lambda_{T_W} &= \frac{\mu_{T_W}}{\Gamma(1 + 1/k_{T_W})} \\ z_j &\sim \text{U}(0,1) \end{aligned} \tag{8}$$

Here, $\Gamma(.)$ is the gamma function (i.e., not the gamma distribution).

Default values for the distribution of parasite lifespan are shown in Table A2. The assumed mean parasite lifespan was based on historical studies [11,12]. The shape of the Weibull distribution was chosen to represent a linear increase in the risk of parasite mortality with parasite age.[5] These values have also been used in previous modelling exercises [1–8].

After establishment, parasites must reach a certain age (**`w_patent`**) to become patent before they can mate (Table A2) [1–8]. At each time step $t \rightarrow t + \delta t$, patent female worms are considered fertilised as long as at least one patent male worm is present in the same host at time t. The total egg production $E_{W,i}$ by the female parasite population in host i is assumed to be negatively density-dependent on the total number N_{W_f} of fertilised patent female worms, using a hyperbolic saturating function (dropping subscript t for convenience):

[5]We consider a Weibull distribution for lifespan more realistic than the exponential distribution which assumes that the risk of a parasite dying is constant throughout its lifetime. Compared to a Weibull distribution, an exponential distribution implies that parasites start dying shortly after they establish themselves, and that a minority of the parasites have extremely long lifespans. Setting the shape parameter of the Weibull distribution to $k = 1.0$ results in an exponential distribution. Setting the shape parameter to a value $k > 1.0$ implies that the risk of a parasite dying increases as a polynomial of time with power $k - 1$ (hence, $k = 2$ implies a linear increase).

Table A2: Adult parasite lifespan and pre-patent period by species.

Species	Adult parasite lifespan (years)				Pre-patent period (weeks)
	Mean	Shape	Scale (derived)	95%-CI	
Ascaris lumbricoides	1	2	1.13	0.18 - 2.17	10
Necator americanus	3	2	3.39	0.54 - 6.50	7

$$\begin{aligned} E_{W,i} &= c \cdot c_i \cdot \frac{a \cdot N_{W_f}}{1 + a \cdot N_{W_f}/b} \\ c_i &\sim \Gamma(k_c, k_c) \end{aligned} \tag{9}$$

Here, a represents the relative egg production per fertilised female worm in absence of density-dependence,[6] b is the maximum potential relative egg production by a population of female worms in a single host, and c_i represents the relative suitability of host i for female worms to produce eggs in. The value of c_i is drawn per host i at host birth from a gamma distribution with shape and rate k_c (**`suitable_k`**) (and therefore mean 1.0) and is a lifelong value. Although seemingly trivial, the multiplier c is specified to disentangle transmission dynamics from:

1. the measurement unit that parameters a and b are defined in (e.g., egg counts or eggs per gram faeces)
2. the type of diagnostic test that was used to generate the original data that informed a and b, and
3. the weight w_s (**`w_sample`**) of faecal samples based on which model-predicted egg counts should be generated (see also the section Survey output).

This is done by defining the input values a_{input}, b_{input}, and c_{input} (**`egg_prod_a`**, **`egg_prod_b`**, **`egg_prod_c`**),[7] and transforming these as follow:

$$\begin{aligned} a &= a_{\text{input}}/b_{\text{input}} \\ b &= 1 \\ c &= c_{\text{input}} \cdot b_{\text{input}} \cdot w_s \end{aligned} \tag{10}$$

Table A3 provides an overview of the default parameter values for egg production by parasite species. The default value of $k_c = \infty$ (**`suitable_k`**) implies that there is no variation in host suitability. Previous modelling studies have worked with the assumption that $k_c = 50$ [1–8]. For the current study we chose to simplify this assumption as at this point we do not expect it to contribute to better understanding the evolution of drug resistance.

Table A3: Parameter values for egg production by female worms by species.

Species	a_{input}	b_{input}	c_{input}	k_c
Ascaris lumbricoides	9729	18675	1	∞
Necator americanus	200	1500	1	∞

[6] The first derivative of the function in equation (9) with respect to N_{W_f} is $c \cdot c_i \cdot \frac{ab^2}{a^2 N_{W_f}^2 + 2abN_{W_f} + b^2}$, which evaluates to $c \cdot c_i \cdot a$ when $N_{W_f} = 0$.

[7] The unit of a_{input} and b_{input} (`egg_prod_a`, `egg_prod_b`) should be identical.

Next, we define two types of egg output, one for the purpose of modelling transmission ($E_{W,i,\text{transmission}}$, which does not depend on the measurement unit of parameters a and b) and another for the purpose of simulating observed egg counts for survey output ($E_{W,i,\text{survey}}$):

$$
\begin{aligned}
E_{W,i,\text{transmission}} &= c_i \cdot \frac{a \cdot N_{W_f}}{1 + a \cdot N_{W_f}/b} \\
c_i &\sim \Gamma(k_c, k_c)
\end{aligned}
\tag{11}
$$

$$E_{W,i,\text{survey}} = c \cdot E_{W,i,\text{transmission}}$$

The use of $E_{W,i,\text{transmission}}$ is further explained in the next section on the dynamics of the environmental reservoir. For information about how $E_{W,i,\text{survey}}$ is used, see the section on survey output.

Dynamics of the environmental reservoir

The total amount of infective material $E_{\text{excreted},t \to t+\delta t}$ excreted by the human population into the environment during time step $t \to t + \delta t$ is defined as a function of the egg production by female worms $E_{W,i,t,\text{transmission}}$ in each host i at time t, the rate $Co_{i,t}$ at which individual host i contributes to the environmental reservoir at time t (see the section on contribution and exposure to the reservoir), and time step size δt:

$$E_{\text{excreted},t \to t+\delta t} = \delta t \cdot \sum_{i=1}^{N_{H,t}} E_{W,i,t,\text{transmission}} \cdot Co_{i,t} \tag{12}$$

The amount of infective material in the environment Res_t is then updated to $Res_{t+\delta t}$, given that a fraction of infective material will survive time period δt:

$$Res_{t+\delta t} = e^{-\delta t \cdot \psi} \cdot Res_t + E_{\text{excreted},t \to t+\delta t} \tag{13}$$

Here, parameter ψ (**`psi`**) is one divided by average lifespan of infective material in the environment (assuming exponential survival), which varies by species (Table A4).[8]

The initial amount of infectious material in the environmental reservoir (i.e., after warming up human demography) is set to:[9]

$$Res_{t=0} = \frac{1}{1 - e^{-\delta t \cdot \psi}} \sum_{i=1}^{N_{H,t}} E_{W,i,t,\text{transmission}} \cdot Co_{i,t} \tag{14}$$

Table A4: Average lifespan of infective material in the environmental reservoir.

Species	Average lifespan (months)
Ascaris lumbricoides	1.5
Necator americanus	0.46

[8]Note that the time unit of ψ (**`psi`**) is years, i.e., the unit of time used in the simulation.
[9]This step is skipped if the simulation is initiated with a previously stored simulation state (**`state_init`**). For an example, see the examples section.

Genetic mechanisms underlying drug resistance in worms

As explained in the main manuscript, we consider that the genetic mechanism(s) underlying drug resistance may be monogenic (one locus in the genome determines resistance), polygenic (many loci in the genome determine resistance, with each locus contributing similarly to resistance), or a mix of the two where one locus in the genome is much more influential than others. To accommodate this spectrum of possibilities, we implemented model concepts for 1) Mendelian inheritance of monogenic drug resistance via a single locus and 2) a quantitative trait representing polygenic drug resistance. These two model concepts can optionally be mixed together. In general, we assumed that neither monogenic nor polygenic resistance imposes a fitness cost.

Model concepts for monogenic resistance

We define monogenic drug resistance as resistance conferred by an allele on a single autosomal locus (i.e., not on a sex chromosome) for which a single (group) of allele variant(s) A exists which confers drug resistance; A can consist of either a single allele variant or a group of similar alleles that each confer the same trait. All allele variants that do not confer resistance are referred as variant a. We assume that no spontaneous mutations from a to A or vice versa occur over time.

We assign each worm j a genotype $N_{A,j} \in \{0, 1, 2\}$ which represents the number of copies of the resistance-conferring allele A a worm carries (i.e., 0, 1, or 2, which correspond to genotypes aa, aA, and AA, respectively). The probability that offspring o produced by a female and male worm with genotypes $N_{A,\mathrm{f}}$ and $N_{A,\mathrm{m}}$ has the genotype $N_{A,o} \in \{0, 1, 2\}$ is then:

$$\begin{aligned}
\Pr(N_{A,o} = 0) &= q_\mathrm{f} \cdot q_\mathrm{m} \\
\Pr(N_{A,o} = 1) &= p_\mathrm{f} \cdot q_\mathrm{m} + q_\mathrm{f} \cdot p_\mathrm{m} \\
\Pr(N_{A,o} = 2) &= p_\mathrm{f} \cdot p_\mathrm{m}
\end{aligned} \tag{15}$$

where:

$$\begin{aligned}
p_j &= N_{A,j}/2 \\
q_j &= 1 - p_j
\end{aligned} \tag{16}$$

Each of the three possible genotypes $N_{A,j} \in \{0, 1, 2\}$ is linked to a phenotype p_kill in terms of the probability that a worm is killed by the drug (**`pheno_kill`**).[10] Here, the penetrance of the phenotype can be classified as recessive, co-dominant, or dominant. In case of recessive penetrance, worms with $N_A = 2$ have a lower probability of being killed by the drug compared to worms with $N_{A,j} \in \{0, 1\}$. In case of co-dominant penetrance, the probability of a worm being killed by the drug decreases with the number of alleles $N_{A,j}$. This means that heterozygous worms ($N_{A,j} = 1$) or more likely to survive drug treatment compared to worms with $N_{A,j} = 0$, but not as much as worms with ($N_{A,j} = 2$). Last, in case of dominant penetrance, the probability of a worm being killed is lower for worms with at least one resistance-conferring allele ($N_{A,j} \in \{1, 2\}$) compared to worms with $N_{A,j} = 0$, with no difference between worms with $N_{A,j} = 1$ and $N_{A,j} = 2$. Optionally, each of the three genotypes $N_{A,j} \in \{0, 1, 2\}$ can be linked to a potential fitness cost in terms of the relative egg production of female worms (**`pheno_fitness`**, which is set to 1.0 by default). This fitness cost is multiplied with each female worms' contribution towards the overall egg production in a host.

At the start of a simulation, the distribution of genotypes is initialised given the initial frequency p_snp of the resistance-conferring allele (**`p_SNP`**).[11] For this initialisation, we randomly assign genotypes assuming that

[10] In case inheritance of drug resistance is assumed to be a mix of monogenic and polygenic, phenotypes for the monogenic component of drug resistance represent the probability that a worm is killed by the drug if its trait value $x_\mathrm{trait} = 0$. See also the secion on model concepts for polygenic resistance.

[11] SNP = single nucleotide polymorphism.

the genotype frequency in both the adult parasite population and the environmental reservoir is in genetic equilibrium according to Hardy-Weinberg (HW) equilibrium: $p_{\mathrm{snp}}^2 + 2p_{\mathrm{snp}}(1 - p_{\mathrm{snp}}) + (1 - p_{\mathrm{snp}})^2 = 1$.[12]

Model concepts for polygenic resistance

To apply the quantitative trait model to polygenic drug resistance, we assign each worm a quantitative trait for their response to drug exposure, defined as the change in the log-odds of being killed by the drug (relative to a parasite with a trait value of zero). We assume that loci on the worm genome associated with polygenic drug resistance are mostly situated on autosomal chromosomes (i.e., they are not associated with worm sex) and constitute a very large, though finite set. This allows for certain simplifications relative to an explicit model for Mendelian inheritance of many alleles.

First, let us consider that the quantitative trait is mediated by an infinite number of genes. In this scenario, there are infinitely many genotypes and phenotypes, so according to the central limit theorem, trait values in a population follow a normal distribution. Now, in a more realistic scenario where the trait is mediated by a large but finite number of genes, let n be the number of involved loci with each two possible allele variants A and a. Here, the coefficients of the binomial expansion of $(A + a)^{2n}$ gives the frequency of distribution of all n allele combinations. Already for a moderate number of loci (e.g., $n = 10$) and allele frequencies not far from 0.5, the coefficients of the binomial expansion (i.e., $\binom{2n}{k}$ for each integer k in the set zero through $2n$) resemble a normal distribution (Figure A5). In scenarios with relatively rare alleles (i.e., frequencies under 0.5), the frequency distribution of genotypes will be relatively more skewed, and will only start to resemble a normal distribution when relatively more loci are involved.

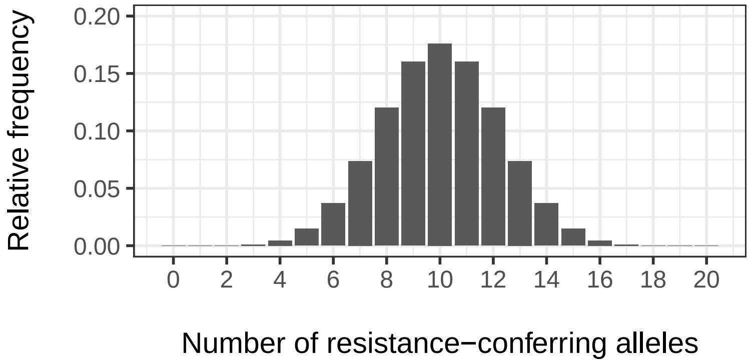

Figure A5: Relative frequency of diploid genotypes that include different numbers of resistance-conferring alleles across among 10 loci, based on the coefficients of the binomial expansion $\sum_{k=0}^{20} \binom{20}{k}$, assuming a resistance-conferring allele frequency of 50% for each locus.

Now, for our model concepts for polygenic inheritance, we assume that polygenic drug resistance is a quantitative trait that is governed by $\gg 10$ loci and is normally distributed in the population. Assuming that alleles have (mostly) additive effects, the expected trait value of an individual can be considered to be the mean of the genetic component of its two parents' trait values. We express the overall trait variance in a population in genetic equilibrium in terms of variance σ_{gen}^2 due to genetic factors (e.g., mutations and recombinations of parental chromosomes during meiosis), variance σ_{env}^2 due to environmental factors that can be considered (relatively) fixed over an individual's lifetime (e.g., epigenetic factors such as DNA methylation), and temporal variance σ_{t}^2 due to environmental factors that change with time.

[12]If the user wants to define an initial population state that is not in (HW) equilibrium, they can extract a population state object from an existing simulation, manully adapt the genotype frequencies, and then use this adapted object as the initial state for a new simulation.

Let us consider an initial founding generation g with a quantitative trait $x_{\text{trait},g}$ that follows a normal distribution with mean μ_g, genetic variance σ^2_{gen}, and non-genetic variance $\sigma^2_{\text{env}} + \sigma^2_{\text{t}}$. The genetic component $x_{\text{gen},g}$ of the quantitive trait, the lifelong fixed component $x_{\text{env},g}$ (including environmental noise), and the full trait $x_{\text{trait},g}$ (i.e., including all non-genetic and temporal components) can be assumed to follow normal distributions (in terms of mean and variance):

$$\begin{aligned} x_{\text{gen},g} &\sim \text{N}(\mu_g, \sigma^2_{\text{gen}}) \\ x_{\text{env},g} &\sim \text{N}(x_{\text{gen},g}, \sigma^2_{\text{env}}) \\ x_{\text{trait},g} &\sim \text{N}(x_{\text{env},g}, \sigma^2_{\text{t}}) \end{aligned} \tag{17}$$

Recognising that $x_{\text{gen},g}$ and $x_{\text{env},g}$ are random variables, using Bayes' Theorem, the three normal distributions can be combined into a single marginal distribution of the full trait:

$$x_{\text{trait},g} \sim \text{N}(\mu_g, \sigma^2_{\text{gen}} + \sigma^2_{\text{env}} + \sigma^2_{\text{t}}) \tag{18}$$

Now let us consider the next generation $g + 1$. The distributions of the quantitative trait in this next generation is then:

$$x_{\text{trait},g+1} \sim \text{N}(\mu_{g+1}, \sigma^2_{\text{gen}} + \sigma^2_{\text{env}} + \sigma^2_{\text{t}}) \tag{19}$$

Here, μ_{g+1} is the mean of the genetic component of the trait of parent pairs in generation g, where both parents' trait values $x_{\text{gen},g}$ originate from the same distribution (equation (17)):

$$\mu_{g+1} = \frac{x_{\text{gen},g,\text{father}} + x_{\text{gen},g,\text{mother}}}{2} \tag{20}$$

Now, to define a marginal distribution for the trait values in the next generation $g + 1$, we first define the distribution of the mean genetic component of parent pairs' traits μ_{g+1}. Assuming that the trait does not affect partner selection or reproductive fitness, the variance of the mean genetic component of parent pairs' traits can be defined as (dropping the subscript "father" and "mother" for convenience):

$$\text{Var}(\frac{x_{\text{gen},g} + x_{\text{gen},g}}{2}) = \frac{\text{Var}(x_{\text{gen},g} + x_{\text{gen},g})}{4} = \frac{\text{Var}(x_{\text{gen},g}) + \text{Var}(x_{\text{gen},g})}{4} = \frac{2\sigma^2_{\text{gen}}}{4} = \frac{\sigma^2_{\text{gen}}}{2} \tag{21}$$

Now, we can describe the parent mean trait μ_{g+1} for generation $g + 1$ as a random variable:

$$\mu_{g+1} \sim \text{N}(\mu_g, \frac{\sigma^2_{\text{gen}}}{2}) \tag{22}$$

Again, using Bayes' Theorem, equations (19) and (22) can be combined to described the marginal trait distribution in generation $g + 1$:

$$x_{\text{trait},g+1} \sim \text{N}(\mu_g, \sigma^2_{\text{gen}} + \frac{\sigma^2_{\text{gen}}}{2} + \sigma^2_{\text{env}} + \sigma^2_{\text{t}}) \tag{23}$$

For the nth generation since generation g, the trait distribution then becomes:

$$x_{\text{trait},g+n} \sim \text{N}(\mu_g, \sigma^2_{\text{gen}} + \frac{\sigma^2_{\text{gen}}}{2} + \frac{\sigma^2_{\text{gen}}}{4} + \frac{\sigma^2_{\text{gen}}}{8} + \cdots + \frac{\sigma^2_{\text{gen}}}{2^n} + \sigma^2_{\text{env}} + \sigma^2_{\text{t}}) \tag{24}$$

For a population in genetic equilibrium (i.e., $n \to \infty$), and given that $\sum_{n=0}^{\infty} \frac{1}{2^n} = 2$, the above can be simplified to:

$$x_{\text{trait},g+n} \sim \text{N}(\mu_g, 2\sigma^2_{\text{gen}} + \sigma^2_{\text{env}} + \sigma^2_{\text{t}}) \tag{25}$$

In other words, the overall trait variance in a population at genetic equilibrium is twice the variance due to genetic factors plus any environmental (or epigenetic) and temporal noise. Although we have ignored the possibility of genetic drift in the population mean trait value μ_{g+n} due to the population being of finite size, the analytical solution for the overall trait variance still holds in case of genetic drift, as the latter only causes a shift in the population trait values, and does not increase trait variance within what can be considered a single, homogeneously mixing population. See also the section on genetic drift and specifically Figure A6 in it.

Given equation (25), we parameterise our quantitative trait model for polygenic drug resistance in terms of the total trait variance $\sigma^2_{\text{trait}} = 2\sigma^2_{\text{gen}} + \sigma^2_{\text{env}} + \sigma^2_{\text{t}}$ in a population at equilibrium (**`trait_var`**); trait heritability among offspring $h^2 = \sigma^2_{\text{gen}}/(\sigma^2_{\text{gen}} + \sigma^2_{\text{env}} + \sigma^2_{\text{t}})$ (**`trait_h2`**),[13] which represents the proportion of variation in offspring trait values due to genetic factors (note that the factor 2 is dropped on purpose here); and the relative contribution of temporal variation to non-genetic variation $p_{\text{trait,t}} = \sigma^2_{\text{t}}/(\sigma^2_{\text{t}} + \sigma^2_{\text{env}})$ (**`trait_prop_t`**). With these parameters, all the basic individual-level processes of the quantitative trait model are quantified based on the following identities for σ^2_{trait}, σ^2_{env}, and σ^2_{t}:

$$\begin{aligned} \sigma^2_{\text{gen}} &= \frac{\sigma^2_{\text{trait}} \cdot h^2}{1 + h^2} \\ \sigma^2_{\text{env}} &= (1 - p_{\text{trait,t}}) \cdot (\sigma^2_{\text{trait}} - 2\sigma^2_{\text{gen}}) \\ \sigma^2_{\text{t}} &= \sigma^2_{\text{trait}} - 2\sigma^2_{\text{gen}} - \sigma^2_{\text{env}} \end{aligned} \tag{26}$$

In the default quantification of the model, polygenic inheritance of drug resistance is turned off ($h^2 = 0$ and $\sigma^2_{\text{trait}} = 0$). Further, in the current simulation study we assume that $p_{\text{trait,t}} = 1$, which effectively means that $\sigma^2_{\text{env}} = 0$ for all scenarios. If $h^2 = 0$ and $\sigma^2_{\text{trait}} > 0$, random non-inheritable noise is added to the quantitative trait (i.e., drug efficacy). In that case, $p_{\text{trait,t}}$ determines to what extent this noise varies ($p_{\text{trait,t}}$ closer to 1) or is fixed over an individual parasite's lifespan ($p_{\text{trait,t}}$ closer to 0).

At the start of a simululation, the trait distribution is initialised given heritability h^2 (**`trait_h2`**), variance σ^2_{trait} of the quantitative trait in the general population (**`trait_var`**), and the relative contribution of temporal variation to non-genetic trait variation $p_{\text{trait,t}}$ (**`trait_prop_t`**). For this initialisation, we randomly assign trait values assuming that the trait distribution in both the adult parasite population and the environmental reservoir is in equilibrium (equation (25)). This is done by assigning each worm j three values:

1. a genetic component $x_{\text{gen},j} \sim \text{N}(0, 2\sigma^2_{\text{gen}})$,
2. a lifelong component $x_{\text{env},j} \sim \text{N}(x_{\text{gen},j}, \sigma^2_{\text{env}})$ that includes the genetic component plus enviromental noise which is drawn when a worm establishes itself in a host, and
3. the full quantitative trait $x_{\text{trait},j} \sim \text{N}(x_{\text{env},j}, \sigma^2_{\text{t}})$, which includes the lifelong component plus temporal noise that is redrawn at every timestep in the simulation.

Infective material in the environment is only assigned a random genetic component $x_{\text{gen,ER}} \sim \text{N}(0, 2\sigma^2_{\text{gen}})$ as the non-genetic trait components are irrelevant for this parasite lifestage.

Within-host dynamics of parasite genetics

To simulate the population dynamics of monogenic and polygenic drug resistance (each on their own or a combination of the two), we assign each adult worm j the following five attributes:

[13] Note that in the definition of h^2, σ^2_{gen} in the denominator is *not* multiplied by 2 because it refers to trait variation in offspring and not the general population. This is in contrast to the definition of trait variation σ^2_{trait} in the general popualation, for which σ^2_{gen} *is* multiplied by 2.

1. a genetic component $x_{\text{gen},j} \in \mathbb{R}$,
2. a lifelong component $x_{\text{env},j} \in \mathbb{R}$ that includes the genetic component plus enviromental and/or epigenetic noise,
3. the full quantative trait $x_{\text{trait},j} \in \mathbb{R}$ that includes the lifelong component plus temporal noise,
4. a genotype $N_{A,j} \in \{0, 1, 2\}$ for monogenic drug resistance, and
5. a phenotype $0 < p_{\text{kill},j} < 1$ for the probability that worm j is killed by the drug if its quantitative trait $x_{\text{trait},j} = 0$.

For each new worm that establishes itself in a host, we randomly sample an infectious particle o from the environmental reservoir (with replacement). The particle o contains the following genetic information for the offspring of a random male-female worm pair that mated at some point in time:

1. the mean of the genetic trait components $\mu_{\text{gen},o}$ of the male-female worm pair, and
2. a vector of length 2 with the parental genotypes $N_{A,\text{f}}$ and $N_{A,\text{m}}$.

From this particle o, we generate the quantitative trait and genotype for a new worm j as follows:

$$\begin{aligned} x_{\text{gen},j} &\sim \text{N}(\mu_{\text{gen},o}, \sigma^2_{\text{gen}}) \\ x_{\text{env},j} &\sim \text{N}(x_{\text{gen},j}, \sigma^2_{\text{env}}) \\ x_{\text{trait},j} &\sim \text{N}(x_{\text{env},j}, \sigma^2_{\text{t}}) \\ N_{A,j} &\sim \text{Multinomial}(N_A = \{0, 1, 2\}, \Pr = p_{\text{genotype},o}) \end{aligned} \tag{27}$$

where $p_{\text{genotype},o}$ is determined by the parental genotypes $N_{A,\text{f}}$ and $N_{A,\text{m}}$ according to equations (15) and (16). Based on the drawn offspring genotype $N_{A,j}$ and the definition of phenotypes p_{kill} (**`pheno_kill`**) associated with each genotype, we assign the new worm a provisional phenotype $p^*_{\text{kill},j}$ (i.e., conditional on $x_{\text{trait},j} = 0$). When drug treatment is simulated, this provisional phenotype $p^*_{\text{kill},j}$ is used to calculate the probability of worm being killed by treatment ($p_{\text{kill},j}$, without an asterisk), accounting for the time-varying value of $x_{\text{trait},j}$ (for details, see the section on the parasitological effects of treatment).

During a time step $t \to t + \delta t$, we assume that a patent female worm j mates with one random patent male worm present in the same host (if any are present). A patent male worm is assumed to be able to mate with multiple patent female worms during the same time step. Eggs produced by female worm j in host i during time step $t \to t + \delta t$ are then assigned the following genetic information (i.e., same format as used for particles of infective material in the environment):

1. the mean of the genetic trait components $\mu_{\text{gen},ij}$ of the female worm and the male worm she mated with during time step $t \to t + \delta t$, and
2. a vector with the two genotypes $N_{A,\text{f}}$ and $N_{A,\text{m}}$ of the female worm and the male worm she mated with during time step $t \to t + \delta t$.

This genetic information is supplemented with a number $O_{ij,t}$ representing the contribution of female worm j to the total number of eggs that host i excretes into the environment during time step $t \to t + \delta t$. $O_{ij,t}$. This quantity is calculated based on the total egg output $E_{W,i,\text{transmission},t}$ of the worm population in host i at time t, the number of fertilised patent female worms $N_{W_f,i,t}$ in host i at time t (assuming all fertilised female worms within the same host contribute equally), and the rate $Co_{i,t}$ at which host i contributes to the environment at time t:

$$O_{ij,t} = \frac{E_{W,i,\text{transmission},t}}{N_{W_f,i,t}} \cdot Co_{i,t} \tag{28}$$

This package of genetic information and the associated number of eggs $O_{ij,t}$ is then made a candidate to become a particle of infective material in the environmental reservoir (more details in next section).

Dynamics of parasite genetics in the environmental reservoir

We simulate the dynamics of parasite genetics in the environmental reservoir while accounting for two important aspects. The first is that the genetic state of the parasite population and the environmental reservoir cannot be assumed to be in genetic equilibrium when under selective pressure from PC. The second is that the genetic make-up of eggs or larvae in the environmental reservoir follows a mixture distribution that consists of genetic information from worms in treated and untreated hosts as, typically, only a part of host population is targeted or reached with PC.

To avoid the computational cost of explicitly simulating the genetic make-up of every single egg or larva in the environment, we approximate the genetic mixture distribution with a large but finite and discrete set of infective particles. This particle population has a fixed size N_O (`n_aux_cloud`; default value of 10^4); the greater N_O, the more closely we approximate the true mixture distribution. Each particle o contains the following genetic information:

1. the mean of the genetic trait components $\mu_{\text{gen},o}$ of the particle's parent male-female worm pair that mated at some point, and 1. a vector with the two genotypes $N_{A,\text{f}}$ and $N_{A,\text{m}}$ from the same parent male-female worm pair.

Each particle has equal probability to infect a host and become a worm. However, the probability that a candidate particle produced by a female worm j in host i enters the particle population depends on the associated contribution $O_{ij,t}$ of female worm j to the total egg excretion of host i (see the section on within-host dynamics of parasite genetics). Because we approximate the genetic mixture distribution with a particle population of a finite and fixed size, any candidate particle that enters the particle population replaces a random existing particle. This is simulated as follows.

During each time step $t \rightarrow t + \delta t$, as long as the total amount of eggs excreted by all human hosts $E_{\text{excreted},t\rightarrow t+\delta t} > 0$, we update the mixture distribution by replacing a fraction $p_{\text{replace},t\rightarrow t+\delta t}$ of the particles with candidate particles produces by female worms. Here, $p_{\text{replace},t\rightarrow t+\delta t}$ depends on the total egg excretion $E_{\text{excreted},t\rightarrow t+\delta t}$ by human hosts and the amount of infective material in the environmental reservoir $Res_{t+\delta t}$ at the end of time step $t \rightarrow t + \delta t$:

$$p_{\text{replace},t\rightarrow t+\delta t} = \frac{E_{\text{excreted},t\rightarrow t+\delta t}}{Res_{t+\delta t}} = \frac{E_{\text{excreted},t\rightarrow t+\delta t}}{e^{-\delta t\cdot\psi} \cdot Res_t + E_{\text{excreted},t\rightarrow t+\delta t}} \tag{29}$$

We then randomly sample and remove $N_O \cdot p_{\text{replace},t\rightarrow t+\delta t}$ existing particles (rounded to the nearest integer) from the environmental reservoir (i.e., these particles are discarded). Next, we supplement the particle population back to size N_O with candidate particles from all female worms, which are sampled with replacement, weighted by the contribution O_{ij} of each female worm j in each host i.

Genetic drift

The processes for the dynamics of parasite genetics in the host and the environmental reservoir allow for natural genetic drift, even in absence of selective pressure. This also happens while warming up the simulation, meaning that at some time point of interest during the simulation (e.g., just prior to the first round of PC) the distribution of genotypes may be different from the specified initial allele frequency P_{snp} (`p_SNP`). Likewise, the population mean of the quantitative trait drifts in absence of selective pressure (the population mean is initialised at zero). This is exemplified in Figure A6.

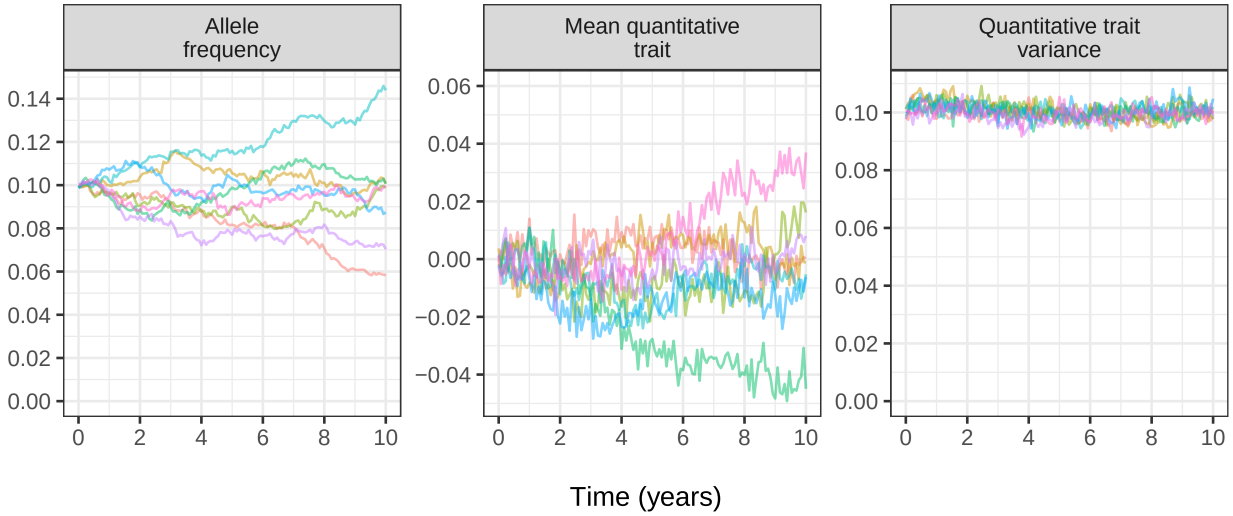

Figure A6: Example of genetic drift of allele frequencies (left panel, initial allele frequency $P_{\text{snp}} = 0.1$) and quantitative trait values (middle and right panels, $\sigma^2_{\text{trait}} = 0.1$, $h^2 = 0.3$, and $p_{\text{trait,t}} = 1.0$) for *Ascaris lumbricoides*. Each coloured line represent a stochastic simulation.

To have maximum control over the initial genetic state of the parasite population at the start of control interventions, optionally, the user can reset the genetic state of the parasite population (i.e., genotype frequencies and trait distribution) once during the simulation at a specified time point (**`reset_genetics_time`**).[14] This reset is performed assuming genetic equilibrium.

Alternatively, the genetic state of the parasite population (along with the general state of the human and parasite populations) can be initialised based on a stored simulation state from a previous simulation (**`state_init`**). In this case, no assumption about genetic equilibrium is applied, unless genetics are explicitly reset with the optional argument **`reset_genetics_time`**. For an example, see below.

Preventive chemotherapy

We define preventive chemotherapy (PC) or mass drug administration (MDA) in terms of:

1. the timing t of treatment rounds,
2. the age bounds of the target population,
3. the therapeutic coverage p_{cov} of the target population (i.e., the proportion of the target population that effectively receives and takes the drug), and
4. the level of systematic non-participation θ_{sys} over repeated treatment rounds.

The timing of treatment rounds is defined as a vector of time points (**`mda_time`**, defined in years since start of the simulation). The target population (**`mda_age`**), therapeutic coverage (**`mda_cov`**), and the level non-systematic non-participation to PC (**`syst_part`**) are assumed to be the same for all treatment rounds throughout the simulation.

[14] Specifying a reset time < 0 means that the genetic state of the population is not reset during the simulation. The default value of **`reset_genetics_time`** is -1.

Individual participation

The probability that an eligible individual (i.e., an individual with age in the range defined by **mda_age**) participating in a treatment round depends on the overall user-defined coverage p_{cov} of the target popultion (**mda_cov**, defined as a probability) and the level of systematic non-participation to PC θ_{sys} (**syst_part**, defined on a scale from zero to one). The number of participating individuals N_{part} is always rounded such that the simulated participating fraction is closest to the user-defined value.

Systematic non-participation is implemented similar to a stochastic participation process described elsewhere [13]. Here, the number of participating individuals at treatment time t is defined as the N_{part} individuals with the highest treatment rank $r_{i,t}$. For each individual i, treatment rank $r_{i,t}$ is defined as a function of the coverage p_{cov}, the level of systematic non-participation θ_{sys}, an individual's lifelong inclination to participate in treatment rounds co_i (drawn at birth), and a random value $z_{i,t}$ (drawns for each individual and treatment time point) that governs the random component of participation:

$$r_{i,t} = \mu_0 + co_i \cdot \sqrt{\sigma^2_{sys}} + z_{i,t}$$

$$\begin{aligned} \mu_0 &= -\text{CDF}^{-1}(p_{cov}) \cdot \sqrt{1 + \sigma^2_{sys}} \\ \sigma^2_{sys} &= \frac{\theta_{sys}}{1 - \theta_{sys}} \\ \\ co_i &\sim N(0,1) \\ z_{i,t} &\sim N(0,1) \end{aligned} \tag{30}$$

Here, μ_0 is defined as a z-score based on the inverse cumulative distribution function (CDF) of the standard normal distribution, scaled by a measure of the degree of systematic non-participation ($\sqrt{1 + \sigma^2_{sys}}$). Although μ_0 is superfluous in our current implementation (it is a constant that does not affect an individuals relative treatment rank), we keep it in the equation as it does play a role in the fully stochastic (i.e., binomial) implementation of this process where participation is determined as $r_{i,t} < 0$.

When $\theta_{sys} = 0$, individual participation is completely random as it is entirely governed by $z_{i,t}$ and p_{cov}. In contrast, as θ_{sys} approaches 1, individual (non-)participation becomes completely systematic as it is entirely governed by co_i. Values of θ_{sys} between zero and one therefore present a gradual range of mixed random and systematic non-participation.

Parasitological effects of drug treatment

Given that an individual participates in a treatment round, the probability $p_{\text{kill},j}$ that a worm j in that individual is killed depends on a worm's provisional phenotype according to the monogenic genotypes (**pheno_kill**) as well as the worm's quantitative trait (see also the section on genetics). Either one or both of these components may be relevant, depending on user-defined quantification of parasite genetics. Here, we describe the calculation of the parasitological effects of drug treatment encompassing both components, agnostic of whether one and/or both are turned off by the user. Note that we do not explicitly consider (host-level) variation in treatment effects due to pharmacokinetics or nutritional factors (apart perhaps from random (worm-level) noise in treatment effects in the polygenic resistance mechanics). Although such effects can be important in clinical and experimental studies, here we assume them to be minor or negligible compared to all of the other sources of individual heterogeneity in the model (exposure levels, worm loads, PC uptake, stochastic treatment effects).

In the section on the within-host dynamics of genetics, we described that a worm's provisional phenotype $p^*_{\text{kill},j}$ is assigned upon establishment of the worm in a host, depending on the number $N_{A,j}$ of resistance-conferring alleles that a worm has and the definition of which phenotype p_{kill} (**pheno_kill**) is associated

with each genotype (aa, aA, and AA). Then, given the quantitative trait $x_{\text{trait},j}$ of worm j, the probability $p_{\text{kill},j}$ that this worm is killed by drug treatment is:

$$
\begin{aligned}
p_{\text{kill},j} &= \text{logit}^{-1}(\text{logit}(p^{*}_{\text{kill},j}) - x_{\text{trait},j}) \\
\text{logit}(p) &= \ln\left(\frac{p}{1-p}\right) \\
\text{logit}^{-1}(q) &= \frac{1}{1+e^{-q}}
\end{aligned}
\tag{31}
$$

Drug efficacy monitoring

A user-defined time (**`retest_time`**) after each treatment round, the model will store expected egg counts (conditional on the user-defined faecal sample weight, without sampling of discrete counts) for all simulated individuals that were treated in the last round (if they are still alive and present in the simulation). This output can be analysed outside the simulation for, e.g., detectability of drug resistance.

The period between treatment and retesting must be a multiple of the time step δt in the simulation (otherwise retesting will simply not happen). Furthermore, two consecutive treatment rounds must not be scheduled closer together in time than **`retest_time`**, or the next treatment round will interfere with the results of retesting.

Survey output

At a user-defined frequency per simulated year (**`survey_freq`**, which must be an integer divisor of the number of simulation time steps **`steps`** per year), the model produces output based on a hypothetical survey among the entire human population. For the population as a whole, as well as user-defined age categories (**`age_output`**), the model summarises the state of the population in terms off:

1. the number of hosts;
2. the number worms (any, female, male, and prepatent);
3. the number of hosts with detectable eggs, based on a negative binomial draw for observed egg count(s) in each individual (details below);
4. the number of hosts with light, moderate, or heavy intensity infection based on the simulated number of eggs;
5. the expected and observed (i.e., sampled) arithmetic mean egg count;
6. the force of infection in terms of the average number of new worms per host per year;
7. the number of worms with 1 (heterozygous) and 2 (homozygous) resistance-associated alleles;
8. the arithmetic mean drug efficacy;
9. the average and variance of the genetic component of the quantitative trait;
10. the average and variance of the quantitative trait, including environmental and temporal variation.

To generate output based on egg counts, for each individual N_s repeated diagnostic tests (**`n_sample`**; default value of 1) are performed assuming that egg counts in a single faecal sample follow a negative binomial distribution with mean $E_{W,i,\text{survey}}$ and shape k_s (**`k_sample`**; default value of 0.32), where the mean is defined as:

$$
\begin{aligned}
E_{W,i,\text{survey}} &= c \cdot E_{W,i,\text{transmission}} \\
c &= c_{\text{input}} \cdot b_{\text{input}} \cdot w_s
\end{aligned}
\tag{32}
$$

Here, w_s is the amount of faeces used for per diagnostic test (**w_sample**; default value of 1/24).[15] See the section on within-host dynamics for how the transmission-related worm productivity $E_{W,i,\text{transmission}}$ is calculated.

After drawing egg counts from the negative binomial distribution, all egg counts are translated to eggs per gram faeces (epg) by dividing the total number of detected eggs per person by the total weight of samples taken ($N_s \cdot w_s$).[16] Last, every individual is categorized as having no, light, moderate, or heavy infection, based on user-defined cut-offs in terms of epg (**intens_cut**).

Simulation algorithm

The simulation algorithm is implemented in the **simresist()** function. Below we describe the exact order in which the different simulation processes take place.

1. **Parse parameter values**

   a. Basic checks.
   b. Transform rate and probability parameters to the appropriate time step size used in the simulation.
   c. Calculate conditional parameters that are used internally, e.g., $\sigma^2_{\text{gen}} = (\sigma^2_{\text{trait}} \cdot h^2)/(1 + h^2)$.

2. **Initialise the simulation**

   a. Use initial simulation state if supplied by user, or
   b. Generate a random human population (and warm up its demograhpy), a random worm population within human hosts (N.B. the worm population is already overdispersed but not warmed up at this point), and an environmental reservoir. Parasite genetics are initialised assuming that alleles for monogenic drug resistance are in Hardy-Weinberg equilibrium and the quantitative trait for polygenic drug resistance in equilibrium at a mean value of zero with user-defined trait variance.

3. **Initialise simulation output containers**

   a. A temporary file on disk to append simulation output to.
   b. A data.table object to hold individual-level expected egg counts from before and after each treatment round.

4. **For each time step of the simulation, loop through the following:**

   a. **Check if any worms are left**; if none and **break_if_elim =** TRUE, break out of the loop.
   b. **Reset worm genetics** (if it is the time for it; this can only happen once during a simulation).
   c. **Generate a survey for simulation summary output** (if it is the time for it).
   d. **Preventive chemotherapy / mass drug administration**
      - Re-draw the random temporal component of the quantitative trait for all worms.
      - Check whether its time to retest egg counts in individuals who were treated in a previous treatment round; if so:
        1. Update worm insemination status and egg output.
        2. Retest egg counts.
      - Check whether its time to implement a treatment round; if so:
        1. Identify individuals to be treated.

[15]Note that the weight w_s (**w_sample**) of faecal samples should be specified in the same weight unit (default: grams) as the weight component of the unit for egg production parameters a_{input} and b_{input} (**egg_prod_a**, **egg_prod_b**; default unit: eggs per *gram* faeces).

[16]Predicted egg counts are expressed in terms of eggs per weight unit faeces, where the weight unit is the same as the one used to specify w_s, a_{input}, and b_{input} (**w_sample**, **egg_prod_a**, **egg_prod_b**) (default weight unit: grams).

2. Update worm insemination status and egg output.
3. Test egg counts of individuals that are to be treated.
4. Treat individuals.

e. **Transmission**

- Age the worm population by δt and mark worms that have reached the end of their lifespan.
- Update insemination status and egg output of living patent female worms.
- Calculate the amount of infective material excreted by hosts and the amount in the environmental reservoir that will survive time step δt.
- Update the amount of infective material in the reservoir to time $t + \delta t$.
- Update the genetic information of infective material in the reservoir to time $t + \delta t$.
- Calculate the force of infection acting on the human population and draw a value for the number of new worms introduced into the human population.
- Generate new worms and their genetic make-up based on the genetic state of the environmental reservoir, and assign new worms to hosts.
- Add new worms to the worm population while removing worms who have died or whose hosts have died.

a. **Update human demography**

- Age the human population by δt.
- Update human age-dependent fertility rates.
- Identify and mark humans who have reached the end of their lifespan, as well as any worms living in them.
- Check whether the living human population size exceeds the maximum specified size; if so, mark a random fraction of living humans for removal.
- Generate human births, and add them to the human population while removing dead humans and individuals marked for removal.
- Update age and time-dependent exposure and contribution rates of humans.

5. **Read the simulation output from the temporary file on disk into memory (and add one last final survey)**.

6. **Collate and return output to the user** (see `?simresist` and `?gen_output` for details about what is produced).

Examples

This section provides some example code for using `simresist` and visualising simulation results.

Running a simulation

To simulate the impact of PC, the user should first warm up the simulation sufficiently for the adult worm population to stabilise. As 50 years is typically long enough for this, we specify a simulation time (**`runtime`**) of 55 years and start annual PC in the 50th simulation year (**`mda_time`**). Note that before simulating the specified 55 years, `simresist` will warm up human demography for a default duration of 250 year(s) (use the argument **`human_warmup_duration`** to define another duration). We assume that PC is targeted at school age children (**`mda_age`**) and that 95% of children are effectively treated (**`mda_cov`**). A single treatment is assumed to kill 95% of adult worms (**`pheno_kill`**). Note that three values have to be specified: one for each potential genotype in case of monogenic resistance. We further assume that no drug resistance will evolve by setting the initial allele frequency for monogenic resistance (**`p_SNP`**) to zero and setting the quantitative trait for polygenic resistance to have zero variation (**`trait_var`**) and zero heritability (**`trait_h2`**). We assume that this setting is endemic for *Necator americanus* (**`species`**) and set the transmission rate (**`zeta`**) such that we get a situation with just under 50% pre-control prevalence of infection (egg-positivity). We simulated

trends in weekly time steps (**steps**), i.e., 52 time steps per simulated year and we make the model generate weekly output (**survey_freq**). For now, we suppress the printing of simulation progress to the console (**verbose**):

```
set.seed(123456)
output_PC_impact <- simresist(runtime = 55,
                              steps = 52,
                              survey_freq = 52,
                              species = "hook",
                              zeta = 175,
                              mda_time = 50:54,
                              mda_cov = 0.95,
                              mda_age = c(5, 15),
                              pheno_kill = rep(0.95, 3),
                              p_SNP = 0,
                              trait_var = 0,
                              trait_h2 = 0,
                              verbose = FALSE)
```

The resulting list object `output_PC_impact` includes the element `monitor_age`, which contains age-specific model output. We can now use `ggplot()` to plot, for instance, trends in infection levels in school age children and in adults, cutting off most of the warm-up period:

```
ggplot(mapping = aes(x = time - 50, y = n_host_eggpos / n_host * 100)) +
  geom_vline(xintercept = output_PC_impact$param$mda_time - 50,
             lty = 2, col = "darkgrey", alpha = .5) +
  geom_line(data = output_PC_impact$monitor_age[age_cat == 15 & time >= 49],
            mapping = aes(col = "Adults")) +
  geom_line(data = output_PC_impact$monitor_age[age_cat == 5 & time >= 49],
            mapping = aes(col = "School age children")) +
  scale_x_continuous(name = "\nTime since start of PC (years)",
                     breaks = -1:5, minor_breaks = NULL) +
  scale_y_continuous(name = "Prevalence of eggs (%)\n") +
  scale_colour_discrete(name = NULL) +
  expand_limits(y = 0) +
  theme_bw()
```

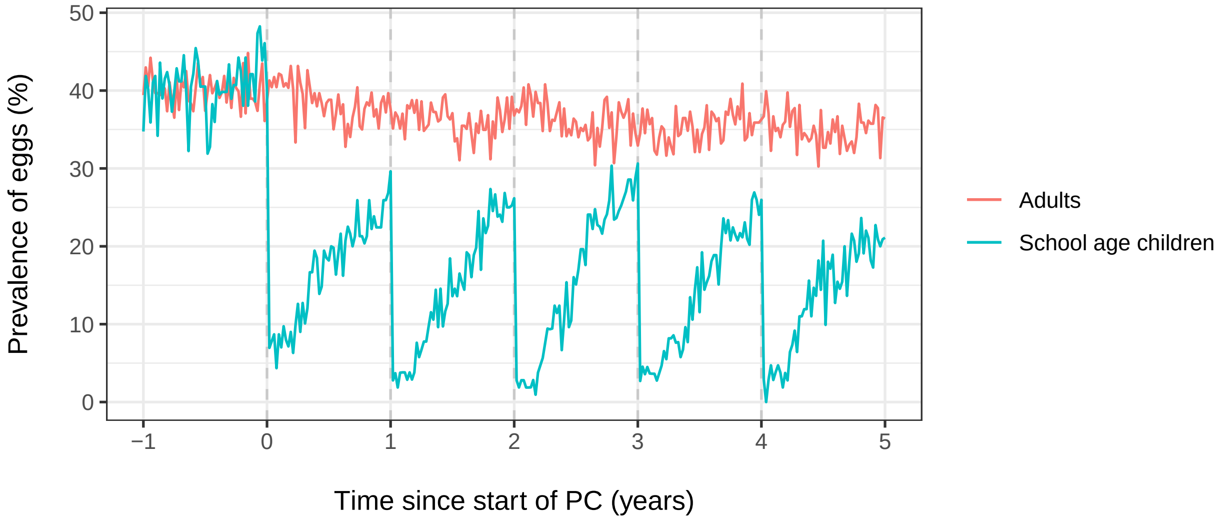

Figure A7: Model-predicted trend in prevalence of eggs in faeces during annual PC (vertical dashed lines) targeted at school age children (age 5-15), implemented at 95% coverage. No evolution of drug resistance is assumed.

The resulting Figure A7 shows how after each round of PC (vertical dashed lines), infection levels drop in school age children, but then bounce back up until the next treatment. Infection levels in adults are not directly affected as they are not treated, although they decline somewhat due to the effect of treating children on the overall intensity of transmission in the community.

Evolution of drug resistance

Now, we will consider a scenario with homogenic inheritance of drug resistance with co-dominant penetrance; we assume that worms with the "wildtype" genotype aa are still fully susceptible to treatment (95% probability of being killed), but worms with genotypes aA and AA are resistant (40% and 10% probability of being killed by treatment, respectively). We specify this by defining the argument **`pheno_kill`** as `c(0.95, 0.40, 0.10)`, where the three positions in this vector represent the three genotypes aa, aA, and AA. We further set the initial allele frequency for monogenic resistance (**`p_SNP`**) to 5%. To enforce that the SNP frequencies in the simulation are as close as possible to the frequencies that we specified, we overwrite any genetic drift during the warm-up period by resetting all genetics one year before the first PC round (**`reset_genetics_time=`** `49`). We further assume that PC is targeted at school age children, implemented annually at 95% coverage.

```
set.seed(123456)
output_PC_resist <- simresist(runtime = 60,
                              steps = 52,
                              survey_freq = 52,
                              species = "hook",
                              zeta = 175,
                              mda_time = 50:59,
                              mda_cov = 0.95,
                              mda_age = c(5, 15),
                              pheno_kill = c(0.95, .40, 0.10),
                              p_SNP = 0.05,
```

```
                      trait_var = 0,
                      trait_h2 = 0,
                      reset_genetics_time = 49,
                      verbose = FALSE)
```

We again plot trends in infection levels in school age children and in adults, cutting off most of the warm-up period:

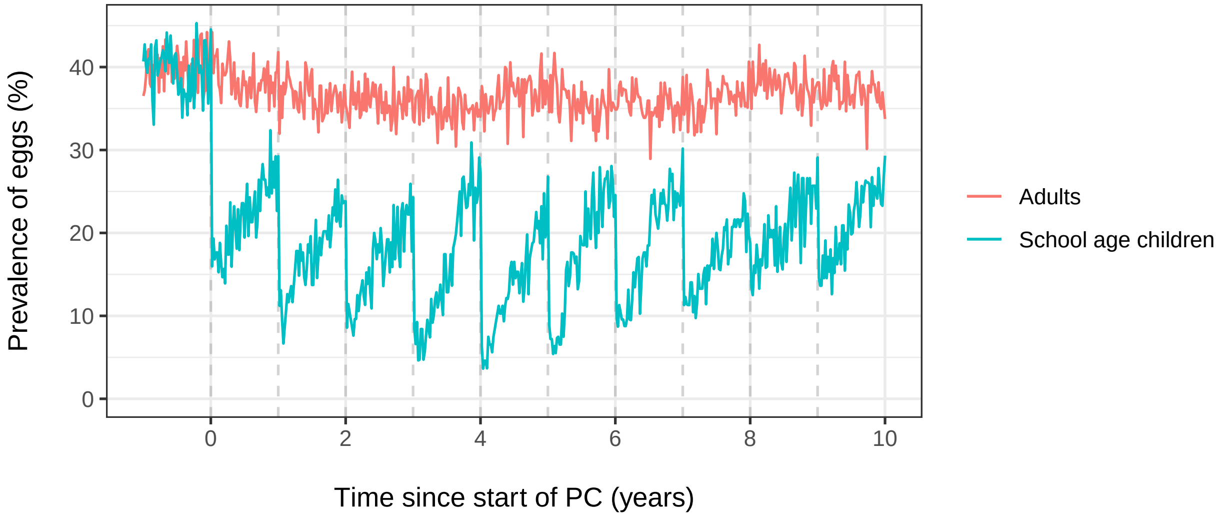

Figure A8: Model-predicted trend in prevalence of eggs in faeces during annual PC (vertical dashed lines) targeted at school age children (age 5-15), implemented at 95% coverage.

As we see in Figure A8, PC seems to impact infection levels as expected, but from year 4 onwards, infection levels seem to gradually drop less and less over time. In addition, genotype frequencies are changing noticably:

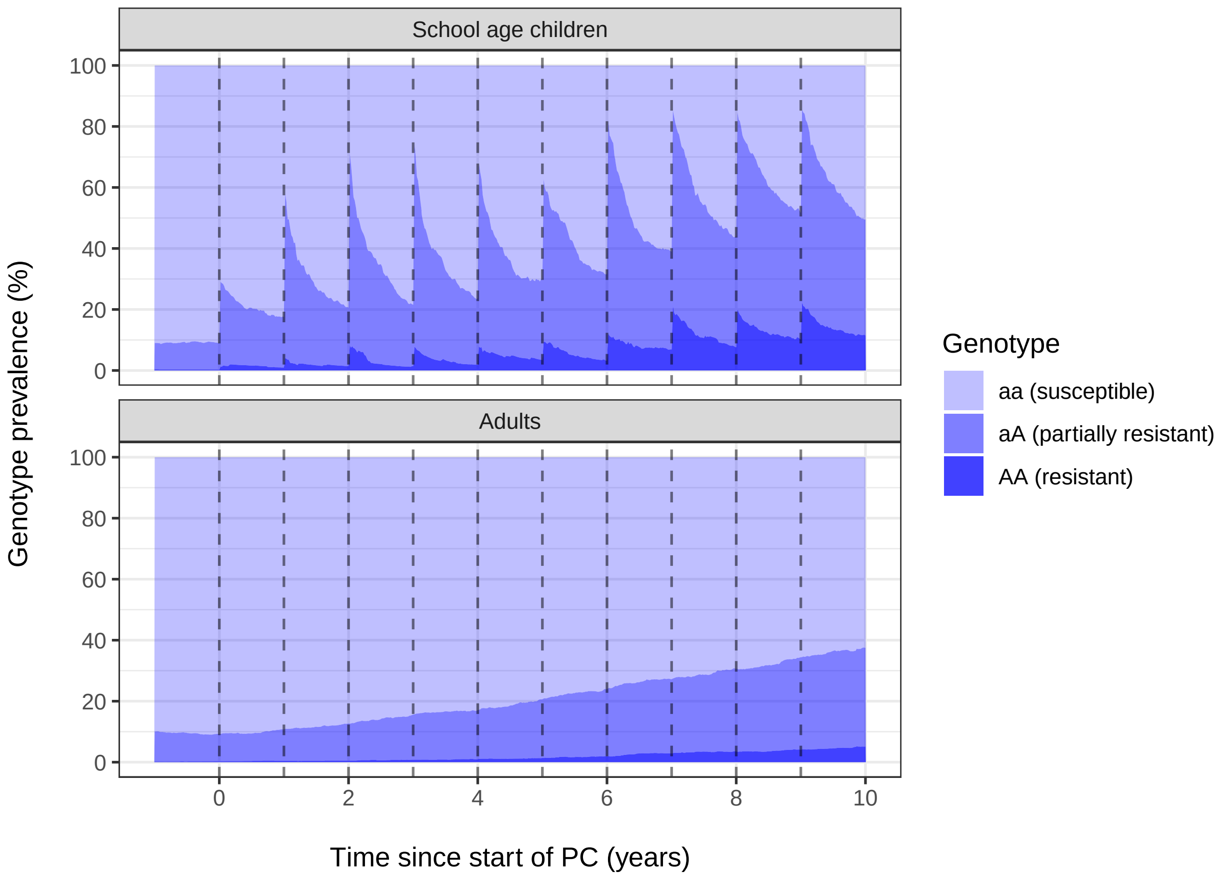

Figure A9: Model-predicted trend in allele frequency during annual PC (vertical dashed lines) targeted at the population of age 2 and above, implemented at 70% coverage.

In Figure A9, we see how after every PC round, the prevalences of genotype aA and AA spike in children. However, between treatment rounds, the genotype prevalences move back toward Hardy-Weinberg equilibrium due to transmission of infection from adults to school age children. This equilibrium does however shift over time, as can be seen in the slowly changing prevalence of genotype aA in adults due to transmission of infection from school age children to adults.

Initialising a simulation based on a previously saved population state

If we want to simulate a change in PC strategy, we need to start a new simulation, using the final state of a previous simulation as the starting point for the new simulation, using the **state_init** argument of the `simresist()` function. This is necessary as `simresist` assumes that the PC target population is fixed for the entirety of a simulation. For instance, let us simulate the impact of changing from targeting school age children (95% coverage) to the entire community (70% coverage):

```
set.seed(123456)
output_continued <- simresist(state_init = output_PC_resist$state,
                              runtime = 10,
                              steps = 52,
```

```
survey_freq = 52,
species = "hook",
zeta = 175,
mda_time = 0:9,
mda_cov = 0.7,
mda_age = c(2, Inf),
pheno_kill = c(0.95, .40, 0.10),
verbose = FALSE)
```

We again plot trends in infection levels in school age children and in adults, cutting off most of the warm-up period. Note that in the previous simulation, we scheduled the treatment round at the start of year 59, and finished the simulation by year 60. Therefore, here, we specify that the first community-wide PC round will take place in year 0 of the new simulation (`mda_time`), which is effectively 60 years after the start of the previous simulation.

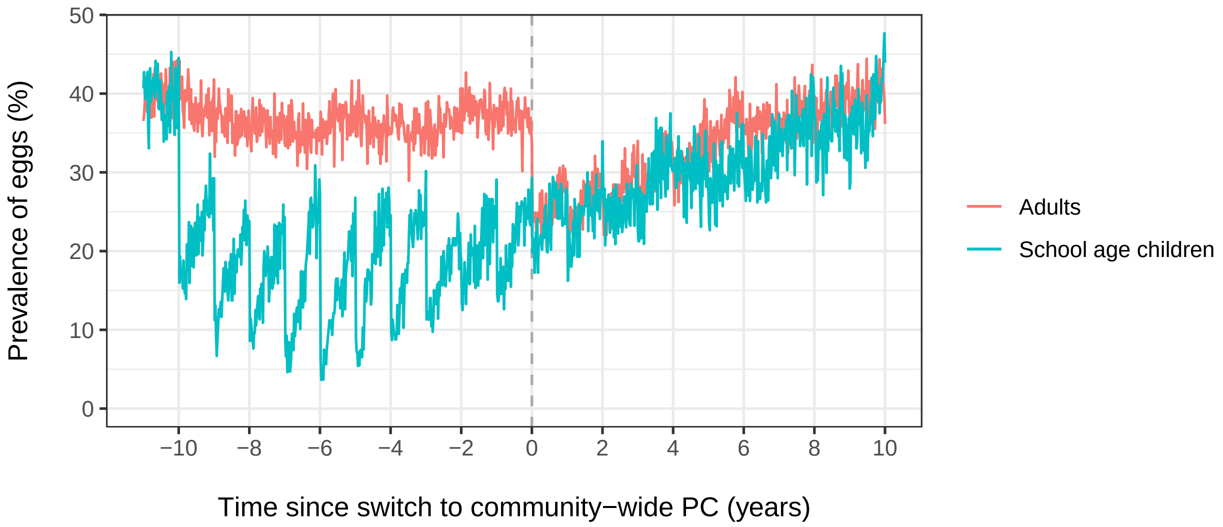

Figure A10: Model-predicted trend in prevalence of eggs in faeces during annual PC after switching (vertical dashed line) from targeting of school age children (95%) to targeting of the population of age 2 and above (70% coverage).

As we see in Figure A10, after switching to annual PC targeting both children and adults (vertical dashed line), the impact of PC quickly diminishes over time, resulting in a steady increase of infection levels towards pre-control levels. This dynamic is also reflected in the change of genotype frequencies. In Figure A11, we see how broadening the target group of PC (vertical dashed line) increases the selective pressure on the worm population, accelerating the evolution of widespread drug resistance.

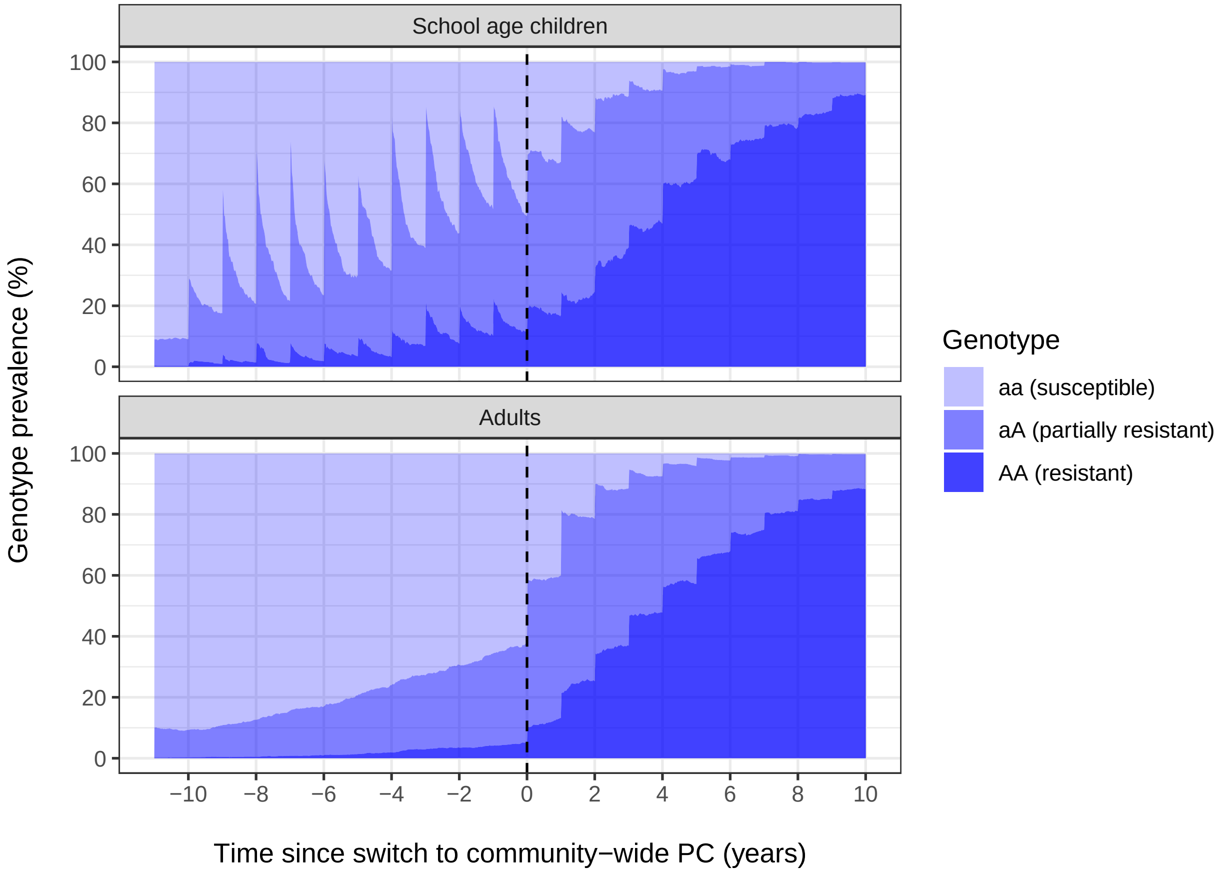

Figure A11: Model-predicted trend in allele frequency during annual PC after switching (vertical dashed line) from targeting of school age children (95%) to targeting of the population of age 2 and above (70% coverage).

This programming technique can also be used to investigate the impact of various alternative PC strategies in a particular population. To do this, first generate a "baseline" population state and then use it to initialise various simulations for alternative scenarios (as extensively used in the study that is described in the paper that this appendix accompanies).

Comparison to WORMSIM

Here, we provide comparison of model predictions by WORMSIM (v4.5.2) and `simresist` for a range of epidemiological and programmatic scenarios, demonstrating that `simresist` (with drug resistance turned off) closely reproduces the original WORMSIM model behaviour (Figure A12).

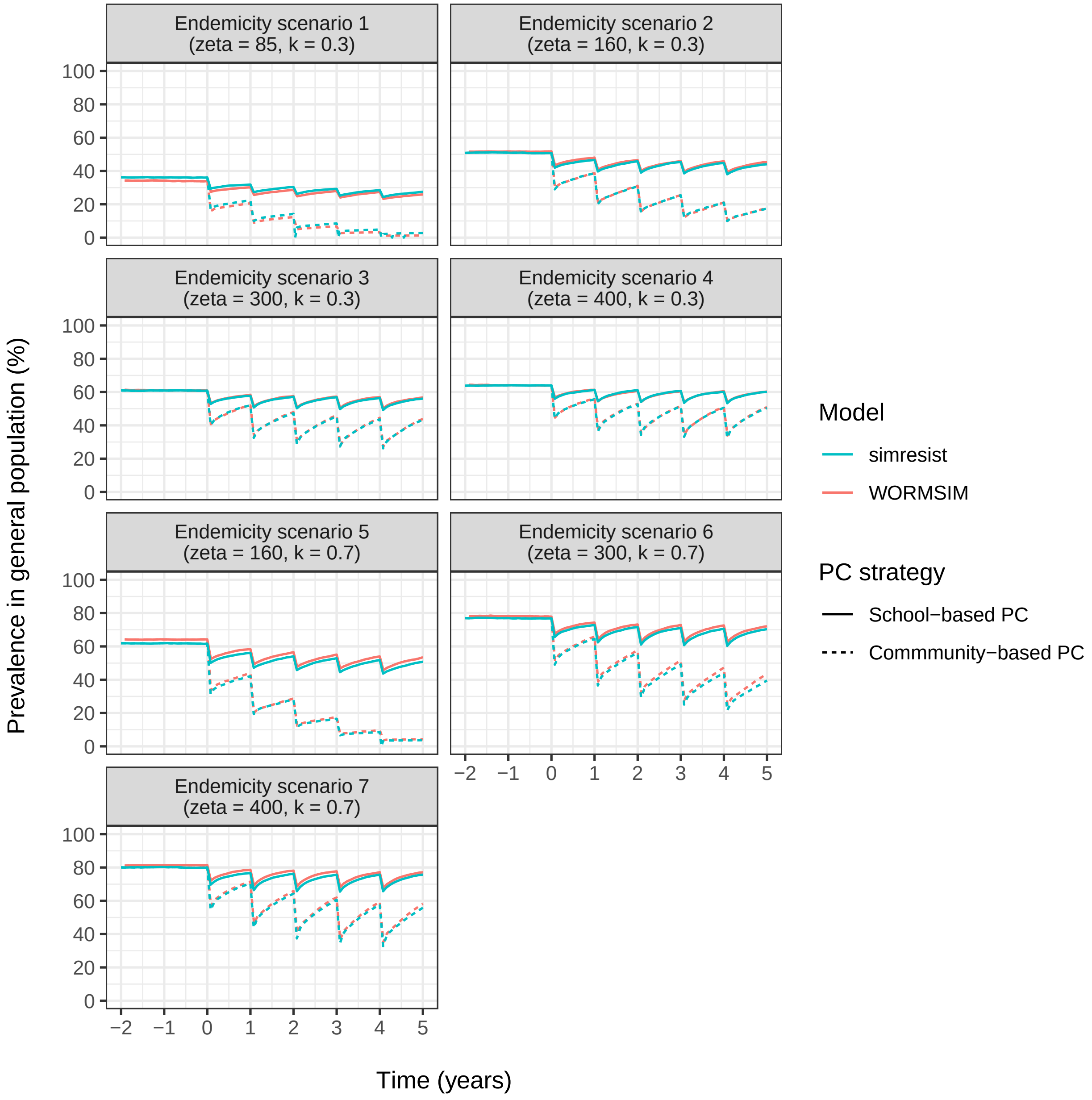

Figure A12: Comparison of trends in prevalence of infection with at least one adult female worm, as predicted for hookworms by simresist and WORMSIM, in absence of drug resistance. School-based PC was assumed to reach 95% of children (age 5-15) and community-based PC was assumed to reach 70% of all individuals of age 2 and above. PC participations is assumed to be random in both cases. Lines represent the average of 50 repeated simulations.

References

1. Coffeng LE, Bakker R, Montresor A, de Vlas SJ. Feasibility of controlling hookworm infection through preventive chemotherapy: a simulation study using the individual-based WORMSIM modelling framework. Parasit Vectors. 2015;8: 541. doi:10.1186/s13071-015-1151-4
2. Coffeng LE, Truscott JE, Farrell SH, Turner HC, Sarkar R, Kang G, et al. Comparison and validation of two mathematical models for the impact of mass drug administration on Ascaris lumbricoides and hookworm infection. Epidemics. 2017;18: 38–47. doi:10.1016/j.epidem.2017.02.001
3. Coffeng LE, Vaz Nery S, Gray DJ, Bakker R, de Vlas SJ, Clements ACA. Predicted short and long-term impact of deworming and water, hygiene, and sanitation on transmission of soil-transmitted helminths. Walker M, editor. PLoS Negl Trop Dis. 2018;12: e0006758. doi:10.1371/journal.pntd.0006758
4. Farrell SH, Coffeng LE, Truscott JE, Werkman M, Toor J, Vlas SJ de, et al. Investigating the Effectiveness of Current and Modified World Health Organization Guidelines for the Control of Soil-Transmitted Helminth Infections. Clin Infect Dis. 2018;66: S253–S259. doi:10.1093/cid/ciy002
5. Giardina F, Coffeng LE, Farrell SH, Vegvari C, Werkman M, Truscott JE, et al. Sampling strategies for monitoring and evaluation of morbidity targets for soil-transmitted helminths. Lustigman S, editor. PLoS Negl Trop Dis. 2019;13: e0007514. doi:10.1371/journal.pntd.0007514
6. Vegvari C, Giardina F, Bajaj S, Malizia V, Hardwick RJ, Truscott JE, et al. Deworming women of reproductive age during adolescence and pregnancy: what is the impact on morbidity from soil-transmitted helminths infection? Parasit Vectors. 2021;14: 220. doi:10.1186/s13071-021-04620-w
7. Vegvari C, Giardina F, Malizia V, Vlas SJ de, Coffeng LE, Anderson RM. Impact of key assumptions about the population biology of soil-transmitted helminths on the sustainable control of morbidity. Clin Infect Dis. 2021;[in press]. doi:10.1093/cid/ciab195
8. Malizia V, Giardina F, Vegvari C, Bajaj S, McRae-McKee K, Anderson RM, et al. Modelling the impact of COVID-19-related control programme interruptions on progress towards the WHO 2030 target for soil-transmitted helminths. Trans R Soc Trop Med Hyg. 2021;115: 253–260. doi:10.1093/trstmh/traa156
9. United Nations Department of Economic and Social Affairs Population Division. World Population Prospects: The 2012 Revision, Volume I: Comprehensive Tables. 2013. Available: http://esa.un.org/wpp/index.htm
10. Anderson RM, Truscott JE, Pullan RL, Brooker SJ, Hollingsworth TD. How effective is school-based deworming for the community-wide control of soil-transmitted helminths? PLoS Negl Trop Dis. 2013;7: e2027. doi:10.1371/journal.pntd.0002027
11. Hoagland KE, Schad GA. Necator americanus and Ancylostoma duodenale: Life history parameters and epidemiological implications of two sympatric hookworms of humans. Exp Parasitol. 1978;44: 36–49. doi:10.1016/0014-4894(78)90078-4
12. Elkins DB, Haswell-Elkins M, Anderson RM. The epidemiology and control of intestinal helminths in the Pulicat Lake region of Southern India. I. Study design and pre- and post-treatment observations on Ascaris lumbricoides infection. Trans R Soc Trop Med Hyg. 1986;80: 774–92. Available: http://www.ncbi.nlm.nih.gov/pubmed/3603617
13. Irvine MA, Reimer LJ, Njenga SM, Gunawardena S, Kelly-Hope L, Bockarie M, et al. Modelling strategies to break transmission of lymphatic filariasis - aggregation, adherence and vector competence greatly alter elimination. Parasit Vectors. 2015;8: 547. doi:10.1186/s13071-015-1152-3

R session info

This document and all its contents were created in an R session with the following characteristics:

```
sessionInfo()
#> R version 4.3.1 (2023-06-16)
#> Platform: aarch64-apple-darwin20 (64-bit)
#> Running under: macOS Sonoma 14.1.2
#>
#> Matrix products: default
#> BLAS:   /Library/Frameworks/R.framework/Versions/4.3-arm64/Resources/lib/libRblas.0.dylib
#> LAPACK: /Library/Frameworks/R.framework/Versions/4.3-arm64/Resources/lib/libRlapack.dylib;  LAPACK v
#>
#> locale:
#> [1] en_US.UTF-8/en_US.UTF-8/en_US.UTF-8/C/en_US.UTF-8/en_US.UTF-8
#>
#> time zone: Europe/Amsterdam
#> tzcode source: internal
#>
#> attached base packages:
#> [1] parallel  stats     graphics  grDevices utils     datasets  methods
#> [8] base
#>
#> other attached packages:
#> [1] simresist_1.1.1    data.table_1.14.10 ggplot2_3.4.4      kableExtra_1.3.4
#> [5] doParallel_1.0.17  iterators_1.0.14   foreach_1.5.2
#>
#> loaded via a namespace (and not attached):
#>  [1] gtable_0.3.4      highr_0.10        dplyr_1.1.4       compiler_4.3.1
#>  [5] tidyselect_1.2.0  webshot_0.5.5     xml2_1.3.6        stringr_1.5.1
#>  [9] systemfonts_1.0.5 scales_1.3.0      boot_1.3-28.1     yaml_2.3.8
#> [13] fastmap_1.1.1     R6_2.5.1          labeling_0.4.3    generics_0.1.3
#> [17] knitr_1.45        tibble_3.2.1      munsell_0.5.0     svglite_2.1.1
#> [21] pillar_1.9.0      rlang_1.1.2       utf8_1.2.4        stringi_1.8.3
#> [25] xfun_0.41         viridisLite_0.4.2 cli_3.6.2         withr_2.5.2
#> [29] magrittr_2.0.3    digest_0.6.33     rvest_1.0.3       grid_4.3.1
#> [33] rstudioapi_0.15.0 lifecycle_1.0.4   vctrs_0.6.5       evaluate_0.23
#> [37] glue_1.6.2        farver_2.1.1      codetools_0.2-19  fansi_1.0.6
#> [41] colorspace_2.1-0  rmarkdown_2.25    httr_1.4.7        tools_4.3.1
#> [45] pkgconfig_2.0.3   htmltools_0.5.7
```

Supplementary Information B: Baseline population states

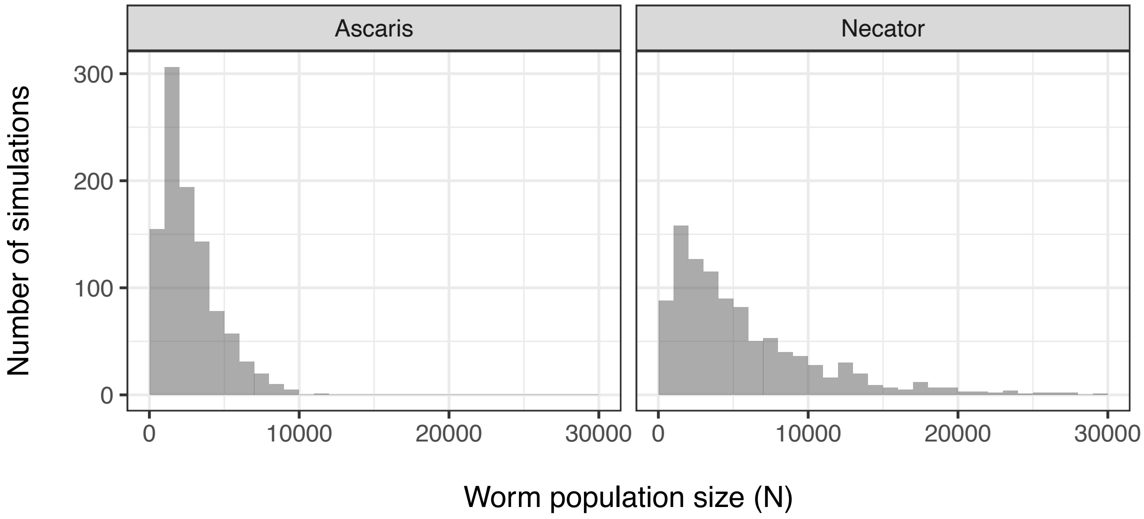

Figure B1. Simulated baseline worm population size (male and female worms). Results are shown for 1,000 baseline states in which at least one adult worm was present in the entire human population after the 300-year warm-up period. Note that population sizes are lower for *Ascaris lumbricoides* than for *Necator americanus*, primarily because the average adult lifespan of *A. lumbricoides* is 1 year (*N. americanus:* 3 years) and because exposure to *A. lumbricoides* transmission (via geophagia) is lower in adults than for *N. americanus* (transmission via skin exposure).

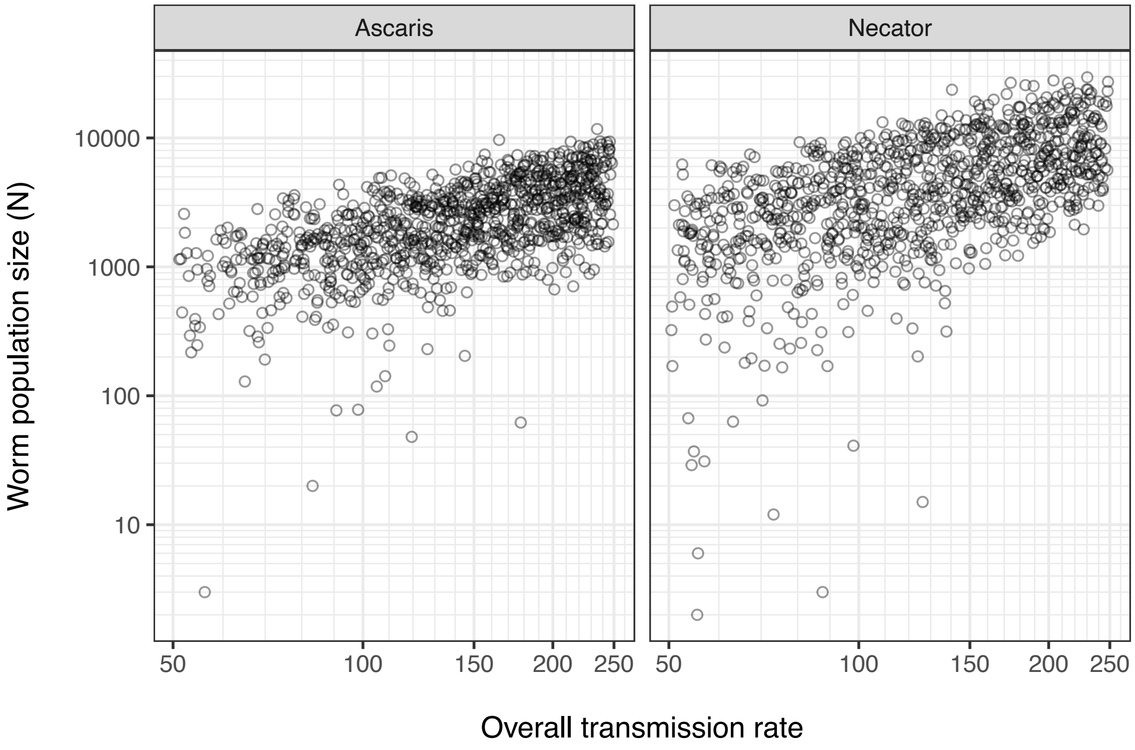

Figure B2. Simulated baseline worm population size (male and female worms) by transmission rate. Results are shown for 1,000 baseline states in which at least one adult worm was present in the entire human population after the 300-year warm-up period. Values on both axes are plotted on logarithmic scales.

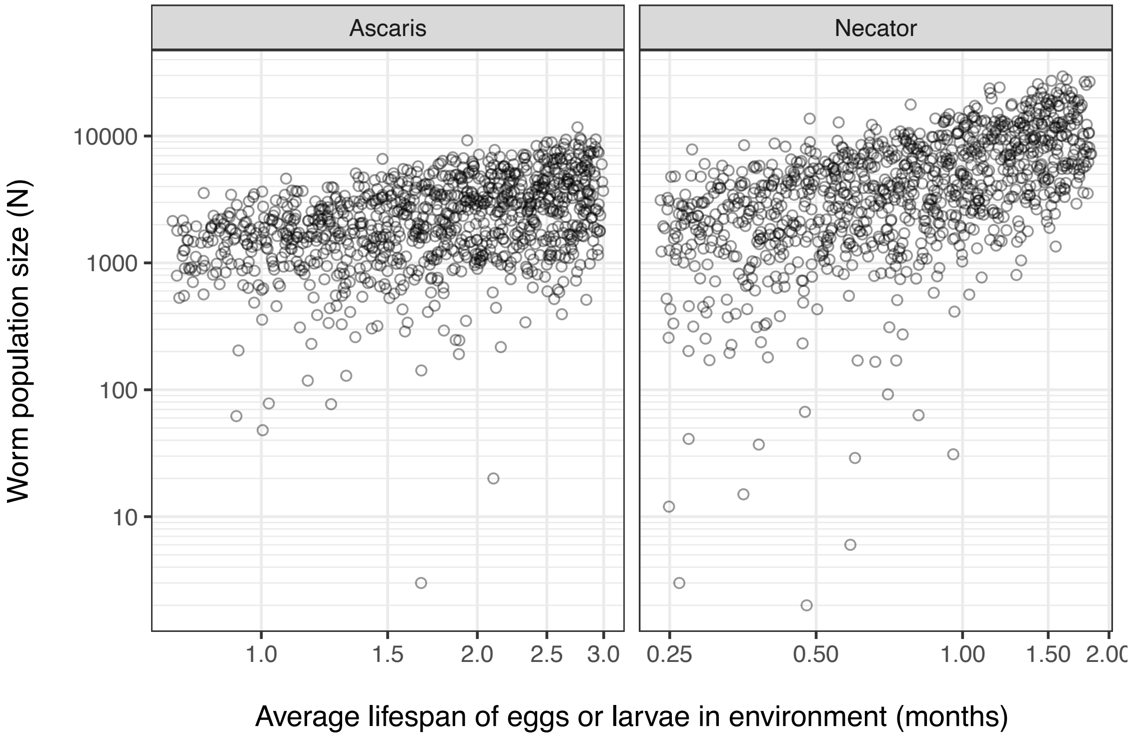

Figure B3. Simulated baseline worm population size (males and females) by average lifespan of eggs or larvae in the environment. Results are shown for 1,000 baseline states in which at least one adult worm was present in the entire human population after the 300-year warm-up period. Values on both axes are plotted on logarithmic scales.

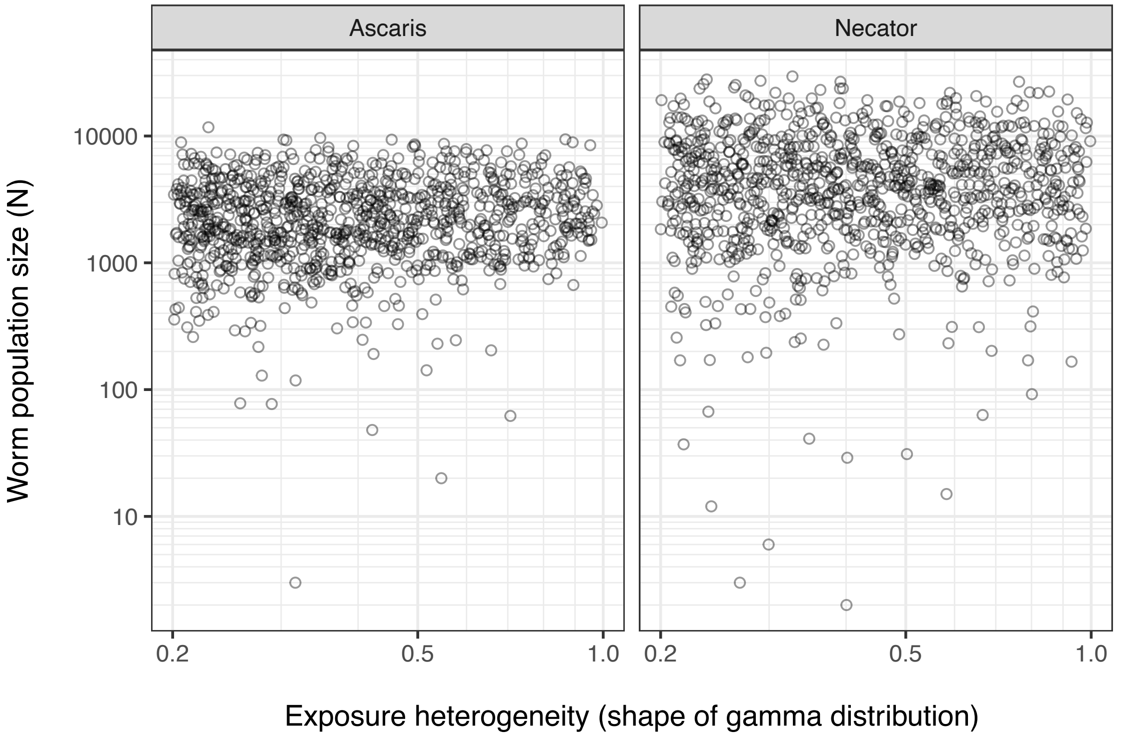

Figure B4. Simulated baseline worm population size (male and female worms) by level of exposure heterogeneity (degree of aggregation of worms in hosts). Results are shown for 1,000 baseline states in which at least one adult worm was present in the entire human population after the 300-year warm-up period. Note that higher values of the shape parameter indicate lower exposure heterogeneity. Values on both axes are plotted on logarithmic scales.

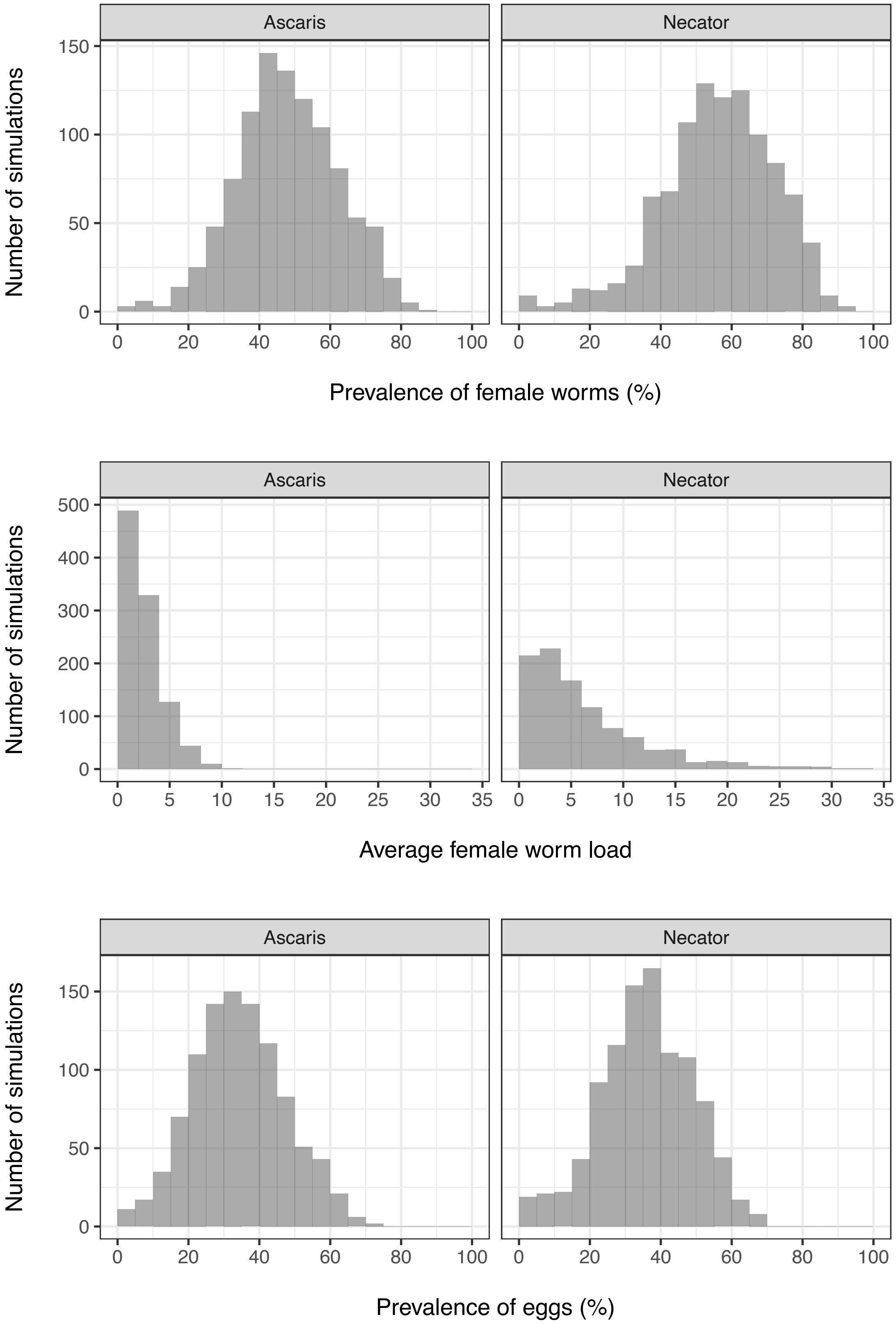

Figure B5. Simulated baseline infection levels in the general human population. Results are shown for 1,000 baseline states in which at least one adult worm was present in the entire human population after the 300-year warm-up period.

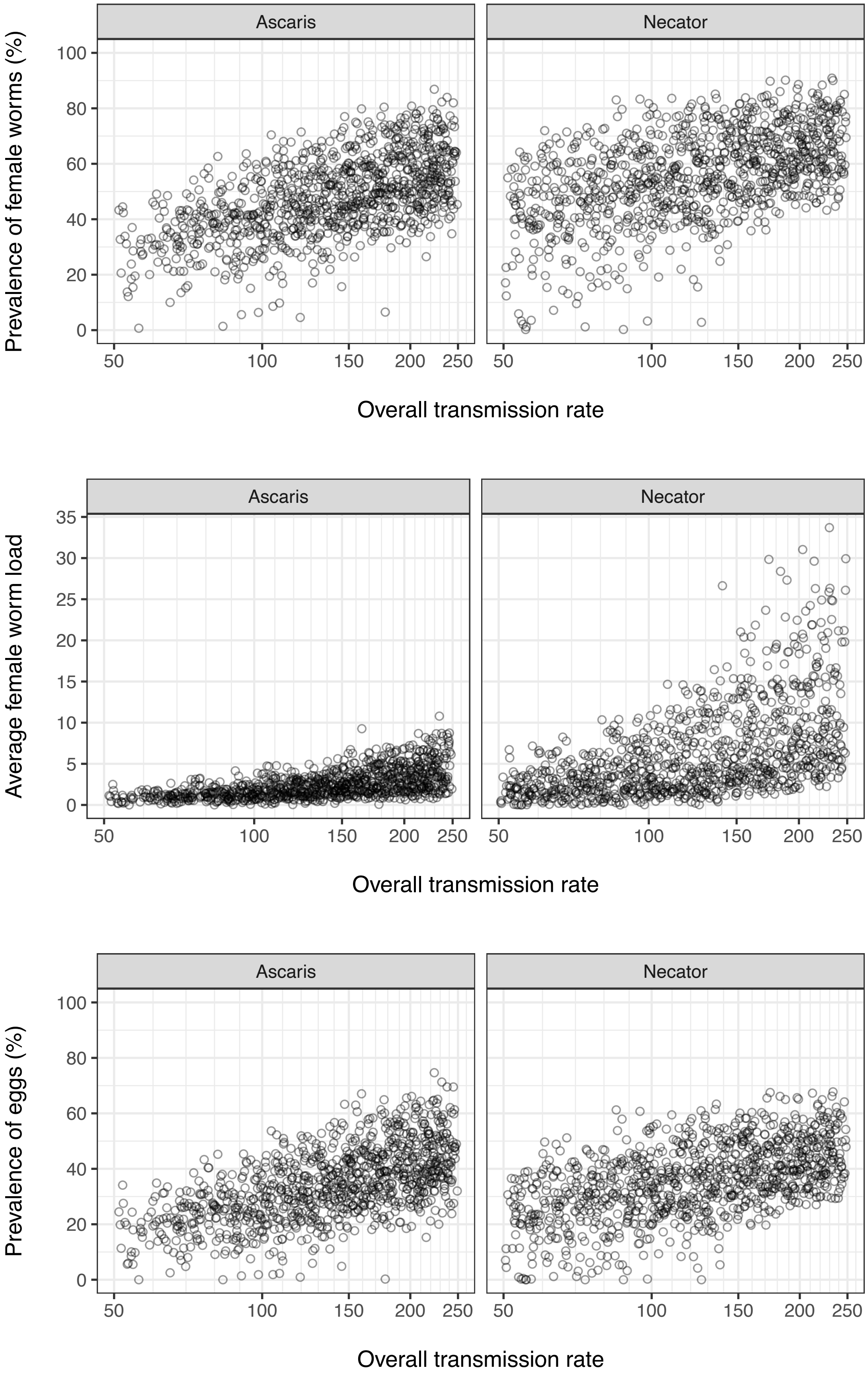

Figure B6. Association between transmission rate and baseline infection levels in the general human population. Results are shown for 1,000 baseline states in which at least one adult worm was present in the entire human population after the 300-year warm-up period. Values on the horizontal axis are plotted on a logarithmic scale.

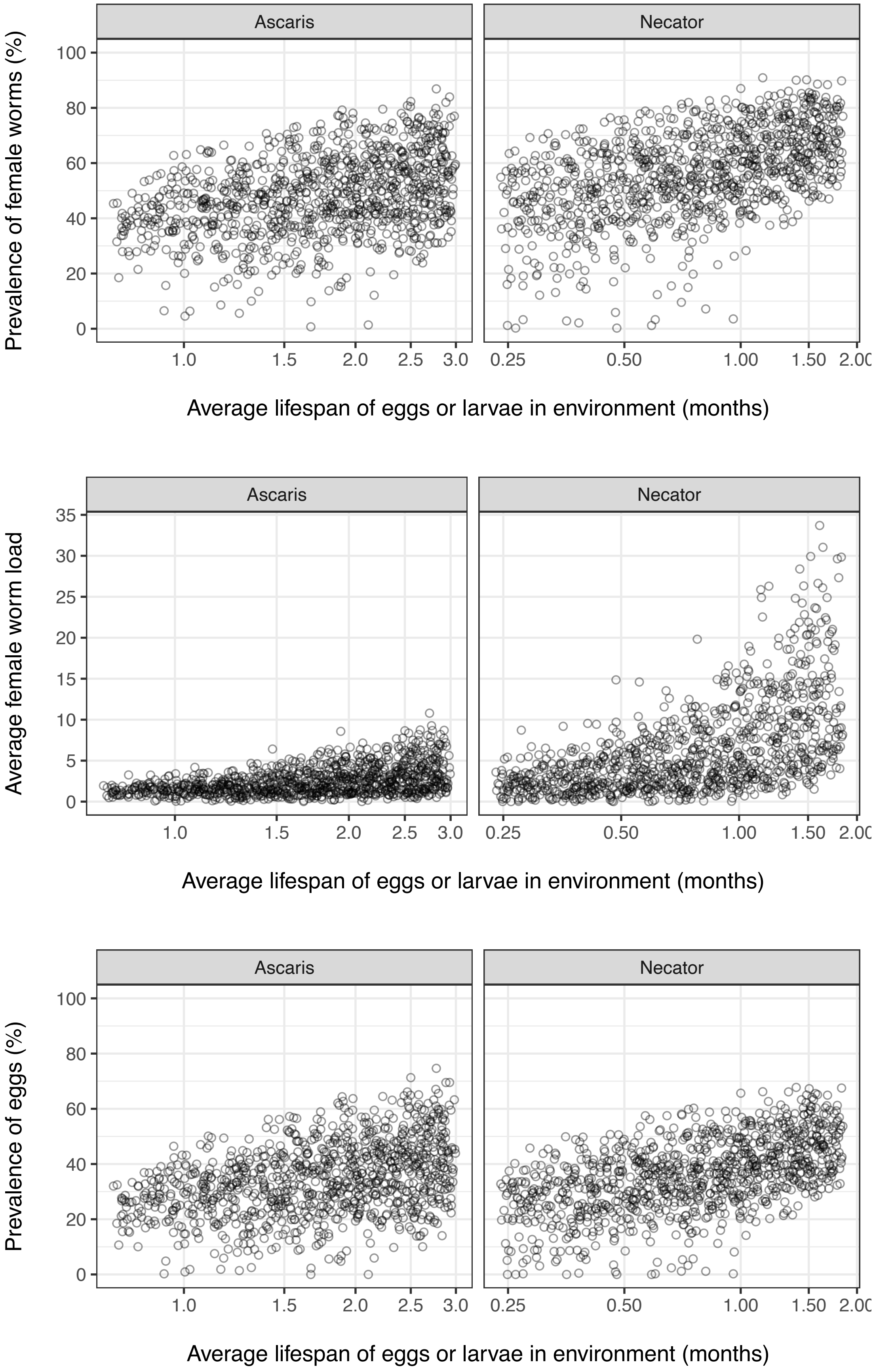

Figure B7. Association between average lifespan of eggs or larvae in the environment and baseline infection levels in the general human population. Results are shown for 1,000 baseline states in which at least one adult worm was present in the entire human population after the 300-year warm-up period. Values on the horizontal axis are plotted on a logarithmic scale.

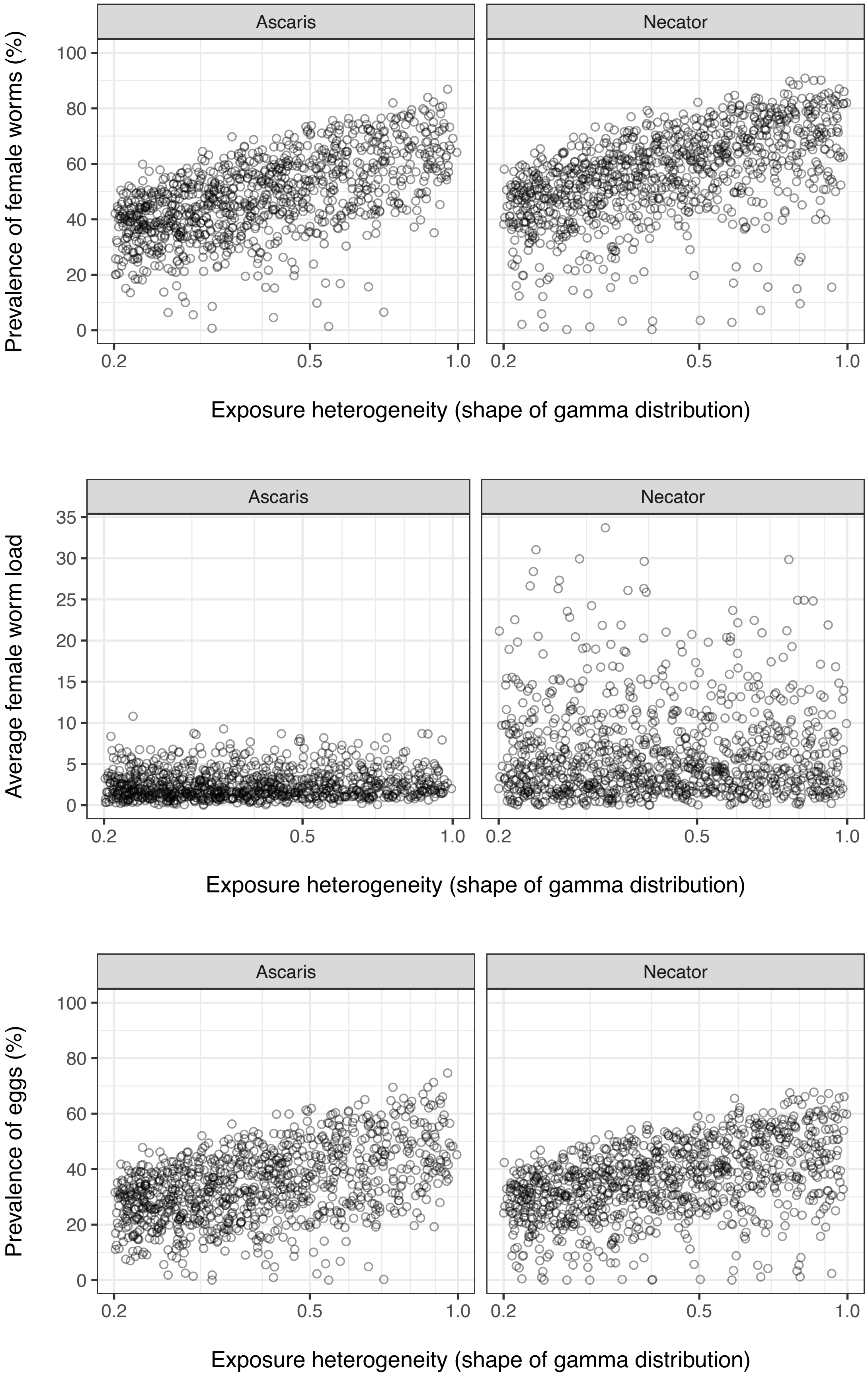

Figure B8. Association between exposure heterogeneity and baseline infection levels in the general human population. Results are shown for 1,000 baseline states in which at least one adult worm was present after the 300-year warm-up period. Note that higher values of the shape parameter indicate lower exposure heterogeneity. Values on the horizontal axis are plotted on a logarithmic scale.

Supplementary Information C: Predicted population dynamics over time

Figure C1. Model-predicted prevalence of female *Ascaris lumbricoides* worms in the general population over time. Solid red lines indicate the average of the simulations that did not reach elimination; the red dashed lines indicate the proportion of simulations that resulted in elimination over time (i.e., zero female worms left; also indicated for year 20 with P_{elim} in the corner of each panel). Thin lines represent single simulations, where the line colour indicates whether elimination was achieved (grey) or not (black).

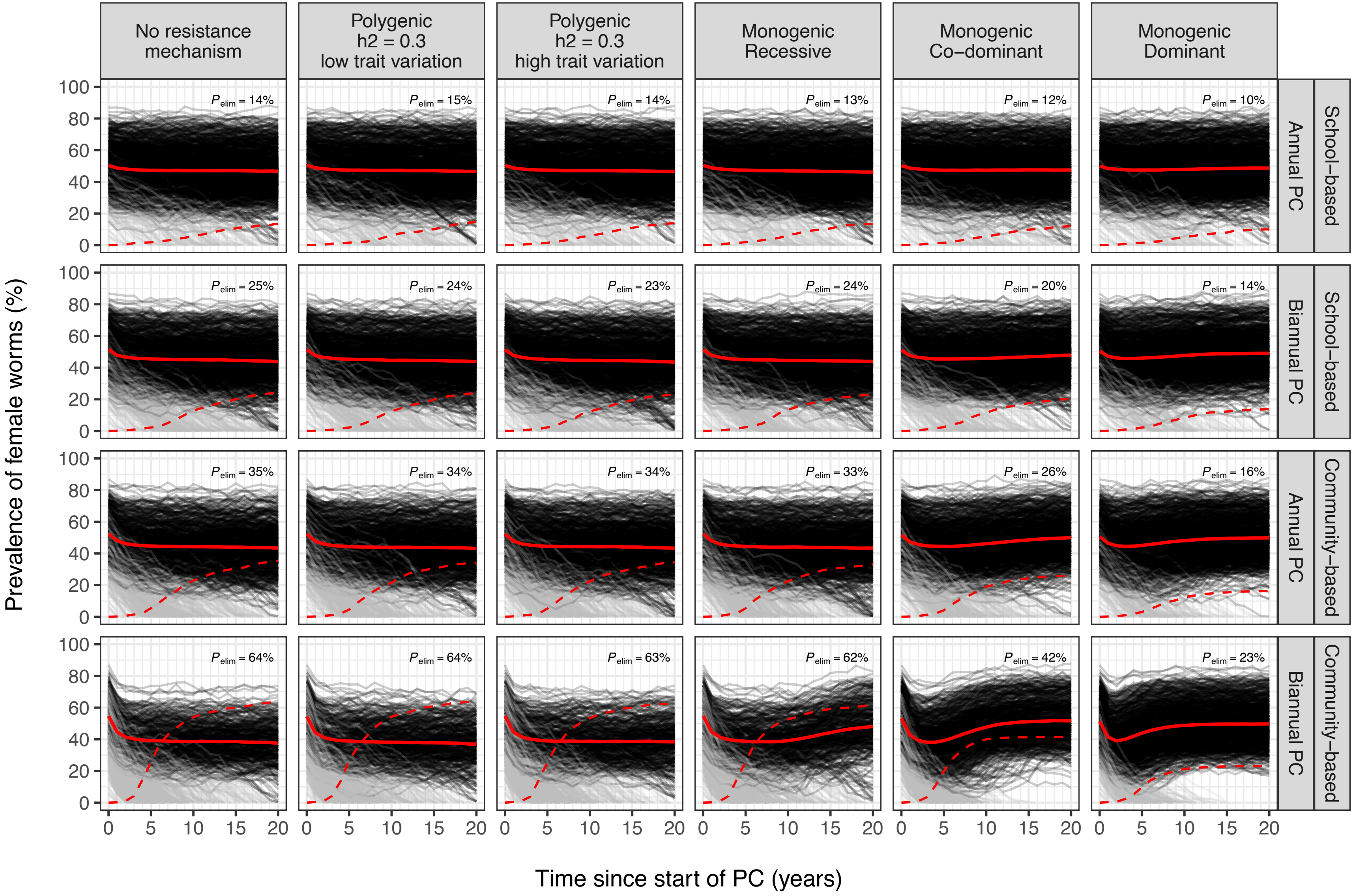

Figure C2. Model-predicted prevalence of female *Necator americanus* worms in the general population over time. Solid red lines indicate the average of the simulations that did not reach elimination; the red dashed lines indicate the proportion of simulations that resulted in elimination over time (i.e., zero female worms left; also indicated for year 20 with P_{elim} in the corner of each panel). Thin lines represent single simulations, where the line colour indicates whether elimination was achieved (grey) or not (black).

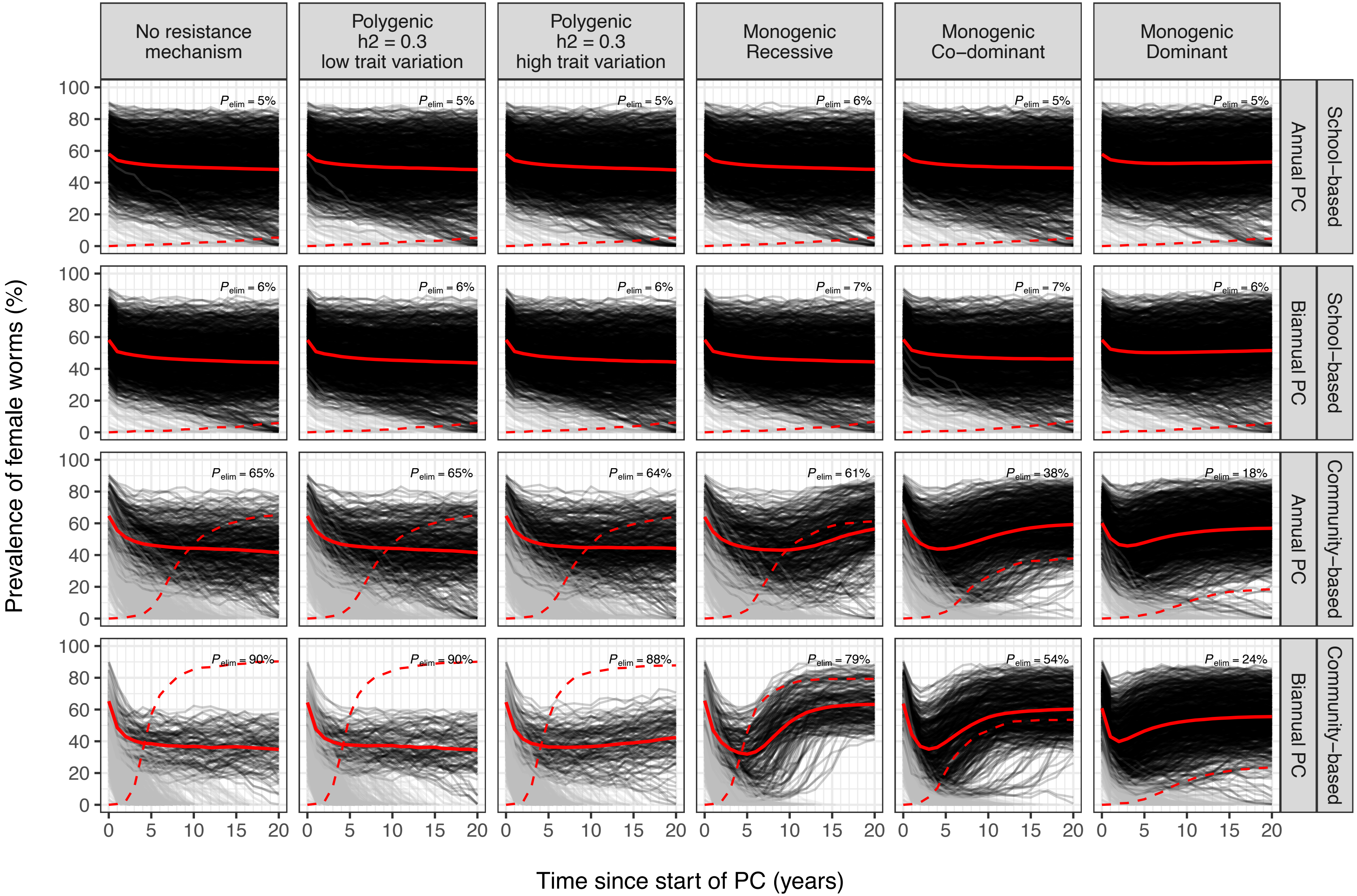

Figure C3. Model-predicted average load of female *Ascaris lumbricoides* worms in the general population over time. Solid red lines indicate the average of the simulations that did not reach elimination. Thin lines represent single simulations, where the line colour indicates whether elimination (i.e., zero female worms left within 20 years of PC) was achieved (grey) or not (black). The proportion of simulations that result in elimination is indicated with P_{elim} in the corner of each panel.

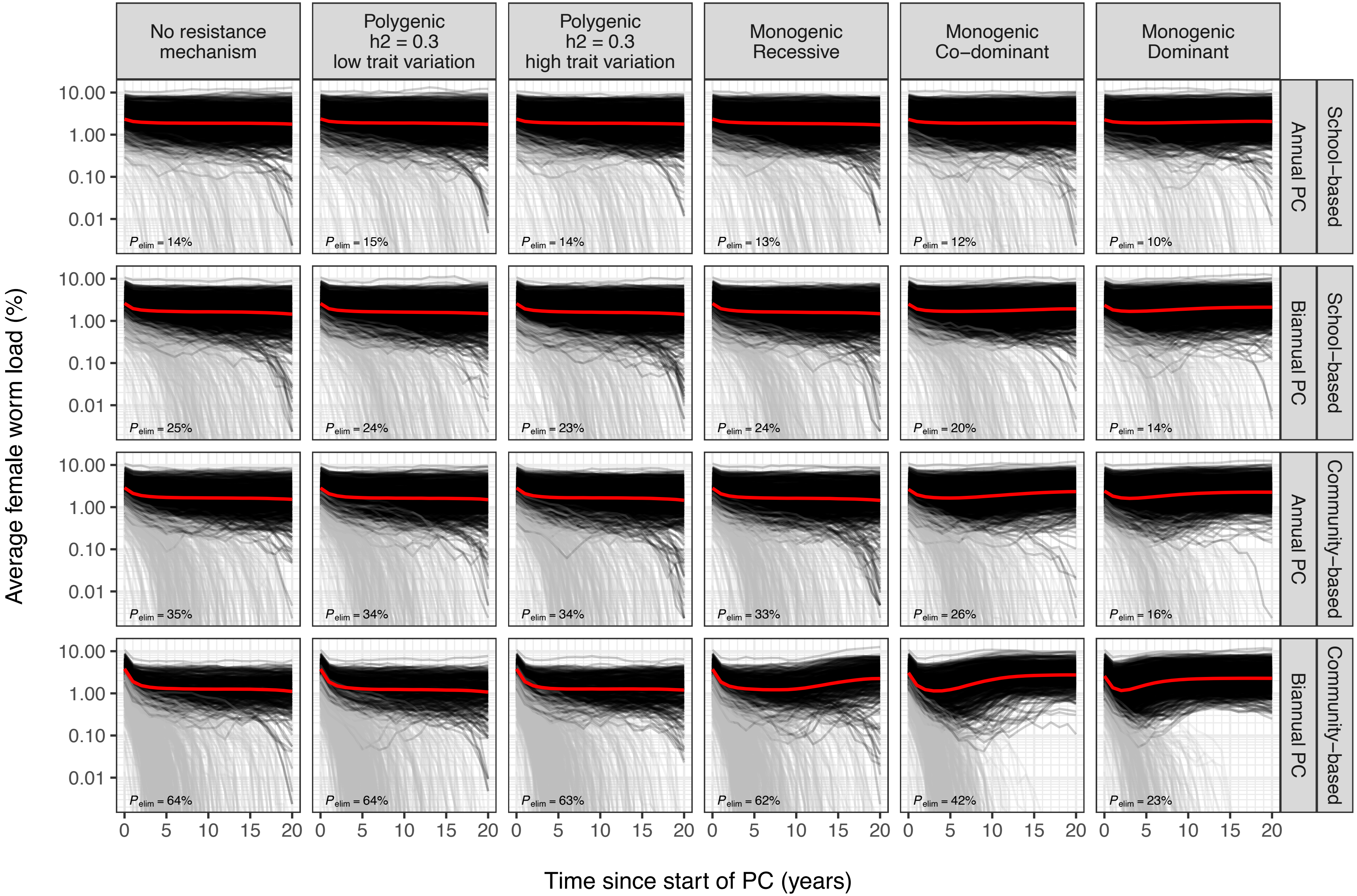

Figure C4. Model-predicted average load of female *Necator americanus* worms in the general population over time. Solid red lines indicate the average of the simulations that did not reach elimination. Thin lines represent single simulations, where the line colour indicates whether elimination (i.e., zero female worms left within 20 years of PC) was achieved (grey) or not (black). The proportion of simulations that result in elimination is indicated with P_{elim} in the corner of each panel.

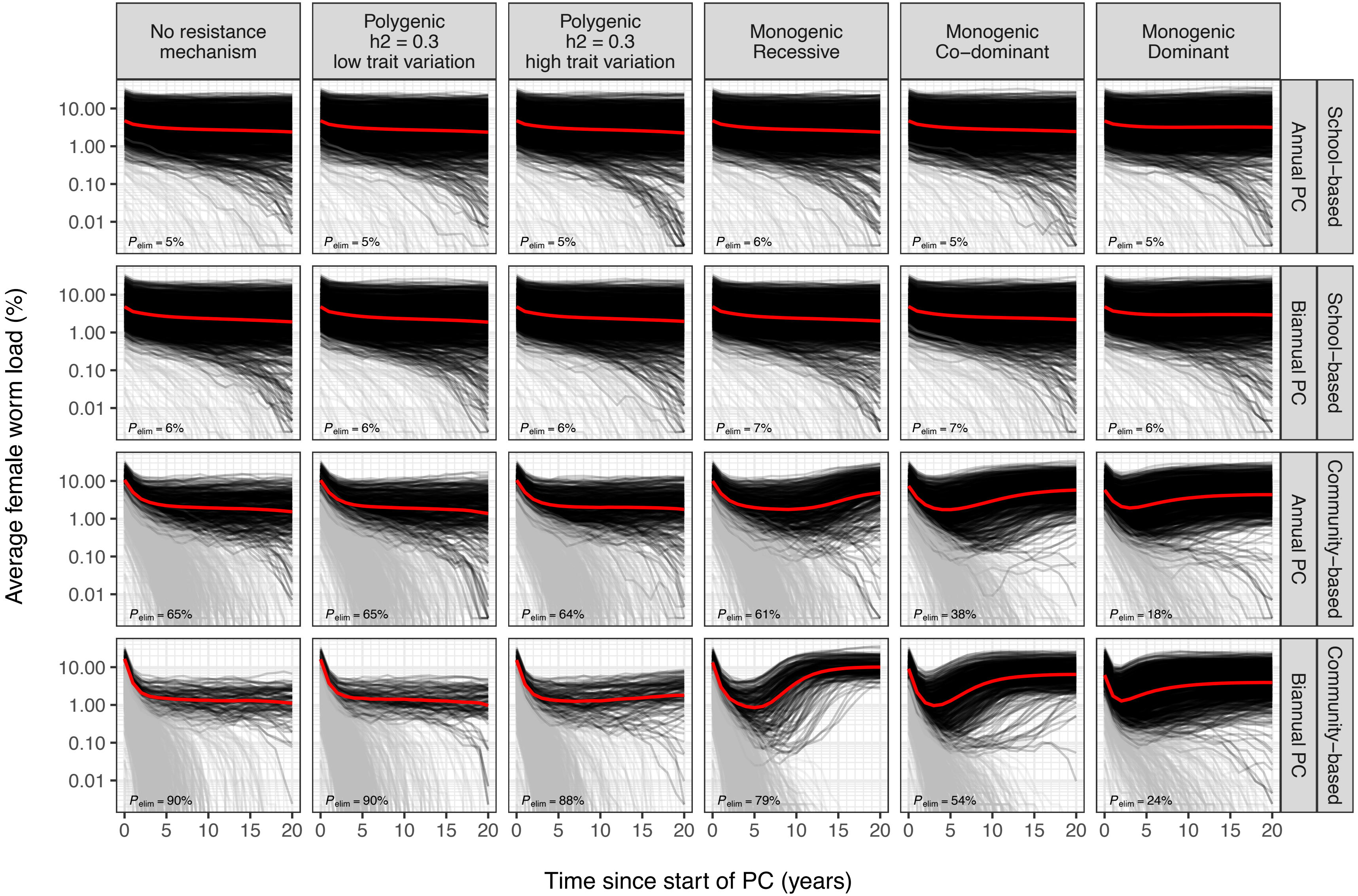

Figure C5. Model-predicted drug efficacy against *Ascaris lumbricoides* worms over time. Solid red lines indicate the average of the simulations that did not reach elimination. Thin lines represent single simulations, where the line colour indicates whether elimination (i.e., zero female worms left within 20 years of PC) was achieved (grey) or not (black). The proportion of simulations that result in elimination is indicated with P_{elim} in the corner of each panel.

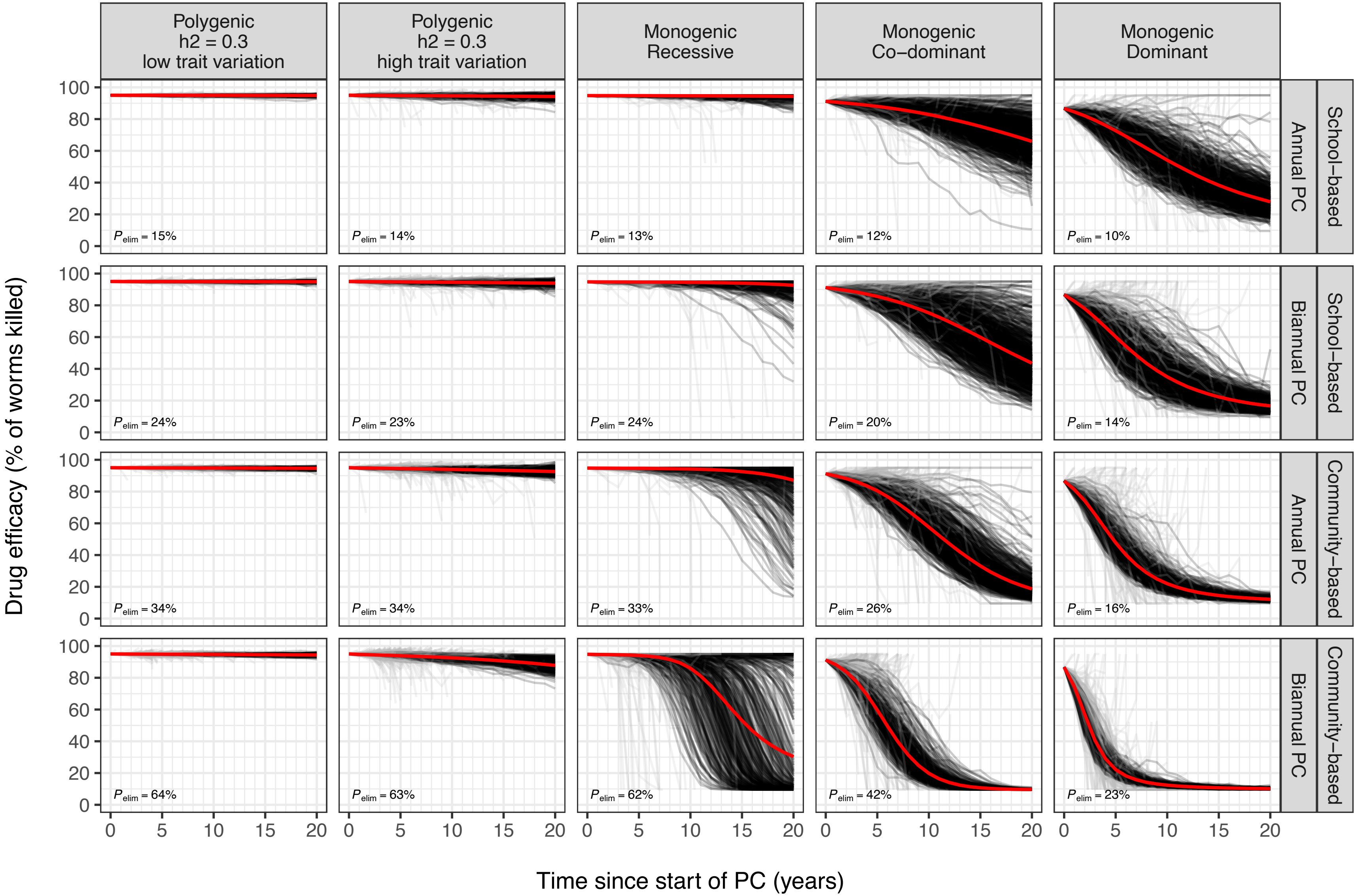

Figure C6. Model-predicted drug efficacy against *Necator americanus* worms over time. Solid red lines indicate the average of the simulations that did not reach elimination. Thin lines represent single simulations, where the line colour indicates whether elimination (i.e., zero female worms left within 20 years of PC) was achieved (grey) or not (black). The proportion of simulations that result in elimination is indicated with P_{elim} in the corner of each panel.

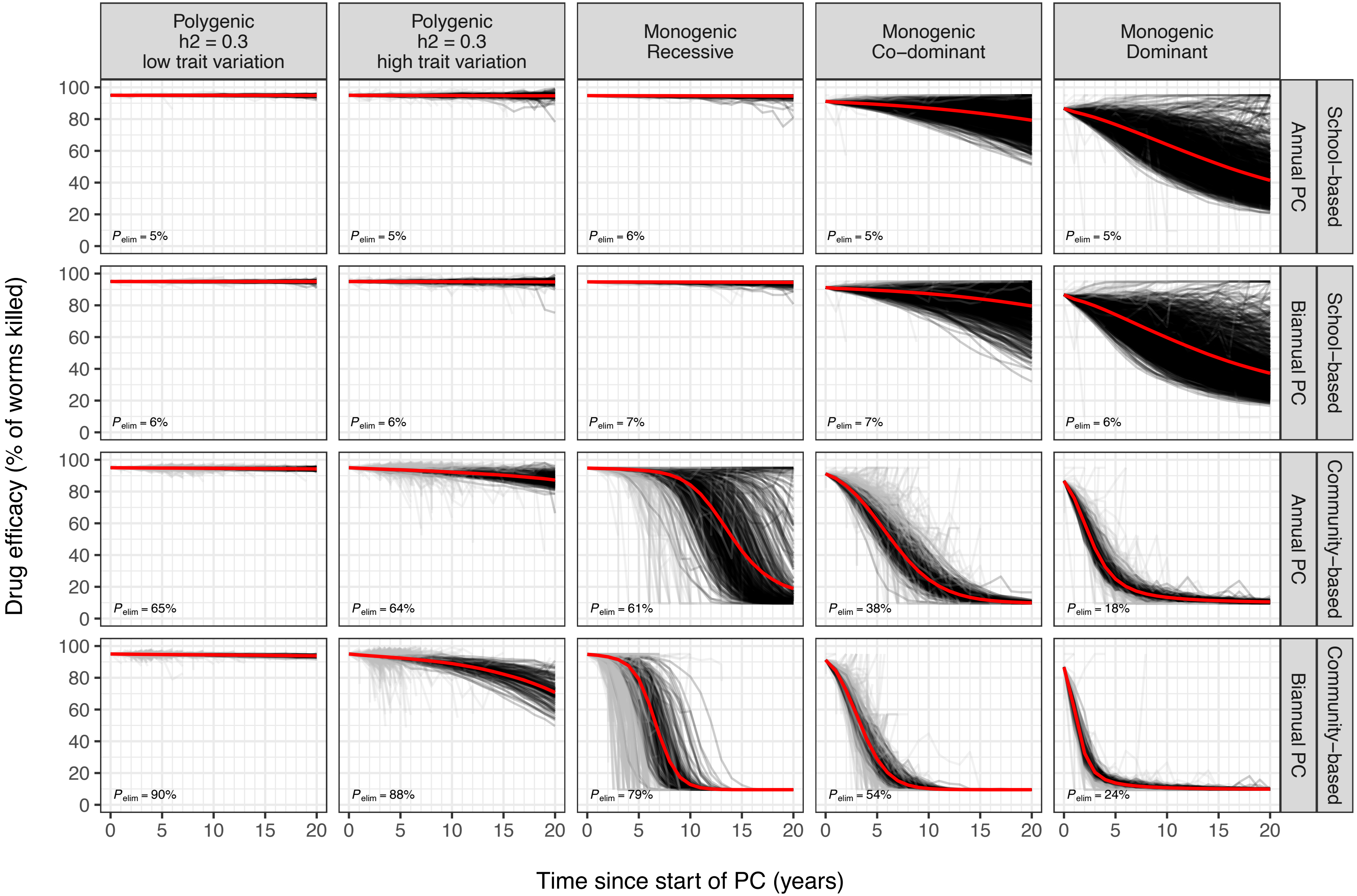

Supplementary Information D: Stratified analyses for transmission conditions

Figure D1. Probability of elimination of *Ascaris lumbricoides*, stratified by parasite population density (x axis) and level of exposure heterogeneity (colours). Stratification was based on tertiles for parasite population density (average number of female worms per person) just before the start of PC and tertiles for exposure heterogeneity in the host population. Elimination was defined as zero female worms before or at 20 years. Absence of visible bars indicates zero probability of elimination. Each bar is based on 1/9 of all 1,000 repeated simulations (i.e., approximately 110 simulations).

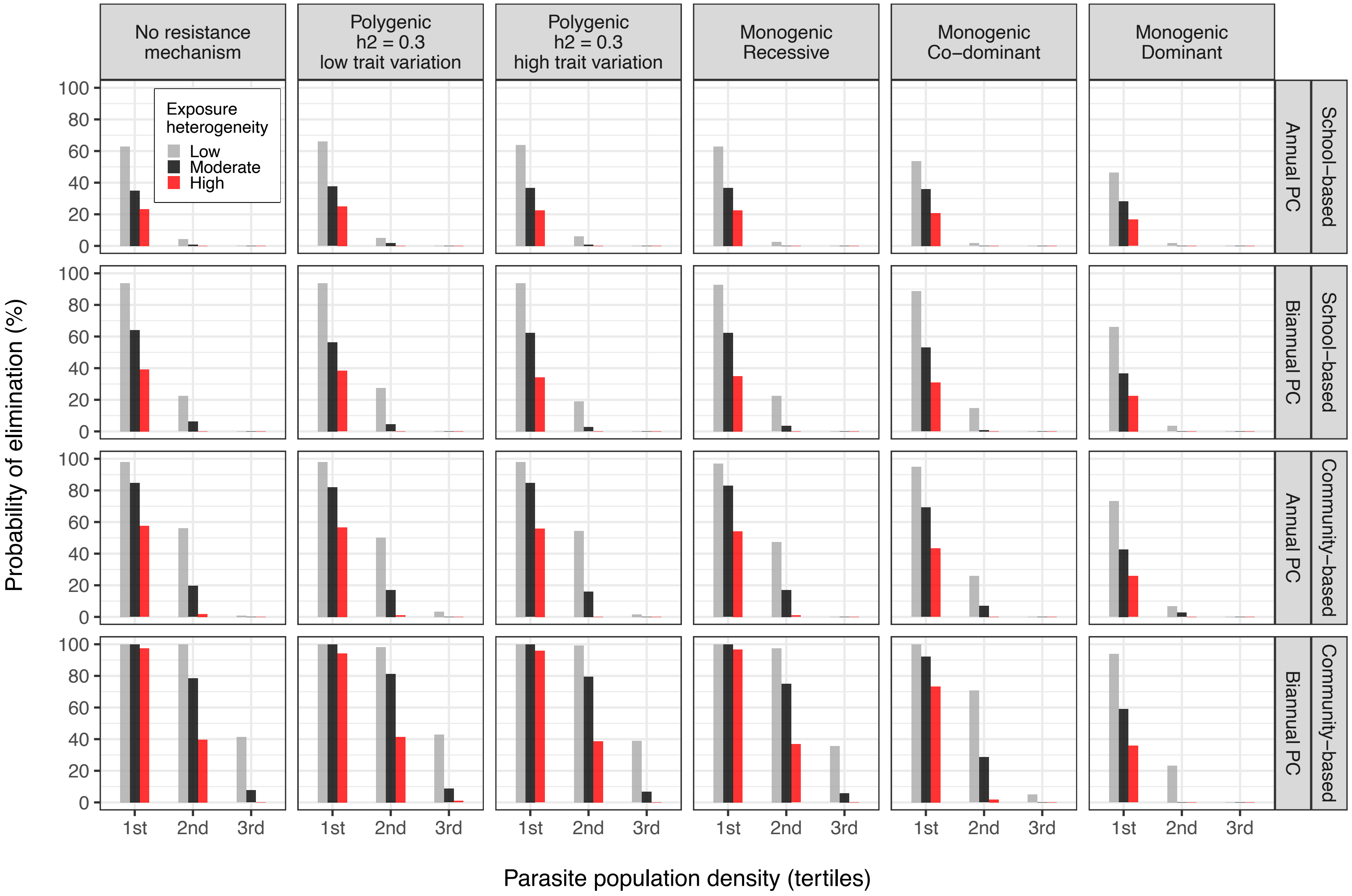

Figure D2. Probability of elimination of *Necator americanus,* stratified by parasite population density (x axis) and level of exposure heterogeneity (colours). Stratification was based on tertiles for parasite population density (average number of female worms per person) just before the start of PC and tertiles for exposure heterogeneity in the host population. Elimination was defined as zero female worms before or at 20 years. Absence of visible bars indicates zero probability of elimination. Each bar is based on 1/9 of all 1,000 repeated simulations (i.e., approximately 110 simulations).

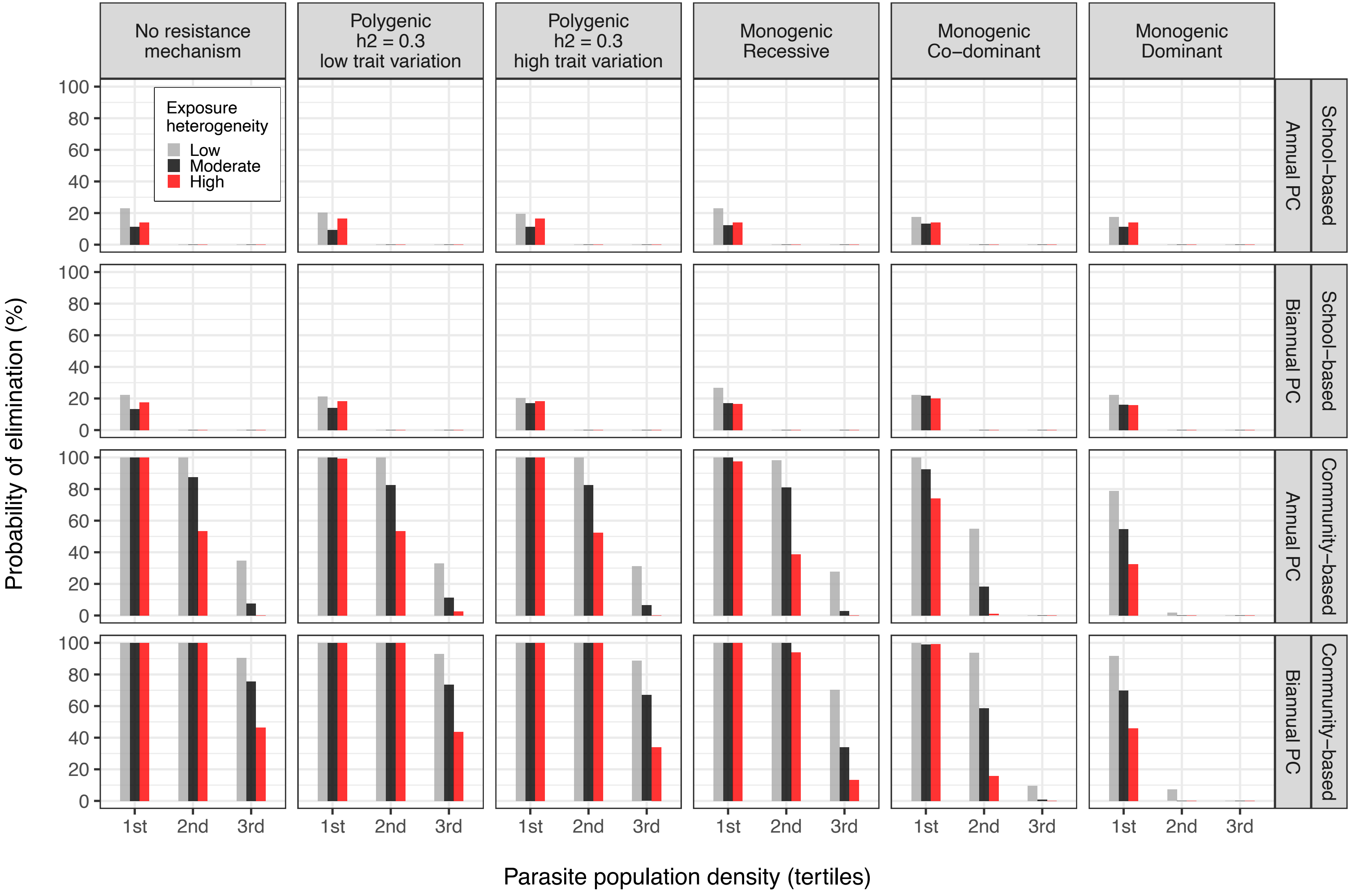

Figure D3. Model-predicted trends in drug efficacy against *Ascaris lumbricoides* in settings where elimination was not achieved, by parasite population density. Lines represent averages per stratum. To make sure trend lines were smooth, averages were only calculated if elimination was not achieved in at least 30 out of about 330 simulations (probability of elimination of less than approximately 90%). Stratification was based on tertiles for parasite population density (average number of female worms per person) just before the start of PC.

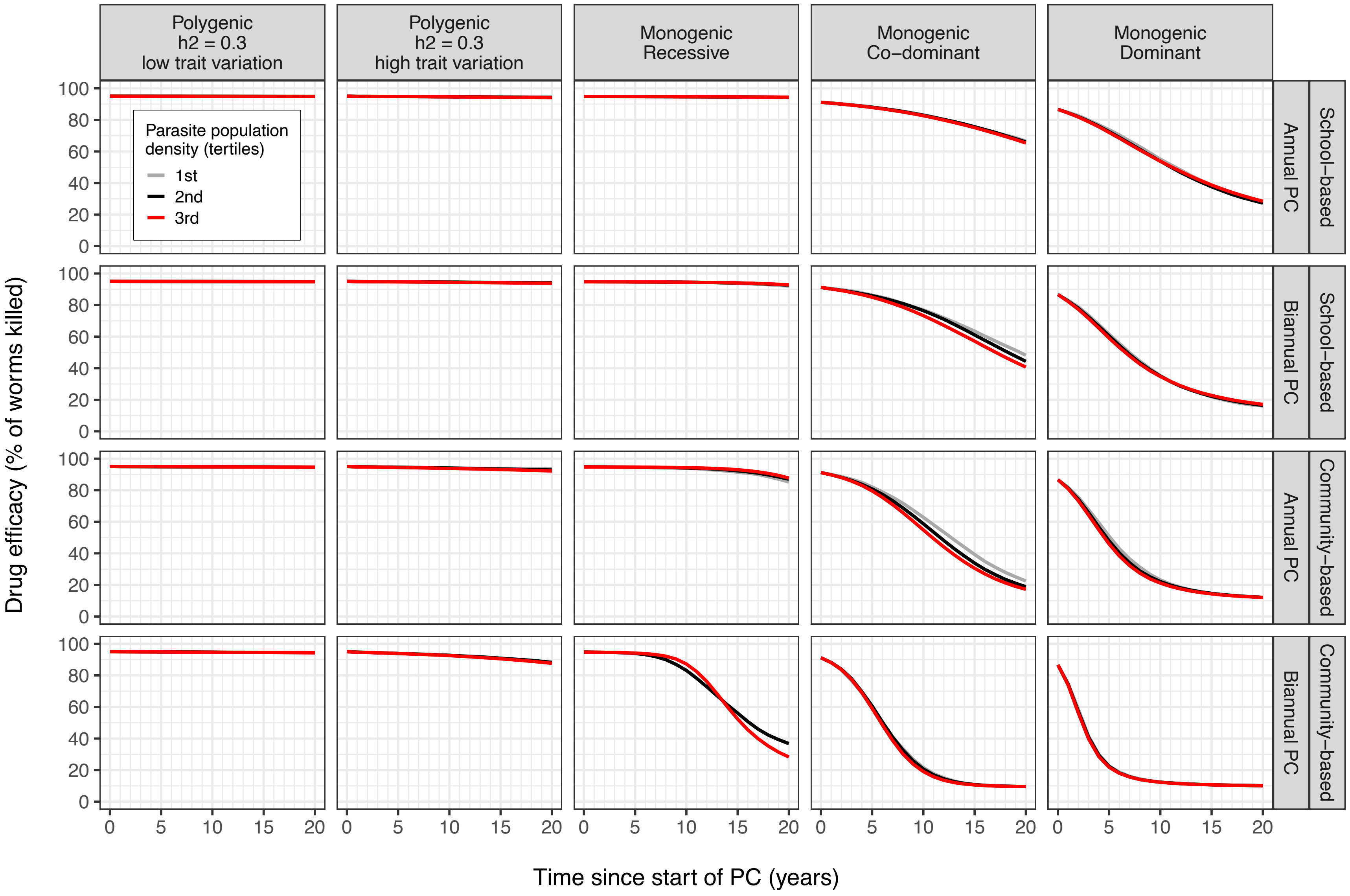

Figure D4. Model-predicted trends in drug efficacy against *Necator americanus* in settings where elimination was not achieved, by parasite population density. Lines represent averages per stratum. To make sure trend lines were smooth, averages were only calculated if elimination was not achieved in at least 30 out of about 330 simulations (probability of elimination of less than approximately 90%). Stratification was based on tertiles for parasite population density (average number of female worms per person) just before the start of PC.

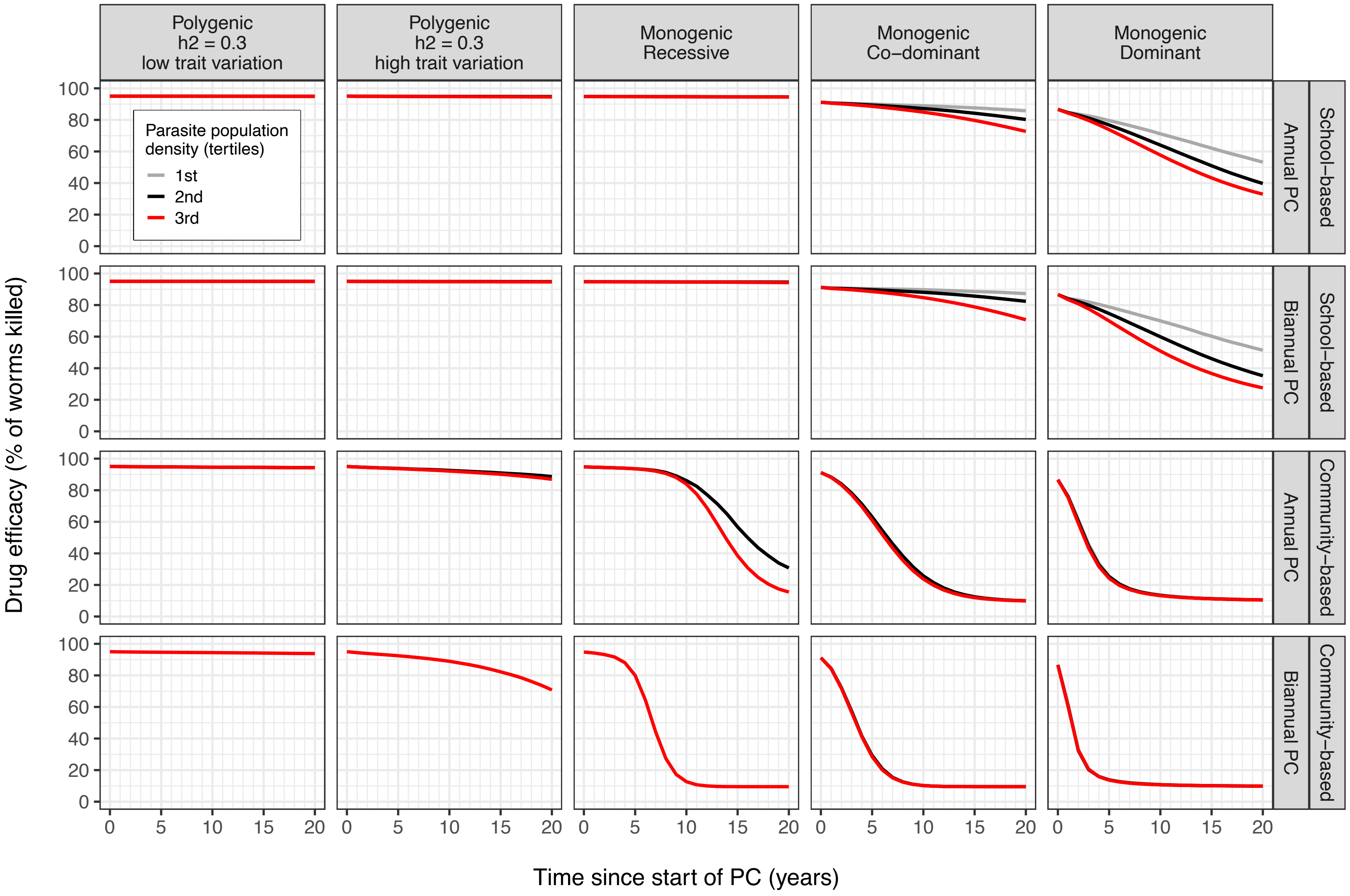

Figure D5. Model-predicted trends in drug efficacy against *Ascaris lumbricoides* in settings where elimination was not achieved, by degree of exposure heterogeneity. Lines represent averages per stratum. To make sure trend lines were smooth, averages were only calculated if elimination was not achieved in at least 30 out of about 330 simulations (probability of elimination of less than approximately 90%). Stratification was based on tertiles for exposure heterogeneity in the host population.

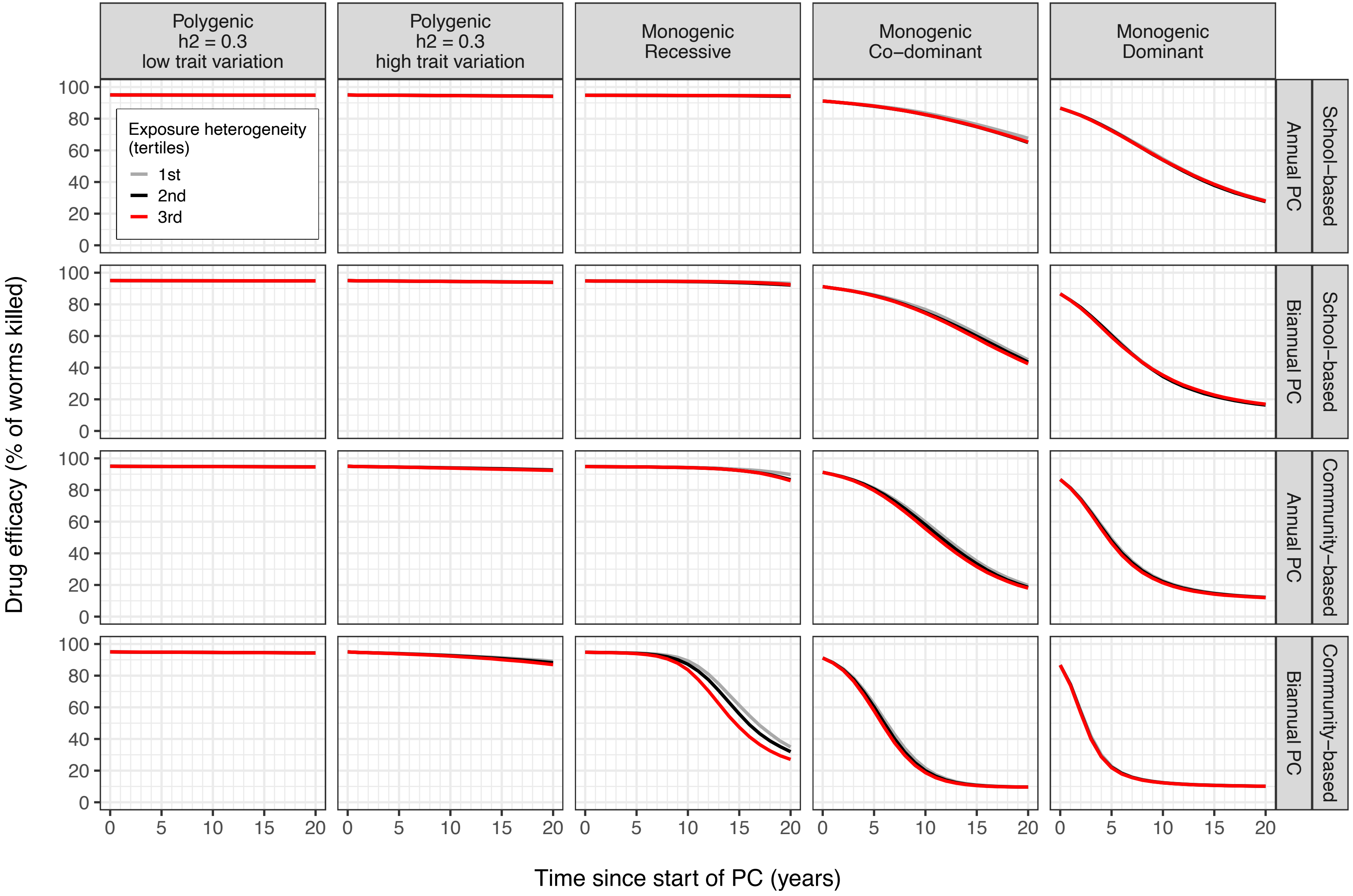

Figure D6. Model-predicted trends in drug efficacy against *Necator americanus* in settings where elimination was not achieved, by degree of exposure heterogeneity. Lines represent averages per stratum. To make sure trend lines were smooth, averages were only calculated if elimination was not achieved in at least 30 out of about 330 simulations (probability of elimination of less than approximately 90%). Stratification was based on tertiles for exposure heterogeneity in the host population.

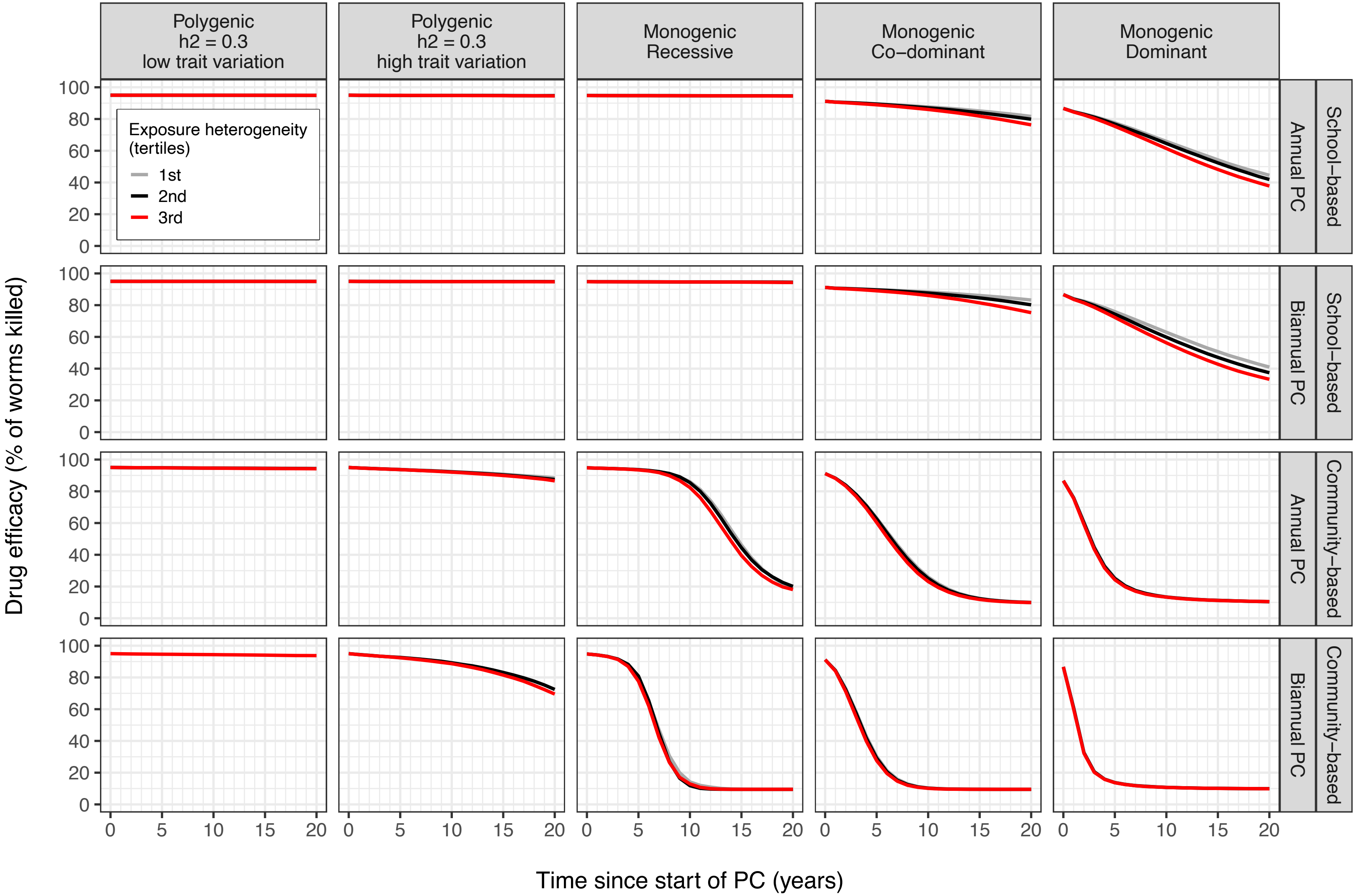

Supplementary Information E: Sensitivity analyses

Figure E1. Monogenic resistance: impact of initial allele frequencies on trends in drug efficacy during annual community-based PC. Solid red lines indicate the average of the simulations that did not reach elimination; thin black lines are individual simulations in none of which elimination (i.e., zero female worms left within 20 years of PC) was achieved. The proportion of simulations that eventually resulted in elimination is indicated with $\boldsymbol{P}_{\text{elim}}$ in the corner of each panel.

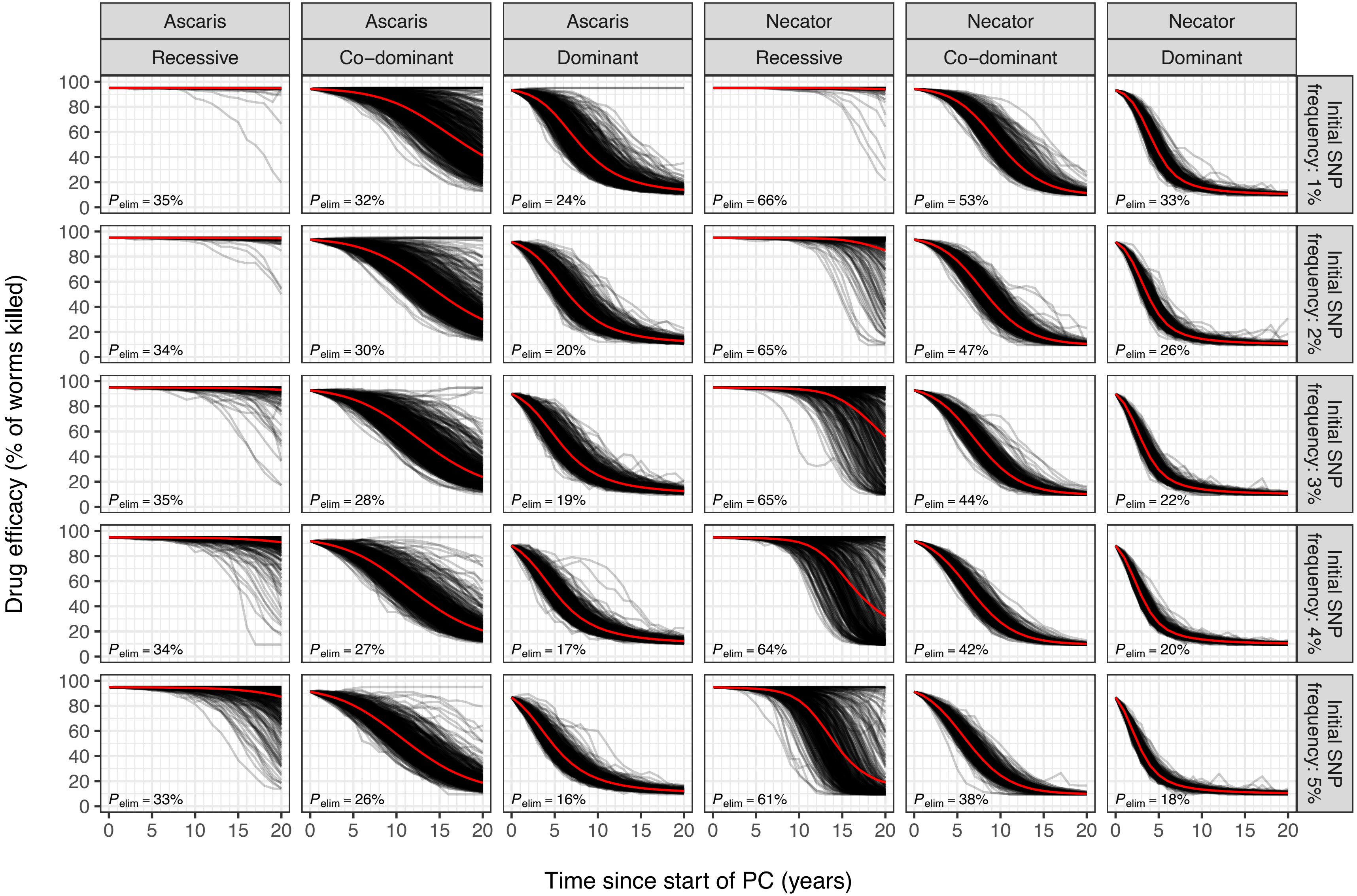

Figure E2. Summary of impact of initial allele frequency on model-predicted trends in drug efficacy during annual community-based PC in case of monogenic drug resistance. Lines are the same as the red lines for average drug efficacy in Figure E1 and therefore represent settings where elimination was not achieved within 20 years.

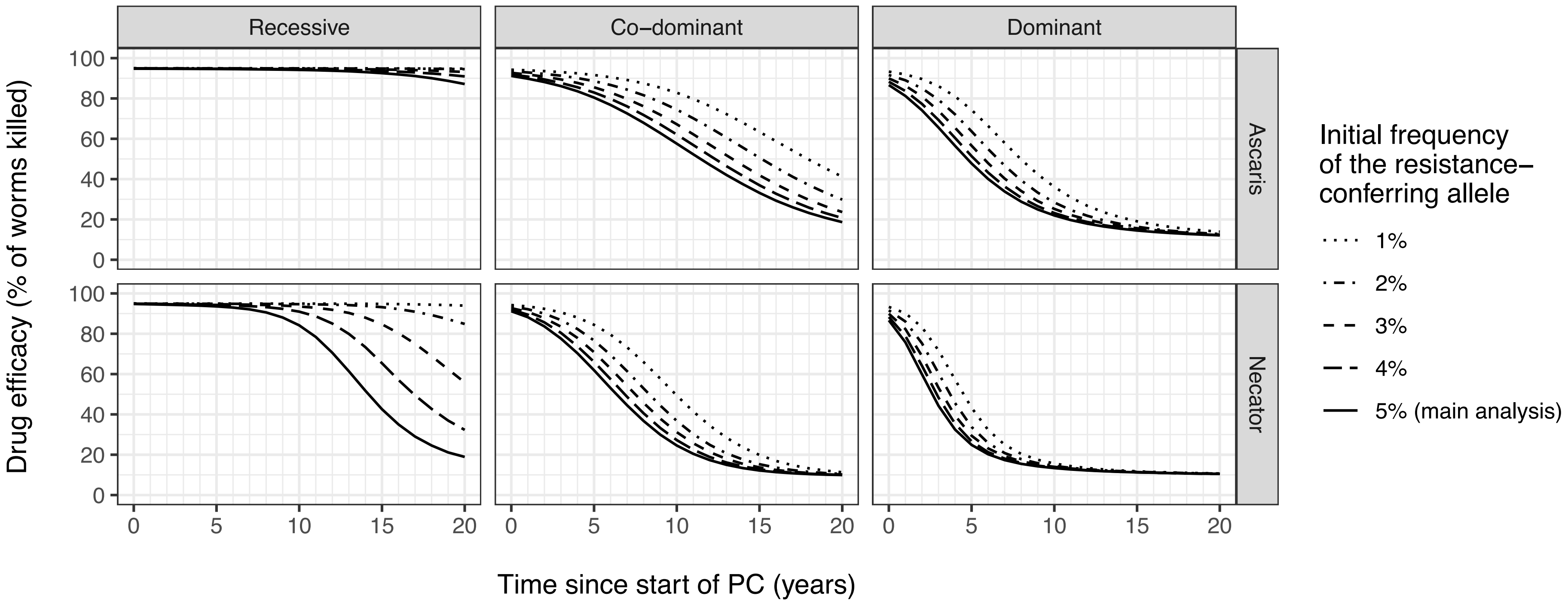

Figure E3. Polygenic resistance: impact of heritability (h^2) on trends in drug efficacy. Solid red lines indicate the average of the simulations that did not reach elimination; thin black lines are individual simulations in none of which elimination (i.e., zero female worms left within 20 years of PC) was achieved. The proportion of simulations that eventually resulted in elimination is indicated with P_{elim} in the corner of each panel.

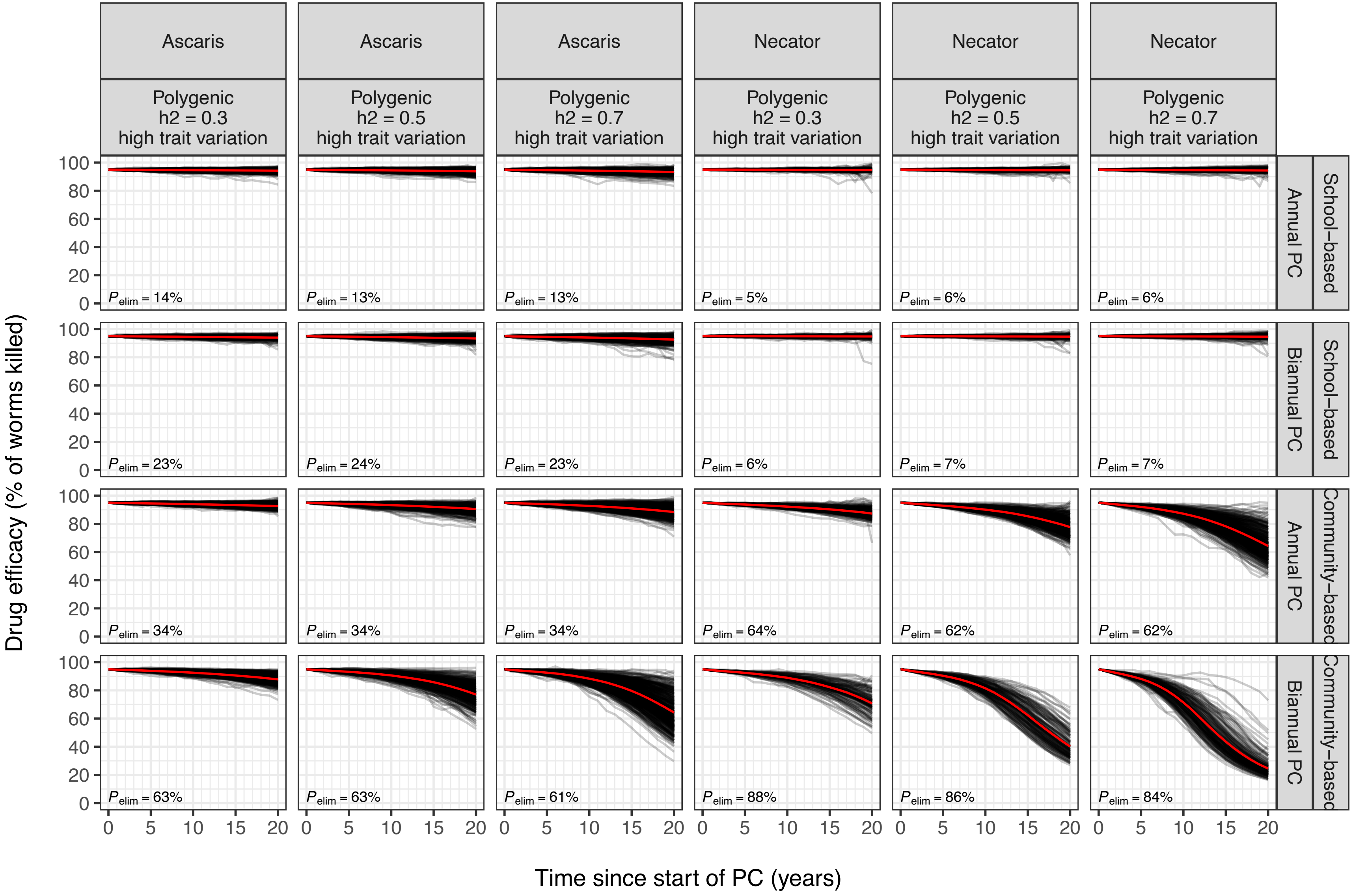

Figure E4. Summary of impact of heritability (h^2) on trends in drug efficacy. Lines are the same as the red lines for average drug efficacy in Figure E3 and therefore represent settings where elimination was not achieved within 20 years.

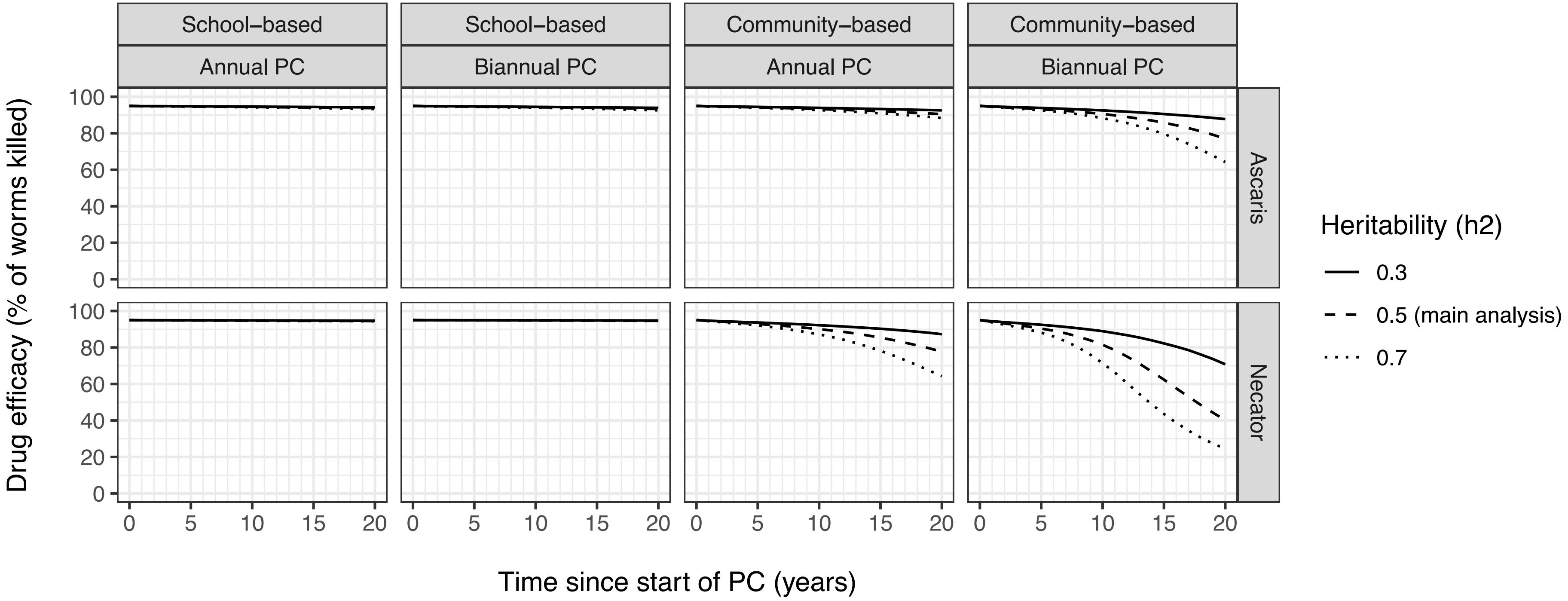

Figure E5. Monogenic resistance with co-dominant inheritance: impact of drug efficacy against non-resistant worms (genotype aa) on trends in drug efficacy. Solid red lines indicate the average of the simulations that did not reach elimination; thin black lines are individual simulations in none of which elimination (i.e., zero female worms left within 20 years of PC) was achieved. The proportion of simulations that eventually resulted in elimination is indicated with $\boldsymbol{P}_{\text{elim}}$ in the corner of each panel. As in the main analysis, we assume that drug efficacy against fully and partially resistant phenotypes is reduced by 90% and 60%, respectively, compared to drug efficacy against non-resistant worms.

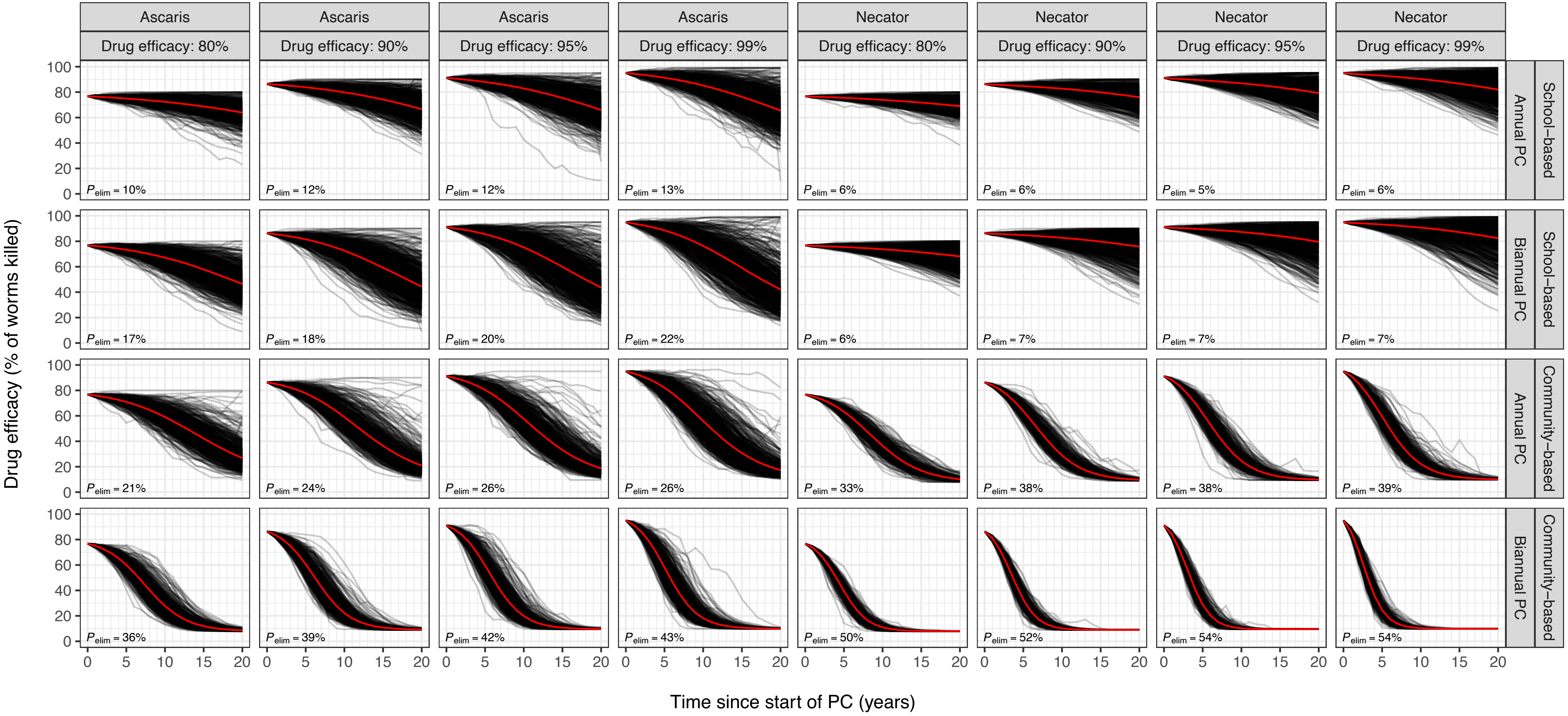

Figure E6. Summary impact of drug efficacy against non-resistant parasites on model-predicted trends in drug efficacy. Lines are the same as the red lines for average drug efficacy in Figure E5 and therefore represent settings where elimination was not achieved within 20 years. Drug resistance was assumed to be monogenic with co-dominant inheritance.

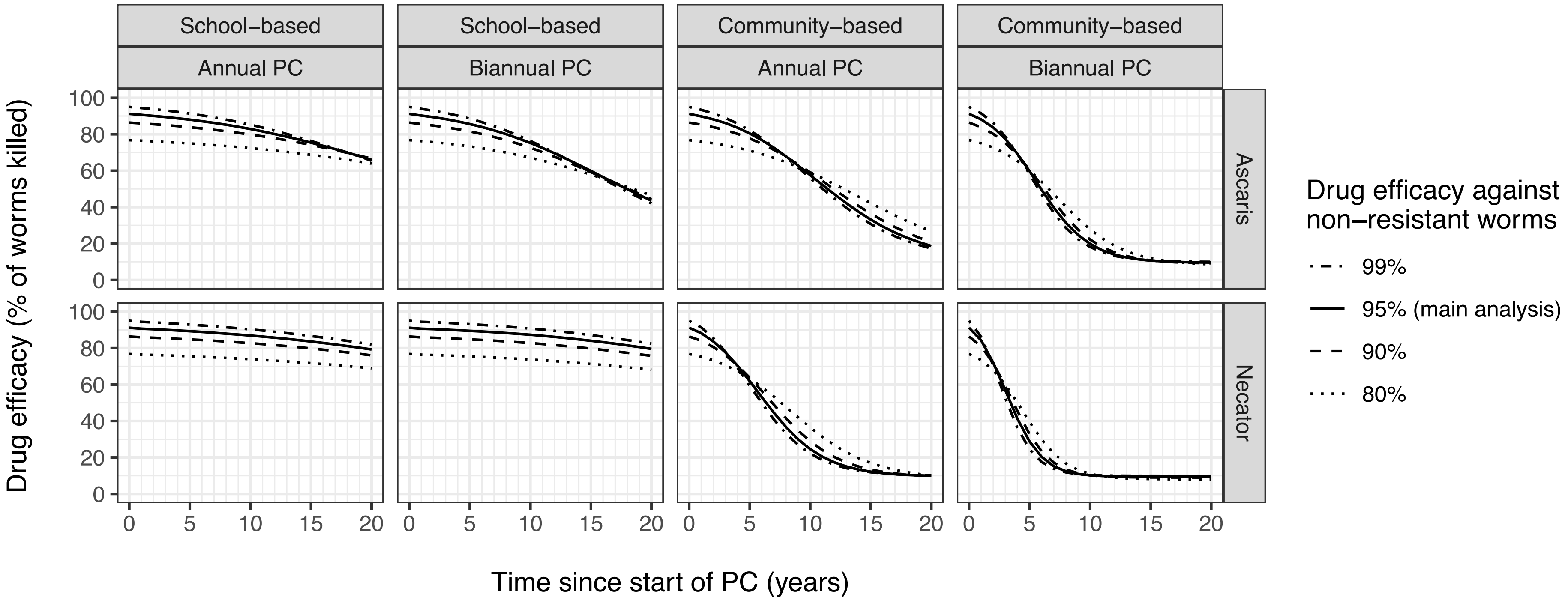

Figure E7. Impact of PC coverage and patterns in individual PC uptake on trends in drug efficacy (co-dominant resistance) during annual community-based PC. PC coverage is defined as the percentage of the targeted population (age 2 and above) that takes up PC every round. Random PC uptake means that individual uptake of PC is not correlated over time such that after 10 rounds, >99.9% of individuals that were eligible throughout those 10 years have received at least one treatment. In contrast, "5% never treated" means that after 10 rounds, due to correlation in uptake over time, 5% of eligible individuals will have never received a treatment. "Systematic PC uptake" is the extreme scenario in which always the same individuals are treated such that the proportion never treated is 100% minus the coverage. Solid red lines indicate the average of the simulations that did not reach elimination; thin black lines are individual simulations in none of which elimination (i.e., zero female worms left within 20 years of PC) was achieved. The proportion of simulations that eventually resulted in elimination is indicated with $\boldsymbol{P}_{\text{elim}}$ in the corner of each panel.

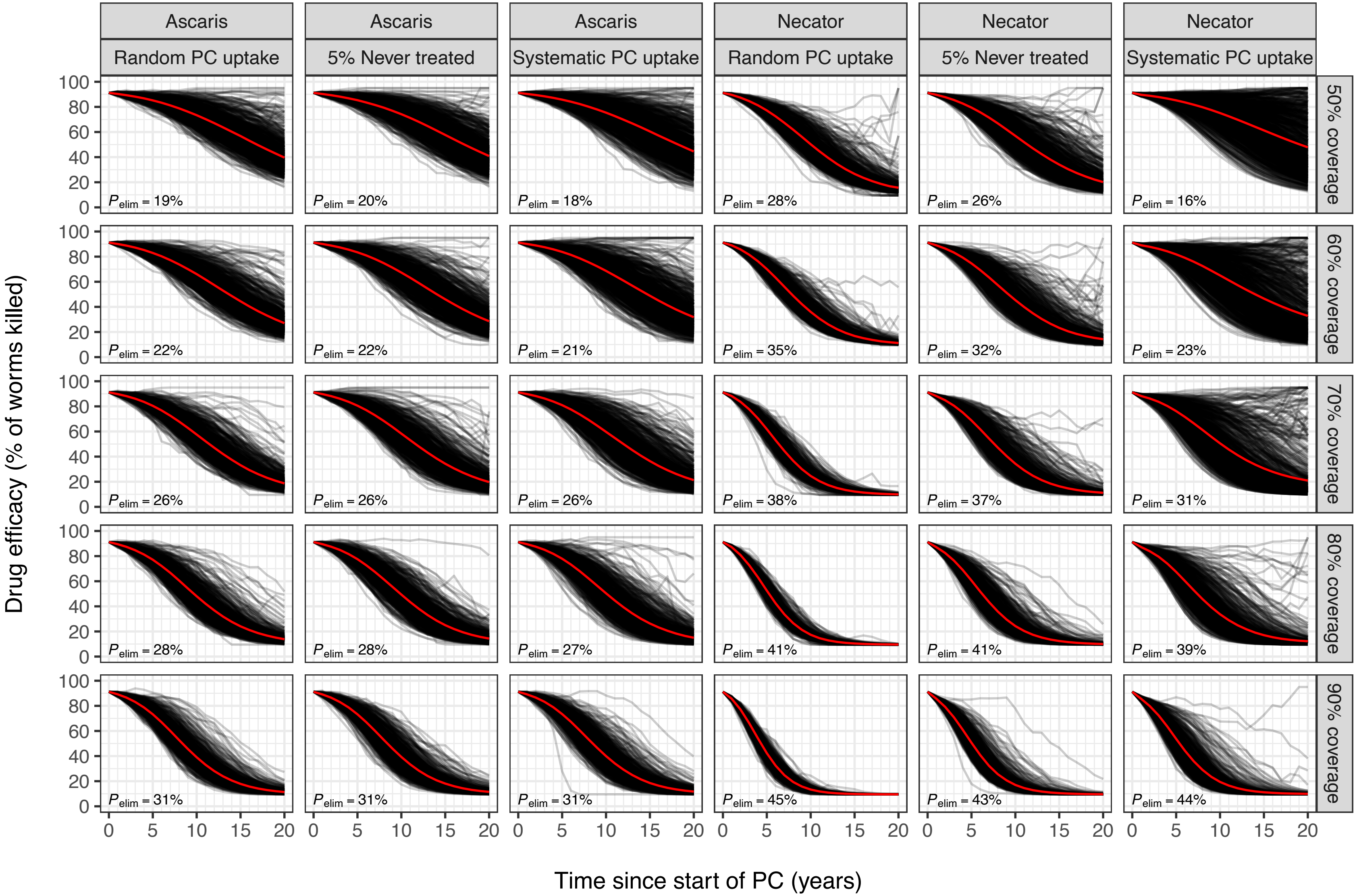

Figure E8. Summary of impact of PC coverage and patterns in individual PC uptake on prospects of elimination and trends in drug efficacy (co-dominant resistance) during annual community-based PC. PC coverage is defined in terms of percentage of the targeted population (age 2 and above) that takes up PC every round. Random PC uptake means that individual uptake of PC is not correlated over time, such that after 10 rounds >99.9% of individuals that were eligible throughout those 10 years have received at least one treatment. In contrast, "fully systematic non-participation" is the extreme scenario in which always the same individuals are treated such that the proportion never treated is 100% minus the coverage. In between, we define a scenario with "5% never treated" in which after 10 rounds, due to correlation in individual uptake over time, 5% of eligible individuals will have never received a treatment. Black lines are the same as the red lines for average drug efficacy in Figure E7 and therefore represent settings where elimination was not achieved within 20 years.

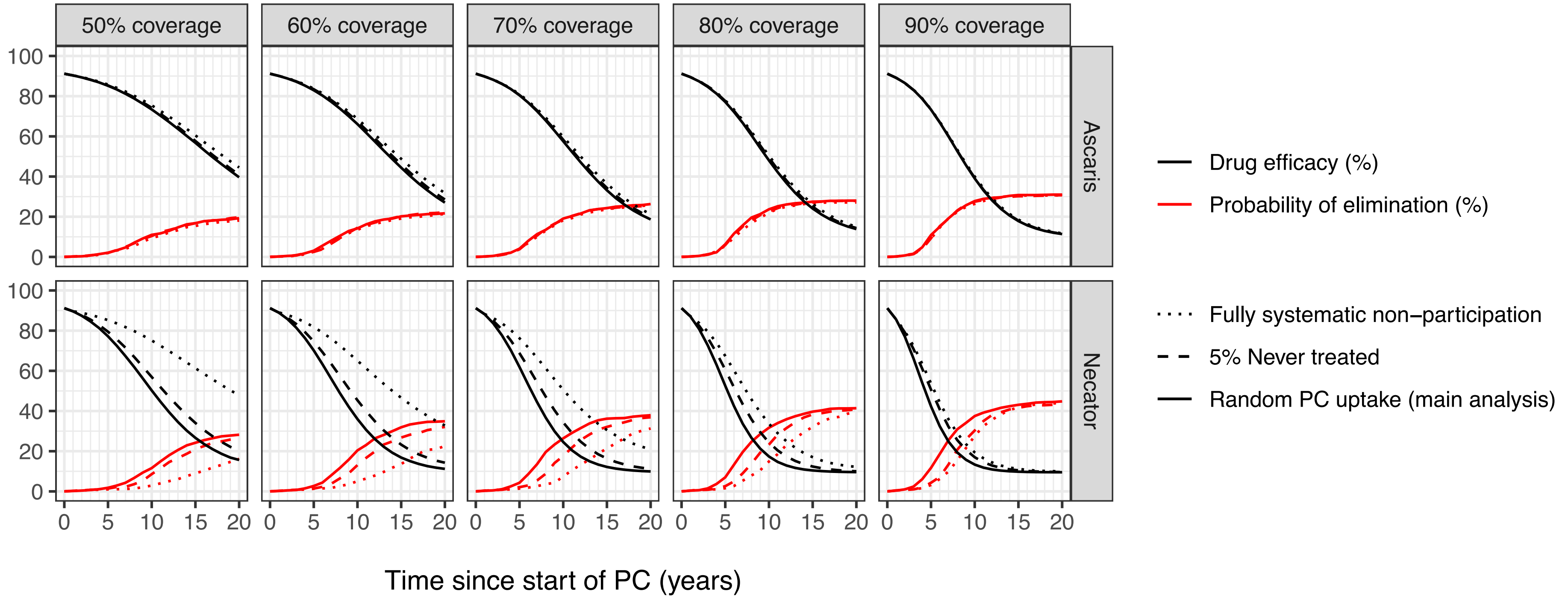

Figure E9. Impact of fitness loss on trends in drug efficacy in case of monogenic resistance and annual community-wide PC. The fitness loss was defined as a 1/3 reduction in female egg productivity for fully resistant worms (recessive and dominant resistance) and a 1/6 reduction for partially resistant worms (co-dominant resistance only). Solid red lines indicate the average of the simulations that did not reach elimination; thin black lines are individual simulations in none of which elimination (i.e., zero female worms left within 20 years of PC) was achieved. The proportion of simulations that eventually resulted in elimination is indicated with $\boldsymbol{P}_{\mathrm{elim}}$ in the corner of each panel.

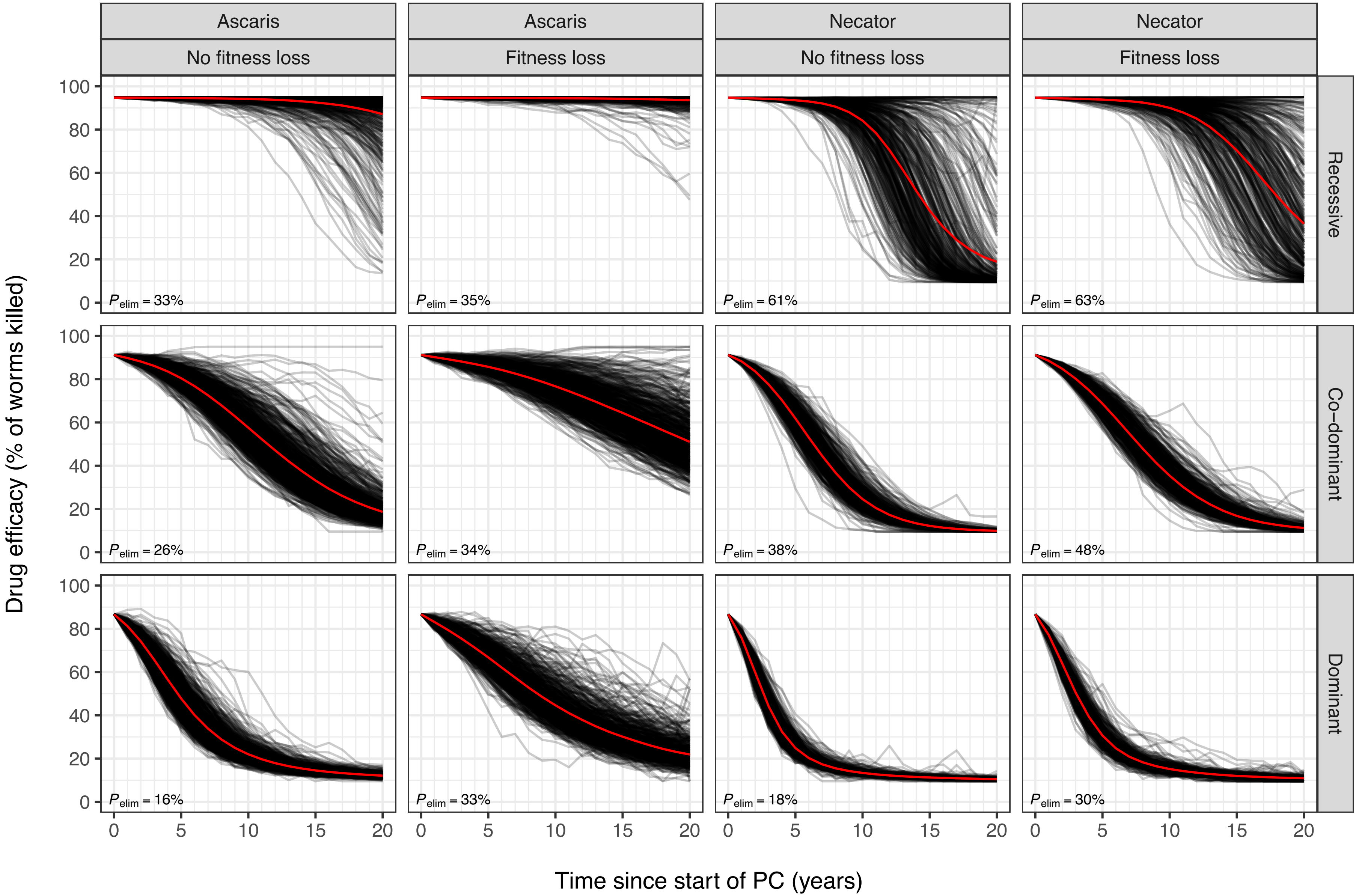

Figure E10. Summary of impact of fitness loss on trends in drug efficacy in case of monogenic resistance and annual community-wide PC. The fitness loss was defined as a 1/3 reduction in female egg productivity for fully resistant worms (recessive and dominant resistance) and a 1/6 reduction for partially resistant worms (co-dominant resistance only). Lines are the same as the red lines for average drug efficacy in Figure E9 and therefore represent settings where elimination was not achieved within 20 years.

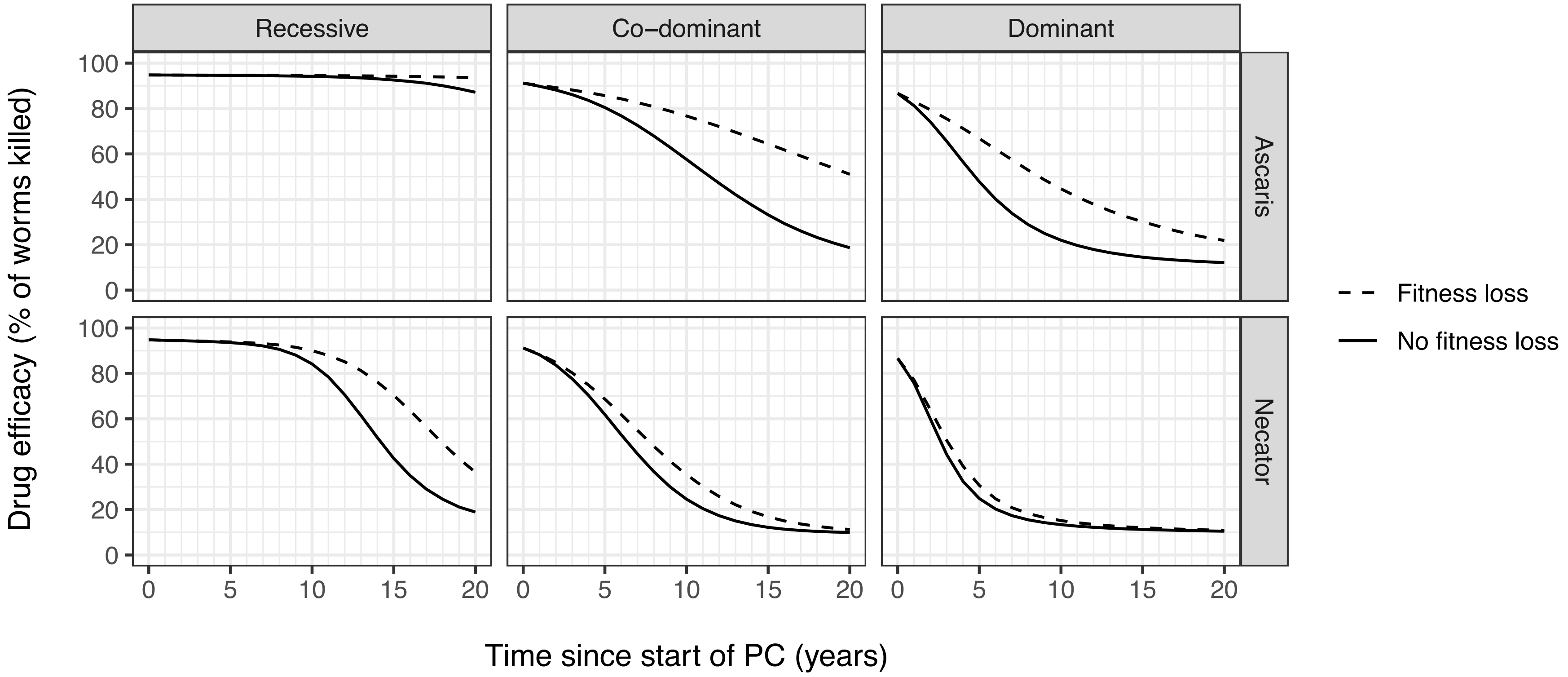

Supplementary Information F: Predictive value of measured faecal egg reduction rates

Figure F1. Predictive value of faecal egg reduction rate (ERR) under 85% for a true drug efficacy under 90% during annual community-based PC. For illustrative purposes, the initial frequency of resistant alleles was assumed to be low (1%, as in Figure 3 in the main manuscript), such that before the start of PC (time zero), the average true drug efficacy (red solid line) was very close to 95% (i.e., proportion of worms killed). The probability of finding an ERR <85% (solid teal line) was based on a simulated survey of 1,000 individuals treated via PC (i.e., all ages of 2 and above), where individuals were tested with a single Kato-Katz (KK) before treatment and duplicate KK (based on a single fecal sample) two weeks after treatment. ERRs were calculated based on the ratio of pre- and post-treatment arithmetic mean egg counts of all tested individuals. Dashed lines represent positive and negative predictive values (PPV and NPV, respectively) of detection an ERR under or above 85% as an indicator of true drug efficacy under or above 90% (i.e., proportion of worms killed). The initial decline in the chance of finding an ERR <85% (teal solid line) is due to the fact that early on during PC programs, there are still relatively many pre-patent worms that are not affected by treatment but who become patent and start producing eggs between the moment of treatment and the post-treatment survey two weeks later.

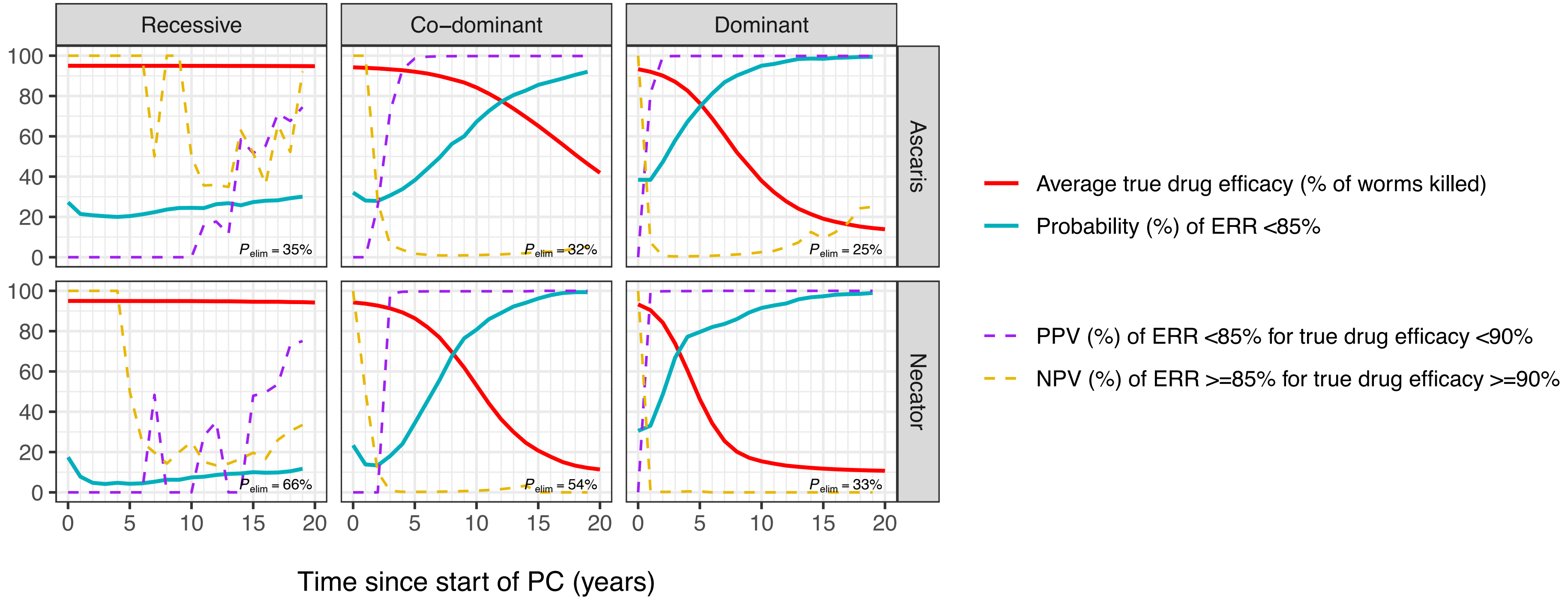

Supplement: Supplementary file 1 — Supplementary Information [file 41467_2024_45027_MOESM1_ESM.pdf]
