# Supplementary material for: Predicting the risk and speed of drug resistance emerging in soil-transmitted helminths during preventive chemotherapy
Source: Nat Commun. 2024 Feb 6;15:1099. doi: 10.1038/s41467-024-45027-2 (PMC10847116; doi:10.1038/s41467-024-45027-2)
Supplement: Supplementary file 3 — Reporting Summary [file 41467_2024_45027_MOESM3_ESM.pdf]

nature portfolio

Corresponding author(s): Luc E. Coffeng

Last updated by author(s): Sep 20, 2023

Reporting Summary

Nature Portfolio wishes to improve the reproducibility of the work that we publish. This form provides structure for consistency and transparency in reporting. For further information on Nature Portfolio policies, see our Editorial Policies and the Editorial Policy Checklist.

Statistics

For all statistical analyses, confirm that the following items are present in the figure legend, table legend, main text, or Methods section.

n/a	Confirmed	
☐	☒	The exact sample size (*n*) for each experimental group/condition, given as a discrete number and unit of measurement
☒	☐	A statement on whether measurements were taken from distinct samples or whether the same sample was measured repeatedly
☒	☐	The statistical test(s) used AND whether they are one- or two-sided<br>*Only common tests should be described solely by name; describe more complex techniques in the Methods section.*
☒	☐	A description of all covariates tested
☐	☒	A description of any assumptions or corrections, such as tests of normality and adjustment for multiple comparisons
☐	☒	A full description of the statistical parameters including central tendency (e.g. means) or other basic estimates (e.g. regression coefficient) AND variation (e.g. standard deviation) or associated estimates of uncertainty (e.g. confidence intervals)
☒	☐	For null hypothesis testing, the test statistic (e.g. *F*, *t*, *r*) with confidence intervals, effect sizes, degrees of freedom and *P* value noted<br>*Give P values as exact values whenever suitable.*
☒	☐	For Bayesian analysis, information on the choice of priors and Markov chain Monte Carlo settings
☒	☐	For hierarchical and complex designs, identification of the appropriate level for tests and full reporting of outcomes
☒	☐	Estimates of effect sizes (e.g. Cohen's *d*, Pearson's *r*), indicating how they were calculated

Our web collection on statistics for biologists contains articles on many of the points above.

Software and code

Policy information about availability of computer code

Data collection	The simulation model and model predictions were generated using custom code developed in R (v4.3.1, compatible with v3.6.0 or higher). The model source code is made available as the R package "simresist (v1.1.1)", which is available from https://doi.org/10.5281/zenodo.10442358 (frozen version used for the paper) and https://gitlab.com/luccoffeng/simresist (the live repository which may include future updates). The simresist package depends on the open-source R package "data.table" (v1.12.8 or higher). The simresist package is open-source, licensed under CC-BY 4.0 (https://creativecommons.org/licenses/by/4.0/).
Data analysis	Model predictions were processed, analysed, and visualised using R (v4.3.1) via Rstudio (v2023.06.1+524). Visualisations were produced using the open-source R package "ggplot2" (v3.4.2).

For manuscripts utilizing custom algorithms or software that are central to the research but not yet described in published literature, software must be made available to editors and reviewers. We strongly encourage code deposition in a community repository (e.g. GitHub). See the Nature Portfolio guidelines for submitting code & software for further information.

Data

Policy information about availability of data

All manuscripts must include a data availability statement. This statement should provide the following information, where applicable:

- Accession codes, unique identifiers, or web links for publicly available datasets
- A description of any restrictions on data availability
- For clinical datasets or third party data, please ensure that the statement adheres to our policy

The model-simulated data that support the findings of this study are available at https://doi.org/10.6084/m9.figshare.24521431. The code to generate and process it the simulated data are available at https://gitlab.com/luccoffeng/simresist-nature-comms-sth-paper.

Research involving human participants, their data, or biological material

Policy information about studies with human participants or human data. See also policy information about sex, gender (identity/presentation), and sexual orientation and race, ethnicity and racism.

Reporting on sex and gender	Not applicable
Reporting on race, ethnicity, or other socially relevant groupings	Not applicable
Population characteristics	Not applicable
Recruitment	Not applicable
Ethics oversight	Not applicable

Note that full information on the approval of the study protocol must also be provided in the manuscript.

Field-specific reporting

Please select the one below that is the best fit for your research. If you are not sure, read the appropriate sections before making your selection.

☒ Life sciences ☐ Behavioural & social sciences ☐ Ecological, evolutionary & environmental sciences

For a reference copy of the document with all sections, see nature.com/documents/nr-reporting-summary-flat.pdf

Life sciences study design

All studies must disclose on these points even when the disclosure is negative.

Sample size	1,000 repeated stochastic simulations per scenario were performed as this was considered sufficient to calculate measures of central tendency (mean) and probability of elimination (on a scale of 0 to 100 percent with zero decimals).
Data exclusions	Not applicable; all simulated data were included in the final analyses and are presented in the main manuscript and/or supplemental files.
Replication	Reproducibility is facilitated by open-source publishing of the software code, including highly detailed code documentation. The model predictions and the code to generate these are made available via an open access link in the manuscript.
Randomization	Not applicable; no real-life subjects (human or non-human) were randomized as the study was a computer simulation study.
Blinding	Not applicable; no blinding was performed as the study was a computer simulation study.

Reporting for specific materials, systems and methods

We require information from authors about some types of materials, experimental systems and methods used in many studies. Here, indicate whether each material, system or method listed is relevant to your study. If you are not sure if a list item applies to your research, read the appropriate section before selecting a response.

Materials & experimental systems

n/a	Involved in the study
☒	☐ Antibodies
☒	☐ Eukaryotic cell lines
☒	☐ Palaeontology and archaeology
☒	☐ Animals and other organisms
☒	☐ Clinical data
☒	☐ Dual use research of concern
☒	☐ Plants

Methods

n/a	Involved in the study
☒	☐ ChIP-seq
☒	☐ Flow cytometry
☒	☐ MRI-based neuroimaging
